# Colombian Contributions Fighting Leishmaniasis: A Systematic Review on Antileishmanials Combined with Chemoinformatics Analysis

**DOI:** 10.3390/molecules25235704

**Published:** 2020-12-03

**Authors:** Jeysson Sánchez-Suárez, Freddy A. Bernal, Ericsson Coy-Barrera

**Affiliations:** 1 Bioprospecting Research Group, School of Engineering, Universidad de La Sabana, Chía 250001, Colombia; jeyssonsasu@unisabana.edu.co; 2 Bioorganic Chemistry Laboratory, Universidad Militar Nueva Granada, Cajicá 250247, Colombia; Freddy.Bernal@hki-jena.de

**Keywords:** leishmania parasites, leishmanicidal, neglected tropical diseases, chemoinformatics, machine learning, Colombia, *Leishmania panamensis*

## Abstract

Leishmaniasis is a parasitic morbid/fatal disease caused by *Leishmania* protozoa. Twelve million people worldwide are appraised to be currently infected, including *ca.* two million infections each year, and 350 million people in 88 countries are at risk of becoming infected. In Colombia, cutaneous leishmaniasis (CL) is a public health problem in some tropical areas. Therapeutics is based on traditional antileishmanial drugs, but this practice has several drawbacks for patients. Thus, the search for new antileishmanial agents is a serious need, but the lack of adequately funded research programs on drug discovery has hampered its progress. Some Colombian researchers have conducted different research projects focused on the assessment of the antileishmanial activity of naturally occurring and synthetic compounds against promastigotes and/or amastigotes. Results of such studies have separately demonstrated important hits and reasonable potential, but a holistic view of them is lacking. Hence, we present the outcome from a systematic review of the literature (under PRISMA guidelines) on those Colombian studies investigating antileishmanials during the last thirty-two years. In order to combine the general efforts aiming at finding a lead against *Leishmania panamensis* (one of the most studied and incident parasites in Colombia causing CL) and to recognize structural features of representative compounds, fingerprint-based analyses using conventional machine learning algorithms and clustering methods are shown. Abstraction from such a meta-description led to describe some function-determining molecular features and simplify the clustering of plausible isofunctional hits. This systematic review indicated that the Colombian efforts for the antileishmanials discovery are increasingly intensified, though improvements in the followed pathways must be definitively pursued. In this context, a brief discussion about scope, strengths and limitations of such advances and relationships is addressed.

## 1. Introduction

Leishmaniasis is a vector-borne parasitic zoonosis caused by protozoa of the genus *Leishmania*, which is considered as an important neglected tropical disease (NTD). Clinically, this disease is classified as cutaneous (CL), mucosal (ML), or visceral leishmaniasis (VL). Central and South America are among the most affected regions, registering an annual incidence of 54,950 cases between 2001 and 2018 [1]. Among the 18 American countries where leishmaniasis is considered an endemic disease, Brazil, Colombia and Peru are those with the highest number of cases, involving 16432, 6362 and 6321 respectively, for 2018 [1]. In Colombia, the incidence rate of this disease was 26.2 cases per 100,000 population, with 98.6% of the cases related to CL [2]. Such an incidence was due to the presence of several parasites species, including *L. venezuelensis*, *L. equatorensis, L. lainsoni, L. colombiensis, L. mexicana, L. amazonensis, L. infantum, L. guyanensis, L. braziliensis* and *L. panamensis* [3,4,5,6], with the last three *Leishmania* species being the most representative etiological agents [4,6].

Despite the efforts involved in the development of new chemotherapeutic options/alternatives, the use of pentavalent antimony compounds still remains the first-line treatment today [7]. These drugs are recognized by their side effects [8,9,10], which implies an additional effort to monitor patients under treatment [11]. Moreover, the increasing number of therapeutic failure (mainly associated with parasite drug resistance) [12,13] establishes the need to persist in the search for more effective and safer antileishmanial agents. In this context, the accumulated knowledge about leishmaniasis pathophysiology, parasite biology and advancements in high-throughput screening, big-data analysis, analytical platforms, extraction/isolation and organic synthesis, open up new opportunities regarding the fight against NTDs such as leishmaniasis.

In 2013, the state-of-the-art regarding leishmaniasis research in Latin America showed that Brazil and Colombia were the most-contributing countries. However, Colombia’s scientific production was far from that of Brazil (almost six-fold lower production) [14]. After our current search, we found that this scenario has not practically changed, due to the fact that the burden/liability has been assumed by a small set of research groups basically disconnected from industry partners. Despite this, Colombian research on antileishmanials has led to the discovery of interesting chemical entities (both from natural and synthetic origin) with prospective activity against promastigotes and/or amastigotes of different *Leishmania* species. Their results have separately demonstrated high potential involving possible hits, but a holistic and comprehensive overall view of the outcome of those studies remains to be uncovered. Such a view would allow understanding/delineating the current status and future directions on further drug discovery-based initiatives against *Leishmania* parasites.

Increasing efforts are constantly paid by researchers around the world to improve the current drug discovery pipelines. Hence, the advances of computational methods and their application to them constitute a significant component. Accordingly, the use of novel and better algorithms have led to a large number of publications showing the extent of their applicability in computer-aided drug design projects [15,16,17]. The impact of chemoinformatics, understood nowadays as a discipline intersecting chemistry and computer science [18], has been moreover boosted by the development of machine learning algorithms in recent years [19,20,21]. Its use has extended across all levels of a typical drug discovery pipeline. 

The so-called in silico methods have been thoroughly applied to a wide range of scientific problems, including the search for new treatments against infectious diseases, and more specifically, NTDs such as leishmaniasis, as extensively reviewed [22,23,24,25]. Chemoinformatics has also greatly influenced the renascence of natural products, not only as a tool for identification of their vast biological potential but also aiming to fight NTDs [26,27,28,29,30,31]. Hence, a notorious use of chemoinformatics within the forthcoming Colombian research projects focused on antiparasitic agents is no less than expected.

As an endeavor to describe and characterize the status to date of the antileishmanial-focused Colombian studies, we present herein a systematic and comprehensive review of Colombian studies that have performed in vitro leishmanicidal trials. An approach to the chemical space conformed by the compounds involved in those research projects is disclosed for the first time. Finally, machine learning models are established for those compounds acting on amastigotes of *L. panamensis* (causative agent of CL in Colombia), including a particular emphasis on their interpretability and its relationship with important structural features.

## 2. Results and Discussion

### 2.1. Study Characteristics

**Figure 1:**
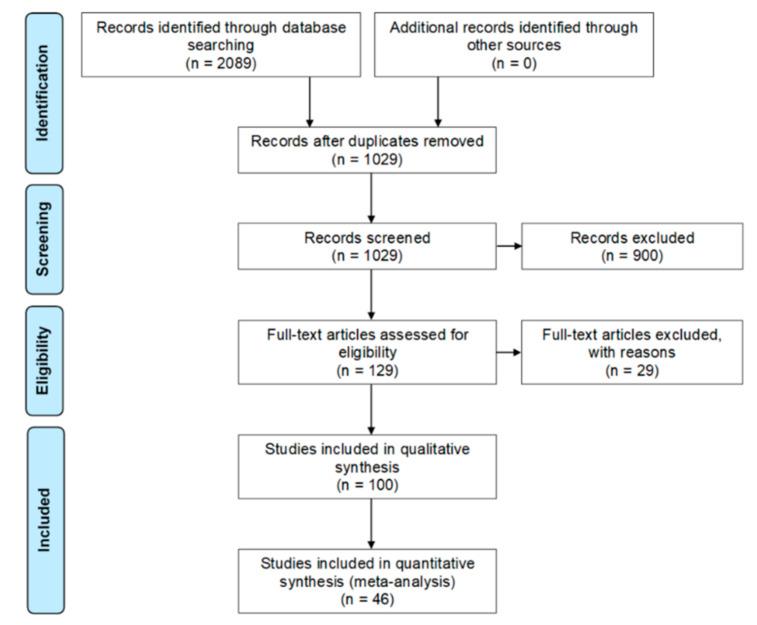
PRISMA flow diagram of this systematic review. Adapted from Moher et al. [32]. Compliance with the items in the statement guideline is presented in Supplementary Table S1.

The systematic review was performed under the Preferred Reporting Items for Systematic Reviews and Meta-Analyses (PRISMA) statement guidelines: the flowchart is outlined in Figure 1. After removal of duplicate studies, 1029 articles were screened and 900 (87.46%) were then excluded. At this point, we intended to delineate how the leishmaniasis research has been approached in Colombia. Hence, we classified the excluded studies within the following categories: entomology (vector studies), therapeutics (e.g., pharmacokinetics/pharmacodynamics studies), parasite biology, disease pathophysiology, epidemiology, diagnosis, case report, miscellaneous (i.e., studies with mixed goals and those with goals outside the previously described categories) and unrelated (i.e., those studies that, although were retrieved by the search, actually did not focus on the leishmaniasis subject). Most of the excluded papers were centered on entomological approaches (22%), followed by the ones with therapeutics goals (18%). The full results of this classification are shown in Supplementary Figure S1. Regarding the 129 papers that passed this phase, each one was read and analyzed at the full-text level (access to all the selected papers was granted) and 29 of them were afterward excluded according to inclusion/exclusion criteria, which resulted in 100 studies for the qualitative synthesis. In this phase, we could identify that 46 studies provided outputs comparable to each other, which enabled performing a quantitative synthesis. 

### 2.2. General Findings

**Figure 2:**
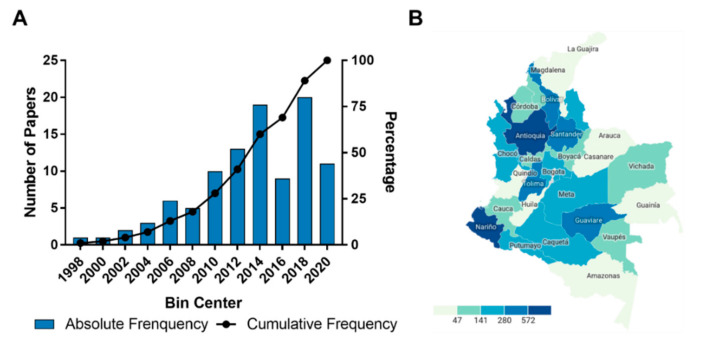
Evolution of the included scientific publications and geographical distribution of reported leishmaniasis cases in Colombia. (**A**) Time course of publications showing absolute (left-hand, *y*-axis) and cumulative frequency (right-hand, *y*-axis). (**B**) Number of reported cutaneous leishmaniasis (CL) cases for 2019 distributed through the political-administrative Colombian regions [39].

Publications showed an increasing trend between 1998 and 2020 (Figure 2A) as an indicator of the research importance on the discovery of new antileishmanial compounds. Remarkably, one institution leads the academic production in this field (*ca.* 40%), despite the wide distribution of leishmaniasis through several regions within the country (Figure 2B). Owing to the importance of this disease as a public health problem in Colombia, this fact calls for the local investment in other regions to improve the research capabilities on antileishmanials, even more considering the positive effect by the drug development from public research on the burden of neglected diseases [33]. It is worth noting that, despite that leishmaniasis’ global burden has noticeably decreased between 2007 and 2017, CL—the most prevalent leishmaniasis form in Colombia—and ML did not follow this trend, showing a significant disability-adjusted life year (DALY) rate increase (31.5%) in the same period [34]. The above-mentioned events and the therapeutic failure-related drawbacks of current antileishmanial drugs justify the persistent need for the discovery/development of more efficient and safer chemotherapy to treat CL. However, such a pipeline is generally prolonged (*ca.* 10 years) and very expensive (a general cost among $1.4–2.9 billion) [35,36], generating a clear imbalance between the required investment and the local budget, which is a common limitation in developing countries. The condition in the case of NTDs-related research is even worse, since no big pharmaceutical company is committed to participate in an antileishmanial discovery program due to the unattractive incentives to cover the costs for the development of drugs against NTDs [37]. An alternative is related to promote effective partnerships with non-profit organizations, such as Drugs for Neglected Diseases *initiative* (DND*i*) as a well-known example, whose modus operandi has covered those gaps to drive various compounds into lead optimization and pre-clinical phases [38].

**Figure 3:**
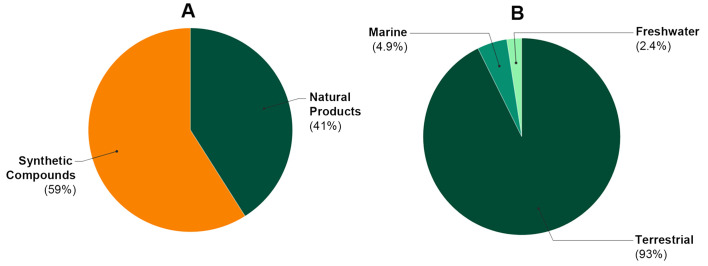
Classification of evaluated substances and environments explored for the research of antileishmanial agents in Colombia. (**A**) Publications classified according to the type of substance tested. (**B**) Publications evaluating natural products subdivided into the habitat source.

Although Colombia is a megadiverse country, most of the evaluated substances were obtained from synthetic approaches (Figure 3A). Regarding natural products, they were mostly obtained from terrestrial organisms (93%), while aquatic ecosystems (both marine and freshwater) remain as unexplored habitats (Figure 3B). Most of the studies (82.9%) used plants as a source of substances to be evaluated in antileishmanial assays, but the number of such records seems to be very low in comparison to other countries. These deeds call into question the efficiency of the natural products research in terms of antileishmanials discovery. Hence, a lack of organized/systematic programs, appropriately funded and directed to explore the potential of Colombian biodiversity under collaborative research networks, was evidenced.

In addition, other sources should also be examined to look for novel, effective leishmanicidal agents. For instance, marine organisms have been considered as promising suppliers of compounds with novel structures and noteworthy antiparasitic activity [40,41,42], including antileishmanial properties [43,44,45]. In this sense, it may be worthwhile considering marine specimens in future studies, especially marine microorganisms, whose chemical composition and biological activity—to our knowledge—remain to be deeply explored. Among the studies employing natural products, 22 evaluated the antileishmanial activity of crude extracts or solvent/chromatographic fractions (Table 1). Activity of the ethanol extract of *Bomarea setacea* aerial part can be highlighted, since it exhibited half-maximal effective concentrations (EC_50_) between 4.9 and 5.1 μg/mL against *L. amazonensis, L. braziliensis* and *L. donovani* promastigotes [46]. Only four studies showed promising activities against the clinically relevant stage of the parasite (i.e., intracellular amastigotes), comprising EC_50_ values under 10 μg/mL [47,48,49,50]. From those studies, the dichloromethane extract from leaves of *Conobea scoparioides* was found to be the most promising extract (EC_50_ = 1.30 μg/mL, selectivity index = 48.8) against *L. panamensis* [47]. This plant has been traditionally used for the treatment of leishmaniasis in Colombia [47]. However, despite the encouraging result, we did not find post-studies about the putative antileishmanial compounds isolated from *C. scoparioides.*

**Figure 4:**
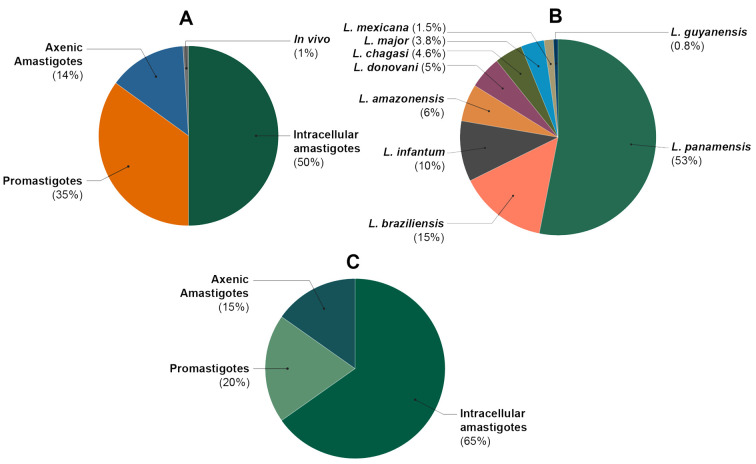
Characteristics of antileishmanial assays reported in those papers included in the present review. (**A**) Distribution of antileishmanial in vitro (subdivided into parasite forms, i.e., intracellular amastigotes, promastigotes and axenic amastigotes) and in vivo assays. (**B**) Distribution of *Leishmania* species involved in the antileishmanial in vitro assays. (**C**) Distribution of parasite forms used in antileishmanial in vitro assays against *L. panamensis.*

Antileishmanial assays can be performed on different *Leishmania* species as well as parasite forms as experimental models, and this fact limits the comparison possibilities owing to the differential response of the test parasite. Thus, in order to know the distribution of *Leishmania* species and forms within the group of included papers, we examined the experimental model employed in the assay. As expected, the main model used to evaluate the antileishmanial potential was intracellular amastigotes (50%), followed by the evaluation on promastigotes (35%) (Figure 4A). Regarding *Leishmania* species, most of the studies were conducted on *L. panamensis* (Figure 4B). This fact can be considered reasonable due to the fact that *L. panamensis* is the etiological agent most frequently reported in Colombia [3,6]. However, recent studies reported that *L. braziliensis* and *L. guyanensis* have relevant epidemiological reports and wide spatial distribution in Colombia [68,69]. Considering Colombia as a country with a significant number of pathogenic *Leishmania* species in circulation, these facts are very critical, bearing in mind that parasite sensitivity to antileishmanial agents relies on *Leishmania* species [70,71]. For instance, a study evaluated dehydroabietylamine derivatives and found species-dependent susceptibility for several of them [72], being consistent with other reports [73,74]. Indeed, owing to the role of intra- and inter-species *Leishmania* susceptibility [71,75,76], the incorporation of drug-resistant strains during antileishmanial research programs have been strongly recommended [77]. Presumably, the availability of high-throughput infection models has limited the Colombian research using other *Leishmania* species, since we found that 43.0% of the studies involved the green fluorescent protein (GFP)-transfected *L. panamensis* strain [78], representing 61.0% of such studies. 

Additionally, we discerned that most of the reviewed basic studies were initiated from an exploratory basis, starting from in vitro studies to find bioactives against one or two *Leishmania* parasites and using synthetic compounds or natural substances (i.e., crude extracts, solvent/chromatographic fractions and isolated compounds). Some studies (<10%) continued in order to involve in vivo trials and/or expand the structural alternatives by synthesizing more compounds with related structures. However, there is still a lack of studies continuing down to further development, since none of the tested compounds have entered biomedical or clinical phases, possibly due to budget and/or continuity issues or disconnection with pharmaceutical partners whose financial capacity and/or infrastructure might impulse such a kind of innovative, required solutions.

On the other hand, a set of 836 compounds were retrieved from 75 articles. We have mined 1060 records from these papers (Table 2), since some studies involved evaluations against different *Leishmania* species, even different antileishmanial models (i.e., promastigotes, axenic amastigotes and intracellular amastigotes). However, intracellular amastigotes were found to be the most used antileishmanial assay model (65%, involving 46 articles) among in vitro trials against *L. panamensis* (Figure 4C). This valuable information was further exploited for the first time to perform a chemoinformatics-based analysis using these leishmanicidal results (as EC_50_ values).

### 2.3. Chemoinformatics Analyses on Retrieved Compounds

**Figure 5:**
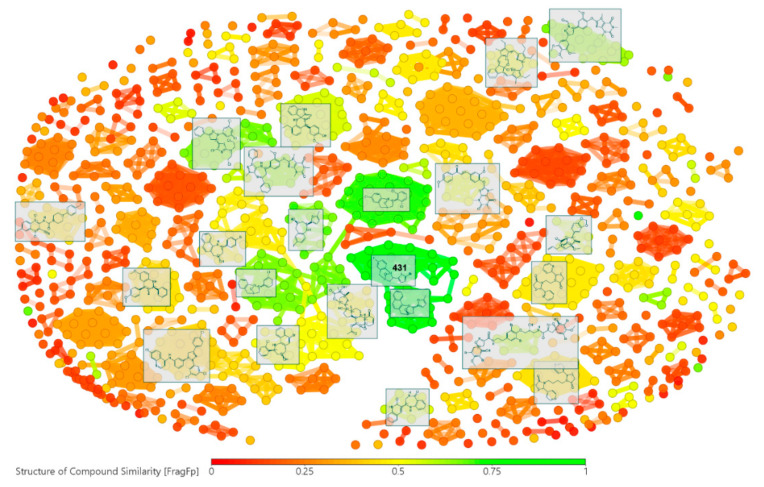
Similarity chart of the custom-made library. This plot was obtained after structure similarity analysis using the substructure fragment dictionary-based binary fingerprint descriptor (FragFp) [79]. The library comprised 836 compounds retrieved from the systematic review-derived information about Colombian research on antileishmanials. Structures of some compounds (enclosed in boxes) for those clusters possessing FragFP ≥ 0.4 were arbitrarily selected to illustrate the compound subset, according to the detailed information presented in Supplementary Table S2. Compound **431** was randomly designated as the reference compound to calculate the FragFp descriptor.

A custom-made library was then compiled from the systematic review-derived records. Such a library gathered 836 compounds, containing 90.3% synthetic compounds and 9.7% natural products. Activity and structural details (as EC_50_ in µM and Simplified Molecular-Input Line-Entry System (SMILES) codes, respectively) of these compounds can be found in Supplementary Table S2. The chemical space of the whole compound set was firstly examined in order to perform a structural filtering and qualitative characterization according to structural fragments/scaffolds. Therefore, a preliminary structural similarity analysis between test compounds was performed using the FragFp descriptor available in DataWarrior [79]. Compound **431** (having a linked thiophen-epoxybenzo[*b*]azepine moiety) was arbitrarily selected as a reference compound to calculate such a descriptor. The resulting similarity chart is presented in Figure 5. After such an analysis, some clusters were revealed with FragFp values, according to the heatmap based on the structure similarity index, between 0 (red = very different) and 1 (green = very similar). Such a scale indicated that our custom-made library gathered compounds related to 53 different FragFp-derived main clusters, which comprise small subsets (covering 6–23 compounds) of very structurally similar compounds depending on compound origin (e.g., the synthetic approach or natural source used in the respective study). Hence, several attempts to study/model a statistically validated structure–activity relationship were unsuccessful. However, this structure similarity analysis clustered the test compounds into several classes with FragFp ≥ 0.4, indicating that the library involves particular scaffolds that deserve a more robust analysis.

**Figure 6:**
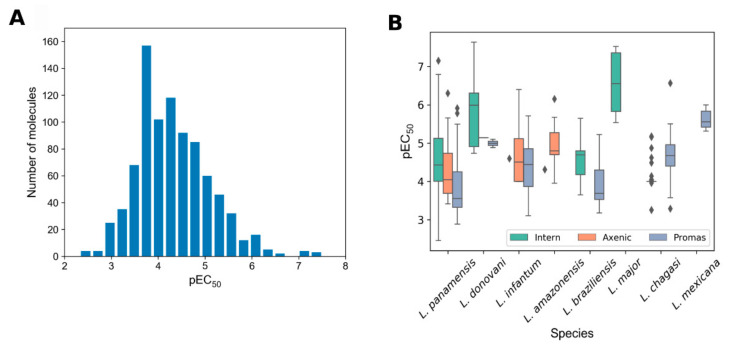
Antileishmanial activity of pure compounds expressed as pEC_50_. (**A**) Number of compounds associated with activity ranges and (**B**) distribution of activity according to parasite species and form.

Regarding the antileishmanial activity of retrieved compounds (Table 2), Figure 6A shows the general distribution of activity values for pure compounds expressed as negative decadic logarithm of the half-maximal effective concentration in mol/L(pEC_50_). As can be seen, despite the relatively large number of publications included, most of the tested compounds from these Colombian campaigns are rather poorly active. Only a limited fraction (18%) of the compounds displays EC_50_ values in the range 1–10 μM (5 < pEC_50_ < 6), whereas the number of substances with activity in the sub-micromolar range (pEC_50_ ≥ 6) is below 5%. This observation let us infer that the efforts made so far to find antileishmanial agents in Colombia should be considerably strengthened, if actual positive results are wanted. Otherwise, the studies will remain belonging to exploratory-oriented basic science rather than needed/applied medicinal and natural product chemistry projects.

As mentioned before, several *Leishmania* species have been included within the considered reports. Analysis of the activity of the studied compounds by *Leishmania* species (Figure 6B) reinforces the previously discussed necessity of further and more efficiently driven projects (as observed, most of the boxes appeared in a range of pEC_50_ between 4 and 6). Interestingly, there are some compounds with remarkable activity against intracellular amastigotes of *L. major*. Similarly, some compounds have displayed exceptionally high activity against intracellular amastigotes of *L. donovani* and *L. panamensis* (mainly found within the whiskers zone of the corresponding distribution). Following the well-recognized criteria for hit compounds for infectious diseases (including VL) [80], solely those compounds with EC_50_ below 10 μM could be considered as hits. Nonetheless, lack of information regarding structure–activity relationships, tractability of the chemotype, conformity with the rule of five and selectivity (>10-fold) may be considered as the major issue to define them as truly hit compounds.

#### 2.3.1. Chemical Space of Selected Compounds

The elevated number of papers determining activity against *L. panamensis* might be seen as directly related to the high incidence of reported cases of infection by this species in Colombia, as mentioned earlier. Thus, we decided to focus our attention on the compounds described therein, looking for possibly relevant structural information that could be used as a first-line tool for further investigations. The dataset was therefore filtered, keeping only entries for pure compounds that were tested against *L. panamensis*. More specifically, and in order to reach some degree of comparability, exclusively those entries with activity determinations on intracellular amastigotes were used in the subsequent analyses. This form of the parasite/model was preferred over axenic amastigotes and promastigotes within the publications (see above), supporting our decision for deeper examination. A total of 484 compounds were considered for further studies, comprising 9.1% natural products and 90.9% synthetic compounds.

**Figure 7:**
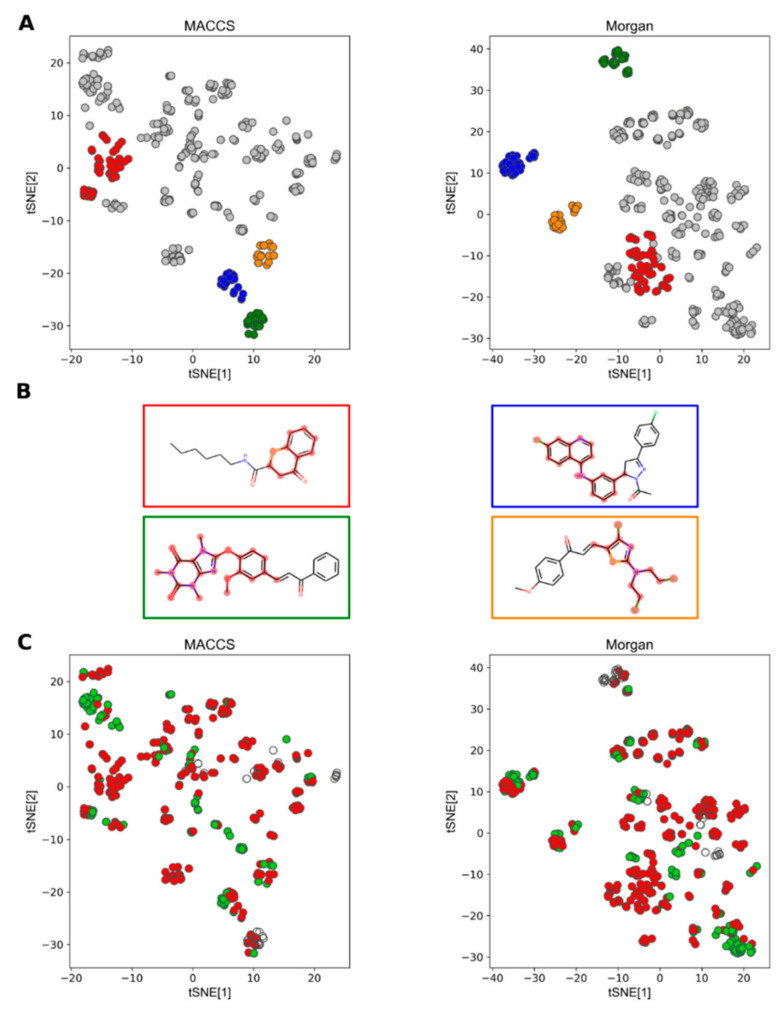
Representation of the chemical space of compounds tested against intracellular amastigotes of *L. panamensis*. (**A**) t-distributed Stochastic Neighbor Embedding (t-SNE) plot showing four common clusters to Molecular ACCess System (MACCS) (left) and Morgan (right) fingerprints, highlighted by red, blue, orange and dark green dots. Light gray dots represent the rest of the compounds. (**B**) Chemical structures of representative compounds of each selected cluster (enclosed in boxes colored according to the previous plot) with maximum common substructure (MCS) highlighted by pink contours. (**C**) t-SNE plot with dots colored by activity. Green dots: active compounds (pEC_50_ ≥ 5), red dots: intermediate and poorly active compounds (pEC_50_ < 5), empty dots: compounds without EC_50_ determination.

Structural information of the resulting group of compounds was extracted by using the Molecular ACCess System (MACCS) keys (167 bits) [81] and the Morgan fingerprints (radius = 2, 1024 bits) [82] implemented in RDKit [83]. These two sets of general molecular fingerprints were used aiming at a general glance of the corresponding chemical space. Owing to the typically high performance of t-distributed Stochastic Neighbor Embedding (t-SNE) as a dimensionality reduction method [84], we decided to look for possible compound clustering using such an algorithm and the fingerprints as independent inputs (Figure 7A). Although not directly comparable, both fingerprints offered some degree of clustering after t-SNE. Principal component analysis (PCA) was not able to represent clusters of similar compounds in a simple representation (data not shown). The partial generation of clusters of compounds by t-SNE would indicate actual structural relations among them, at least to some extent, as described by each fingerprint, i.e., some structural features seem to appear reiteratively within some groups of compounds. However, the limited clustering (high dispersion) also indicates a quite significant structural diversity within the whole group of compounds (wide chemical space). Hierarchical clustering analysis (HCA) proved to be consistent with the spatial distribution provided by t-SNE (see Supplementary Figure S2).

After exhaustive analysis of the clusters obtained by HCA and extraction of the corresponding maximum common substructure (MCS), only three of the clusters were completely coincident between fingerprints, whereas a fourth was partly in agreement (the MCS from the cluster using Morgan fingerprint was part of a larger substructure found when using MACCS keys). This result highlights the well-known differences among fingerprints, which would translate to changes in outcomes coming from direct comparisons of fingerprints. The four common clusters are depicted in Figure 7A by different colors.

Representative compounds from each cluster are also included in Figure 7B, whose MCSs are highlighted. As expected, the relative location of each common cluster in the scatter plots is different. However, it is noteworthy to mention that three of them are quite well separated from the others, suggesting very particular features compared to the rest of the compounds. Interestingly, the compounds in the cluster in red were not particularly separated from other compounds compared to those previously mentioned. The seemingly marked lack of resolution of this particular cluster (especially when using Morgan fingerprints) might be due to high structural diversity of its compounds, whereupon the fingerprint features would be rather strongly shared (overlapped), ending up with many common bits with other clusters.

Looking for insights into the possible effect of structural diversity on the antileishmanial activity, the t-SNE-derived scatter plots were colored by activity threshold (actives in green, pEC_50_ ≥ 5.0; Figure 7C). It is evident that the most active compounds are not concentrated in any specific cluster, i.e., none of the scaffolds so far analyzed in Colombian studies are clearly favored over the others. Particularly, the cluster in red (Figure 7A) is mainly constituted by poorly active compounds (red in Figure 7C), while it is difficult to establish the potential of compounds in the cluster in green (Figure 7A) owing to the lack of EC_50_ determinations for some of them (empty circles in Figure 7C).

#### 2.3.2. Machine Learning

After filtering off entries whose EC_50_ determinations were not available (e.g., only biological determination of percentage of inhibition at specific compound concentrations reported), a final set of 428 compounds was obtained. Owing to the inherent structural similarities among some compounds but also the huge differences in other cases (as shown above), and to the restricted capacity of the linear algorithms to provide reliable models (as mentioned before), machine learning was selected as a tool to analyze this dataset. Two different extensively used supervised learning algorithms were chosen to accomplish the task: random forest (RF) and support vector machines (SVM). Both MACCS and Morgan fingerprints were independently used for building the models. Preliminary evaluation of the classification variants of the selected algorithms showed decent performance (data not shown) and encouraged us to use actual activity values rather than an arbitrarily defined categorical dependent variable. Having decided for regression models, a coarse-to-fine scheme was followed for the optimization of the corresponding hyperparameters. In case of RF models, the number of trees in the forest, the minimum number of samples required to be at a leaf node, the minimum number of samples required to split an internal node, the maximum number of features to consider for the best split and the number of samples to draw from the training set during bootstrap were considered for optimization. The dataset was randomly split into training and test set (80:20%), ensuring maximum coverage of activity range for the latter. For both fingerprints, models offered maximum performance using 1 sample as a minimum to be at a leaf node and the total number of features as maximum. Those models were named M1 and M2, for MACCS and Morgan, respectively. While M1 used 306 trees, 7 samples to split a node and 75% of samples drawn during bootstrap, M2 required 127 trees, 2 samples and 94% of the samples, respectively.

In case of SVM models (M3 and M4 for MACCS and Morgan, respectively), the optimization was performed considering variations in the kernel functions, the kernel coefficient, the epsilon-tube and the regularization parameter C. The optimized models made use of the Radial Basis Function (RBF) kernel. The best performance for M3 was achieved with epsilon = 0.1, C = 2.5 and gamma = 0.04. M4 performed better when it used the combination of hyperparameters epsilon = 0.08, C = 2.8 and gamma = 0.05.

All the models were trained and tested for predictivity using ten repetitions. Table 3 shows the corresponding validation parameters as a mean of the ten runs. As can be seen, the generated models offered barely acceptable cross-validation (CV) scores, with limited prediction power. Nonetheless, M1–M4 outperformed classical linear models. The limited robustness for M1–M4 was not less than expected coming from such a diverse dataset. It is impossible to properly ensure comparability of activity data due to presumable changes in the specific procedures, despite using the same parasite forms/models (i.e., not all the compounds were experimentally tested at the same time and under the same exact conditions or not even in the same laboratory). Moreover, we did not take into account the implicit data error (which is sometimes not adequately informed, either), whose impact on computational modeling was already highlighted long ago [85]. It must be noted however that the data error is still not included in most of the Quantitative Structure-Activity Relationships/Quantitative Structure-Property Relationships (QSAR/QSPR) studies published in scientific journals. Regardless, both algorithms provided comparable results in terms of internal and external validation (Table 3). 

Although RF using Morgan (M2) fingerprints displayed significantly higher R^2^ than that from MACCS (M1) during training, both internal and external validation coefficients were indistinguishable between models. In the case of SVM, the use of Morgan fingerprints (M4) demonstrated better predictability of the external set of compounds, albeit comparably low performance during CV. Beyond the isolated statistical values, and in spite of their closely related performance, M2 afforded the lowest deviations in predicted antileishmanial activity, represented by the lowest dispersion of data points around the regression line between experimental and predicted values (Supplementary Figure S3; all the corresponding activity predictions are included in Supplementary Table S3).

**Figure 8:**
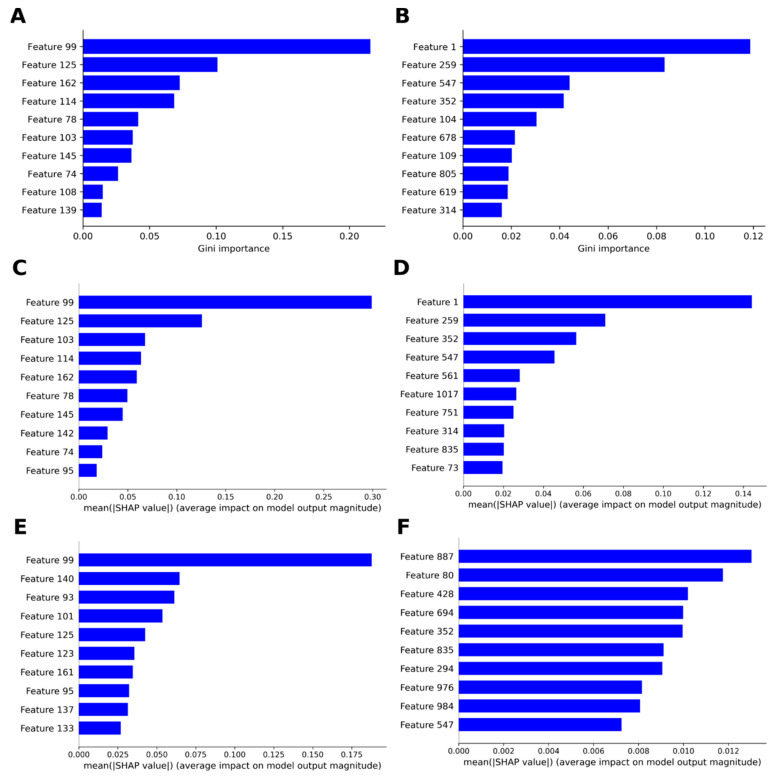
Relevant structural features for machine learning models. (**A**) Gini importance for M1, (**B**) Gini importance for M2, (**C**) SHapley Additive exPlanations (SHAP) values for M1, (**D**) SHAP values for M2, (**E**) SHAP values for M3 and (**F**) SHAP values for M4. Only the ten top features are shown.

With the limited but still acceptable capacity of the obtained models, we were interested in deciphering the governing structure–activity relationships behind them. Although the machine learning algorithms are typically known for their black box nature, recent advances have been made in order to extract information regarding feature importance, like the use of the SHAP (SHapley Additive exPlanations) theory and the derived Shapley values [86,87]. Taken from game modeling, the SHAP theory helps to explain the contribution each single feature has on the outcome obtained. Implementation of this theory to gain detailed information from machine learning models has already been shown for drug discovery projects [88,89], making it possible to define the most important fingerprint bits contributing to the variance in activity. We applied the SHAP theory to the optimized models. In the case of RF models (M2 and M4), the Gini importance was also analyzed. The results are shown in Figure 8. There was an overall agreement between Gini and SHAP values for both M1 and M2, e.g., features 99 and 125 were consistently the top two in M1 (Figure 8A,C), whereas for M2 features, 1 and 259 appeared the most relevant by both methods (Figure 8B,D). High correlation between Gini and SHAP values have already been observed and reported [88]. Interestingly, the observed profound effect of feature 99 on the prediction of activity also prevailed in M3 (Figure 8E), suggesting some similarity between algorithms. The SHAP values also allowed inferring a significant effect of features 125 and 95 on M3 predictions, which were within the top ten features affecting M1 as well. This marked coincidence of features would indicate that both algorithms were able to identify basically the same structural features (held by the MACCS fingerprints) as responsible for the variance in activity. The SHAP values were also in agreement with the Gini importance values for M2, e.g., features 1, 259, 352 and 547 were ranked as the top four in both cases (Figure 8B,D). However, analysis of the corresponding SHAP values for M4 revealed a completely different distribution of features affecting the outcome of the model (Figure 8F). Only features 352 and 547 remained as part of the top ten, although with less importance. Being affected by several features at comparable costs implied that there was not any specific feature with a clear strong impact on M4 predictions, as it was observed above for the other models.

Detailed analysis of the definition of the MACCS keys with higher SHAP values revealed that both M1 and M3 relied on similar structural patterns overall. Features 99 and 125, found in both cases, and 162 and 101, being exclusive for each model respectively, are related to the presence of C = C and aromatic rings. For M1, feature 114, which represents the presence of ethyl units bound to any atom, was also important, while the presence of methyl groups bound to heteroatoms was relevant for M3 (feature 93). Particularly interesting was the fact that M3 predictions were affected by the number of oxygen atoms in the molecule (feature 140 for O > 3). In contrast, the presence of chlorine atoms (feature 103) was important for M1.

**Figure 9:**
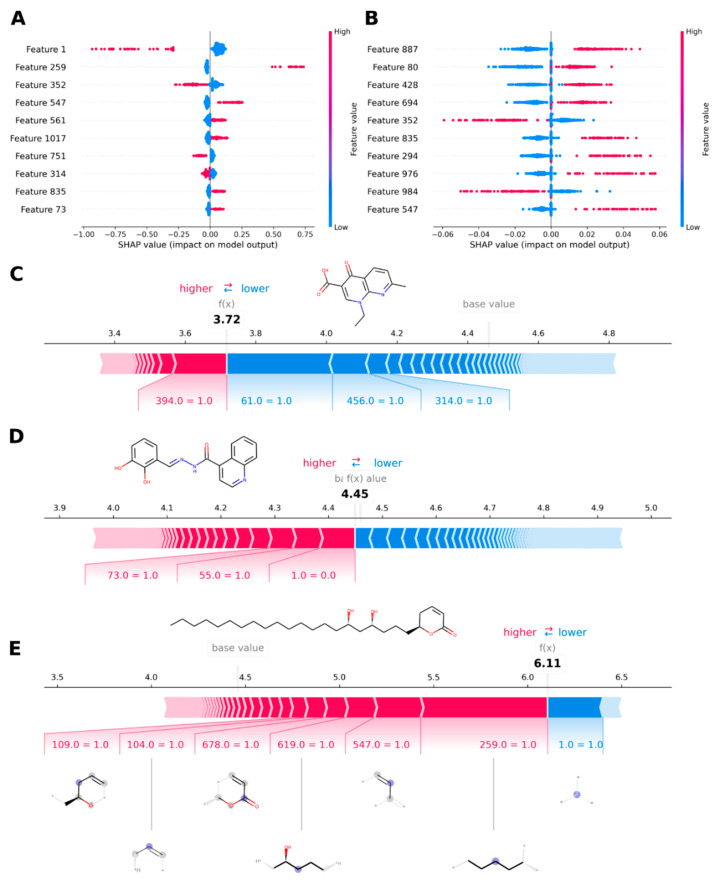
Contribution of structural features to M2 and M4. (**A**) SHAP values for M2 and (**B**) SHAP values for M4, colored by feature value (red: presence, blue: absence). (**C**) Force plot for a representative poorly active compound (**190**), (**D**) force plot for a representative compound with intermediate activity (**586**) and (**E**) force plot for a representative compound with high activity (**164**) as predicted by M2. Plot (**E**) includes the most important fingerprint bits affecting the prediction.

Analyzing the individual contributions of each feature to the general outcome for M2 showed that the presence of feature 1 in the compounds was deleterious for the activity (Figure 9A). A similar general result was observed for features 352 and 751, although at considerably lesser extent. In contrast, the presence of feature 259 significantly favored the predicted activity values. Features 547, 561 and 1017 are other examples of features positively contributing to the activity. A comparable analysis in case of M4 was not straightforward due to the already mentioned high number of features responsible for the activity. However, absence of features 352 and 984 seemed beneficial for the activity, whereas features like 887 and 835 appeared to play a positive role (Figure 9B).

A more comprehensive analysis of the underlying structure–activity relationships for the compounds in the present dataset is not practically achievable because of the strong structural differences among compounds. Nevertheless, several additional insights could be retrieved from in-depth exploration of the individual Shapley values. Thus, taking advantage of the likelihood of drawing Morgan fingerprints offered by RDKit, representative compounds with low (compound **190**), intermediate (compound **586**) and high (compound **164**) antileishmanial activity were further studied. Model M2 was chosen for this analysis based on its apparently low deviation in predictions and clearly outlined important features. Figure 8C–E shows the corresponding force plots for those compounds. It can be observed how the activity of the inactive compound (**190**, Figure 9C) is strengthened by the presence of feature 394, while features 61, 456 and 314 could be responsible for the low value as they are pushing it down. Surprisingly, only the latter feature is part of the top ten features affecting the general outcome of the model. On the other hand, the activity of compound **586** (Figure 9D) was apparently caused by the presence of features 73 and 55. Moreover, absence of feature 1 significantly contributed to the activity of this specific compound, too, being the most important feature for M2, as previously noted. In the case of the active compound (**164**, Figure 9E), the activity was dominated by the presence of several features, including 109, 104, 678, 619, 547 and 259. To make predictions, the model predominantly used most of those features. In addition, the presence of feature 1 in this compound decreased the predicted value, as expected from the general trend observed. Features 109, 547 and 678 correspond to the 5,6-dihydro-2*H*-pyran-2-one moiety (Figure 9E). Meanwhile, features 259 and 619 are related to the aliphatic chains in vicinity of the hydroxyl groups. Particularly, feature 1 in this compound structure refers to the hydroxylated chiral carbons. Presumably, M2 might have learned some effects on activity due to chirality of those stereocenters.

#### 2.3.3. Drug-Likeness Filtering

In order to get an idea of some interesting scaffolds to be considered in future investigations, further analyses were carried out on the group of active compounds. As a first step, their drug-likeness was partially assessed checking for the presence of undesirable moieties according to the filters for Pan-Assay INterference compounds (PAINs) implemented in the FAFDrug4 web server [90]. Despite that 85% of the active compounds passed the three filters available in the server, more than 60% of them might still be considered as potentially reactive substances containing groups susceptible to covalent binding (e.g., 23% of the active compounds contain Michael acceptor groups). Only a reduced set of twenty-eight compounds with confirmed activity on intracellular amastigotes passed the mentioned filters. However, more than half of these compounds (57%) showed compliance with the rule of five (Ro5) as well (one violation of the Ro5 was mainly found for the rest).

**Figure 10:**
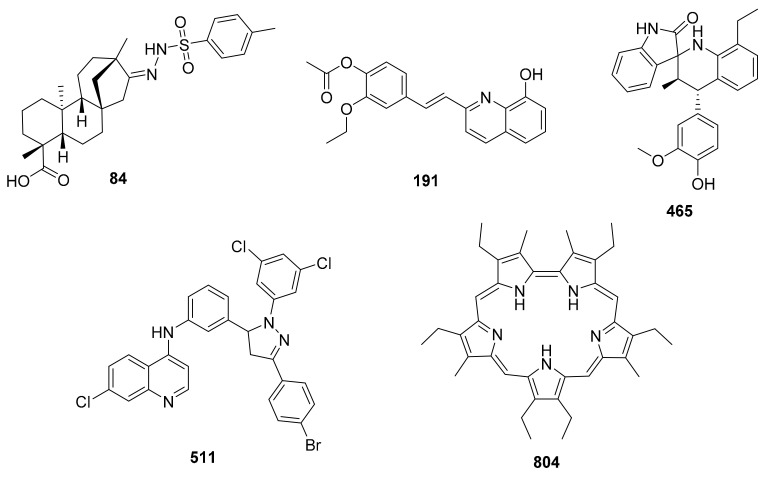
Chemical structures of the best antileishmanial candidates from Colombian campaigns.

On the other hand, selectivity index (SI), defined as the ratio between cytotoxicity and antileishmanial activity, was considered for the last filtering step. This process revealed that whereas sixteen compounds (57%) showed SI > 2, only three of them (11%) displayed actual interesting selectivity values as to be considered for further development (Table 4). Figure 10 shows some of the best candidates after the aforementioned filtering.

Interestingly, a similarity search in SciFinder revealed that the scaffolds comprised by the above-mentioned compounds (Figure 10) have been rather uniquely considered as antileishmanial agents in Colombian research projects. Thus, in spite of the somewhat common nature of most of those scaffolds, their specific combinations have not yet become part of other studies focused on antileishmanial substances. No additional reports were found for the combination of chloroquine and pyrazole scaffolds nor for the combination of indolinone and tetrahydroquinoline scaffolds (as in compounds **511** and **465**, respectively). Similarly, no further studies on leishmanicidal sulfonylhydrazides of beyerene- or stevioside-like diterpenes (e.g., **84**) have been published. On the other hand, compound **191** represents a group of substances (styrylquinolines) quite commonly included in medicinal chemistry projects. Nonetheless, studies on their potential as antitrypanosomatid agents has been limited as well. To the best of our knowledge, there is only one recent study focused on the leishmanicidal properties of a group of related compounds (4-aminostyrylquinolines) [91]. Decent activity against amastigotes of *L. pifanoi* and moderate selectivity indexes were therein reported. Additionally, assessment of the antileishmanial potential of alkenylquinolines was previously reported [92]. In this case, rather poorly active compounds were evinced, limiting the possible identification of interesting candidates. Seemingly, most of those compounds showed better antitrypanosomal activity.

## 3. Methods

### 3.1. Systematic Review

#### 3.1.1. Search and Eligibility Criteria

The search was carried out in Scopus, Web of Science, PubMed and Scielo databases (last search on 6 July 2020) using an appropriated search equation for each database with the keywords and Boolean operators as follows: leishmani* OR antileishmani* OR leishmanicid*. The search results were then refined according to the filter tools available in each database to select the documents affiliated to Colombia, excepting the Scielo database. In this database, the search equation was “(leishmani* OR antileishmani* OR leishmanicid*) AND (colombia)”, as this database does not provide a filter option by country affiliation. After data retrieval from databases, the inclusion criteria were defined for those articles containing the following characteristics/information: (1) original articles, (2) in vitro or in vivo antileishmanial activity, (3) assays with synthetic compounds, (4) assays with pure isolated compounds of natural origin and (5) tested crude extracts. Retrieved studies were excluded if they involved only known/recognized antileishmanial drugs, or if accessibility to full-text versions was not accomplished.

#### 3.1.2. Study Selection

The selection of studies was made in two phases [93]. First, the search results were uploaded to the Rayyan web application [94]. Two reviewers independently screened the titles according to the inclusion criteria. Articles marked as “included” by the two reviewers were promptly selected for the next phase. In the cases of unmatched marks (i.e., articles that were marked as “included,” “excluded” or “maybe” by only one reviewer), the papers were analyzed by the two reviewers; if the disagreement persisted, the final decision was made by the third author. Then, the full-text version of the initially filtered articles was read and selected applying the inclusion/exclusion criteria.

#### 3.1.3. Data Collection

A preliminary data collection form was built according to the review goals, and its suitability was evaluated in a pilot procedure with ten randomly selected papers. Then, the final version of the data collection form was used for the survey/examination of each paper that passed the title-screening phase. The three authors cured the resulting spreadsheet. Since the analysis and characterization of the reported antileishmanial compounds are one of the main objectives of this review, a chemoinformatics approach to the chemical space represented by the compiled compound library was further accomplished. This analysis was based on fingerprints using conventional machine learning algorithms and clustering methods.

### 3.2. Chemoinformatics Analysis

#### 3.2.1. Data Preparation

After compilation of the systematic review-derived antileishmanial records, the structures of retrieved compounds were individually sketched in MarvinSketch (ChemAxon, Budapest, Hungary) and converted into SMILES as a line notation for chemical structure. This notation uses the American Standard Code for Information Interchange (ASCII) character encoding. Once the custom-made library was completed, a structure filtering analysis was firstly performed using the substructure fragment dictionary-based binary fingerprint descriptor (FragFp), incorporated in DataWarrior v5.0.0 [95]. A structure comparison between compound sets can be achieved with this descriptor (analogous to Molecular Design Limited (MDL) keys), as it considers structural moieties through 512 predefined fragments into a dictionary [79]. Each fragment is contained into one bit of the FragFp descriptor; therefore, a bit is defined as 1 if a respective fragment occurred in the structure at least one time. Thus, a list of all dictionary fragments (as part of the substructure query) is generated and the overall, comparison led to the substructure filtering. This filtering is visualized through a similarity chart (e.g., a scatter plot) according to the FragFp index.

Compounds’ structures were additionally characterized by MACCS keys [81] and Morgan fingerprints [82] as selected molecular representations. MACCS keys consist of 166 bits accounting for either absence or presence of specific structural patterns. Morgan fingerprints encode for structural features on a radial basis, i.e., having a circular shape, and they account for structural fragments. In this work, a radius of 2 and length of 1024 bits were chosen. Both sets of fingerprints were calculated using RDKit 2020.03.1 [83], where Morgan fingerprints are defined as a modification of the extended connectivity fingerprints (ECFP) [96]. Activity data were transformed into the corresponding pEC_50_ (negative decadic logarithm of the EC_50_ in mol/L). Finally, the PAINs and Ro5-based filtering was accomplished using the FAF-Drugs4 web server [90]. 

#### 3.2.2. Chemical Space by t-SNE

t-distributed Stochastic Neighbor Embedding (t-SNE) is an unsupervised, non-linear technique that allows the visualization of high-dimensional data [84]. It works in three steps: first, similarities among samples in the high-dimensional space are defined, by measuring the corresponding probabilities using Gaussian distributions; secondly, similarities among samples in the low-dimensional space (typically two-dimensional (2D)) are calculated, but in this case using Student’s t-distribution with one degree of freedom (known as Cauchy distribution) instead. Finally, and in order to genuinely recreate the high-dimensional distribution, optimization of the distributions is conducted by gradient descent using Kullback–Liebler divergence as the loss function. The perplexity and maximum number of iterations were manually tuned. t-SNE implementation in Scikit-learn [97] was used in the present work.

#### 3.2.3. Random Forest

As a supervised machine learning algorithm, random forest (RF) makes use of groups of decision trees, and is therefore part of the ensemble learning methods [98,99]. Each tree is trained from a bootstrapped sample of data, where typically a random subset of features is considered for node splitting. The final predictions correspond to the average of all the predictions made from those individual learners. Node splitting is controlled by reduction of the Gini index (Gini “impurity”). The sum of the reduction in Gini impurity is termed as Gini importance [100]. The number of trees in the forest (5–1000), the minimum number of samples required to be at a leaf node (1–10), the minimum number of samples required to split an internal node (2–16), the maximum number of features to consider for the best split (total number of features, base 2 log of the total, and squared root of the total) and the number of samples to draw from the training set during bootstrap (5–95%) were subjected to optimization in this work. RF regression models were built using Scikit-learn.

#### 3.2.4. Support Vector Machines

Support Vector Machines (SVM) is another supervised machine learning algorithm, whose principle is to define hyperplanes for effective segregation of the data typically into classes of objects [101]. Those data points closest to the hyperplanes are called support vectors. The best hyperplanes are selected by minimization of the margin (gap between the support vectors delimiting it). Implementation of SVM usually requires so-called kernel functions that help finding the hyperplanes by increasing the dimensionality of the data (transformation from lower to higher dimensional space). The regularization parameter C trades off misclassification error and decision boundary (margin size). The kernel function (linear, polynomial, sigmoid and radial basis function (RBF)), the kernel coefficient gamma (1 × 10^–6^ to 1), the epsilon-tube (0.1–0.5) and the regularization parameter C (1–100) were optimized during the present work. SVM in regression models (SVR) were built with Scikit-learn.

#### 3.2.5. Hyperparameter Optimization

Both RF and SVM models were submitted to hyperparameter optimization in a coarse-to-fine approach. The process was carried out in two instances. The first one consisted of random sampling of the corresponding hyperparameter grid. The best combination of hyperparameters was selected based on the coefficient of determination obtained during a 5-fold cross-validation (CV) scheme. Afterwards, an exhaustive evaluation of hyperparameter combinations in the proximity of the best set detected in the previous step was performed. The same scoring function was used in the last step. The whole process was achieved applying the corresponding Scikit-learn implementations. 

#### 3.2.6. Final Models

Final RF and SVM regression models were built using the corresponding optimized hyperparameter sets. Each model was fitted ten times and predictions were obtained accordingly. Performance of the models was evaluated by the coefficient of determination (R^2^) and the mean absolute error (MAE) calculated for the respective activity predictions, and expressed as an average. 10-fold CV assessed internal validity of the models.

#### 3.2.7. Analysis of Contributions by SHAP Values

The concept of Shapley values was developed early in cooperative game theory [102], where the calculation of the contribution of each single player to the global outcome is highly important. Thus, properly rewarding each player, in order to provide a unique result prediction, is what the Shapley values represent. This theory was recently extended aiming at a measure of feature importance in different predictive models [86]. The introduced SHAP (SHapley Additive exPlanation) values help explain how the predicted output changes according to the appearance of any feature. Their application for machine learning models’ explanations has been proven [87], including their outstanding potential for machine learning-based drug discovery projects [88,89]. Computation of SHAP values was carried out using an open implementation under Python [87,103].

## 4. Conclusions

Although Colombia is one of the countries with more pathogenic *Leishmania* species in circulation, scientific research was found focused on *L. panamensis.* Since other species such as *L. braziliensis* and *L. guyanensis* are becoming significant etiological agents (associated with an important number of CL cases), these *Leishmania* species should be included in the forthcoming research programs on antileishmanial discovery. Furthermore, our findings highlight the need for involvement of more research centers, allowing strategic collaborations that can lead to more multidisciplinary approaches. Indeed, considering the CL burden in Colombia, along with the demand for more effective and safer chemotherapeutic options, it is essential that public investment (national and regional) maintain and even prolong/improve the effective financial support for the research on leishmaniasis control (particularly the development of antileishmanial agents). Pondering on studies involving the GFP-transfected *L. panamensis* strain, and the difficulties and challenges associated with the leishmanicidal screening assays, the development of more GFP-transfected species (e.g., *L. braziliensis*, *L. guyanensis*) would positively impact the antileishmanial-oriented studies. Regarding natural products, it was expected to find a higher number of studies. The internal regulations for accessing genetic sources has probably limited bioprospecting studies in this field. In this regard, this assumption should be exhaustively evaluated. In any case, we concluded that nature remains as an underexplored resource concerning its leishmanicidal potential and the limited number of studies show a biased attentiveness on plants. 

Moreover, a holistic analysis of the activity data retrieved from our systematic literature revision exposed that more than 50% of them have low to non-existent activity against *Leishmania* parasites. Consequently, future investigations on small molecules targeting this disease should be guided by medicinal chemistry principles using rational approaches. Additionally, the bottlenecks and gaps across the antileishmanials development pipelines can be overcome with public–private partnerships, combining knowledge from academia and infrastructure and financial support from pharmaceutical companies within an efficient and effective scientific and technical cooperation.

Profound chemoinformatics analyses indicated apparently high chemical diversity within the group of compounds with measured activity against intracellular amastigotes of *L. panamensis* (the largest available group of compounds). Interestingly, this chemical space could be condensed within a relatively small number of compound clusters without any visibly privileged scaffold. Furthermore, classical machine learning algorithms facilitated uncovering some underlying structure–activity relationships, affording a set of models with decent capacity to predict antileishmanial activity. Combination of these models with SHAP theory could be a valuable tool in further research to be developed in Colombia, as foreseeing the possible activity and associating it with SAR information might serve as a simple but effective initial guidance.

## Figures and Tables

**Table 1:** Summary of the antileishmanial potential of crude extracts retrieved from the articles included in this systematic review.

*Leishmania* Species ^a^	Parasites Form ^b^	EC_50_ (μg/mL) ^c^	E/F ^d^	Source ^e^	Ref ^h^
*L. braziliensis; L. infantum; L. panamensis*	Promastigote	N/A ^e^	1	*Annona spraguei*	[51]
*L. braziliensis; L. panamensis*	Promastigote	N/A ^e^	3	*Annona muricata*	[52]
*L. amazonensis; L. braziliensis; L. infantum; **L. panamensis***	Promastigote, **Intracellular amastigote**	1.30	88	*Conobea scoparioides*	[47]
*L. amazonensis; **L. braziliensis; L. donovani***	Promastigote	10.70	36	*Rollinia pittieri*	[53]
*L. amazonensis; L. braziliensis; **L. donovani***	Promastigote	4.90	26	*Bomarea setacea*	[46]
*L. braziliensis*	Intracellular amastigote	12.40	1	*Ervatamia coronaria*	[54]
*L. panamensis*	Intracellular amastigote	6.25	8	*Xylopia discreta*	[48]
*L. braziliensis*	Promastigote	17.40	13	*Rosmarinus officinalis*	[55]
*L. amazonensis*	Axenic amastigotes	9.00	94	*Renealmia alpinia*	[56]
*L. mexicana*	Axenic amastigotes	>50.00	452 ^f^	*Several ^g^*	[57]
*L. panamensis*	Intracellular amastigote	15.40	6	*Physalis peruviana*	[58]
***L. panamensis*** *; L. braziliensis; L. major; L. guyanensis*	Promastigote	42.23	10	*Origanum vulgare*	[59]
*L. panamensis*	Intracellular amastigote, **Axenic amastigotes**	38.50	6	*Piper daniel-gonzalezii*	[60]
*L. panamensis*	Intracellular amastigote	18.50	13	*Heliotropium indicum*	[61]
***L. panamensis*** *; L. major*	Promastigote, **Intracellular amastigote**	6.16	3	*Zanthoxyllum monophyllum*	[49]
*L. panamensis*	Intracellular amastigote	48.07	1	*Artemisia annua*	[62]
***L. braziliensis*** *; L. panamensis*	Promastigote, **Intracellular amastigote**	9.19	4	*Lippia alba*	[50]
*L. panamensis*	Intracellular amastigote	30.70	8	*Pilocarpus alvaradoi*	[63]
*L. braziliensis*	in vivo on golden hamsters	N/A ^f^	1	*Arnica montana*	[64]
*L. panamensis*	**Promastigote**, Intracellular amastigote	23.42	2	*Sarconesiopsis magellanica*	[65]
*L. panamensis*	Promastigote	N/A ^f^	4	*Galleria mellonella*	[66]
*L. infantum; **L. braziliensis***	Promastigote	47.70	12	*Enterobacter hormaechei*	[67]

^a^*Leishmania* species used in the antileishmanial assays. For studies involving more than one species, the most sensitive species are highlighted in bold font. ^b^ Parasite forms (i.e., promastigote, intracellular amastigote or axenic amastigote) employed in the antileishmanial assays. For studies involving more than one parasite form, the highlighted one in bold font indicates the result presented in this table. ^c^ Leishmanicidal half-maximal effective concentration (EC_50_). The lowest EC_50_ value reported in each study. ^d^ E/F = Number of Extracts/Fractions Assayed; ^e^ Scientific name of the source of the most active crude extract/fraction. ^f^ In these studies, the EC_50_ values were not calculated/informed. ^g^ This study screened several plants from different Latin American countries. None of the 17 Colombian screened plants were found to be active in the range of test concentrations. ^h^ Ref = Cited reference.

**Table 2:** Summary of antileishmanial activity of those compounds retrieved from the articles included in this systematic review.

Parasite Form ^a^	Antileishmanial Activity Category ^b^	Number of Records ^c^
Intracellular amastigotes	High	127
	Intermediate	68
	Low	221
	Not Determined	162
	Not Available	112
Axenic amastigotes	High	28
	Intermediate	27
	Low	37
	Not Determined	32
	Not Available	28
Promastigotes	High	29
	Intermediate	44
	Low	81
	Not Determined	50
	Not Available	14
**Total ^d^**		**1060**

^a^ Parasite form employed in the antileishmanial assay. ^b^ Categories according to the resulting negative decadic logarithm of the half-maximal effective concentration in mol/L (pEC_50_) for each compound: High = pEC_50_ ≥ 5.00 (EC_50_ ≤ 10.0 µM), Intermediate = 4.60 ≤ pEC_50_ < 5.00 (25.0 µM > EC_50_ ≥ 10.0 µM), Low = pEC_50_ < 4.6 (EC_50_ > 25.1 µM), Not Determined = compounds included into the respective study, but the EC_50_ value was over the maximum evaluated concentration, Not Available = compounds included into the respective study, but the antileishmanial assay did not return an EC_50_. ^c^ Number of records regarding those test compounds with a pEC_50_ value within the respective antileishmanial activity category and parasite form. ^d^ The raw data of this table is presented in Supplementary Table S2.

**Table 3:** Statistical performance of machine learning models.

Validation Parameter ^a^	M1 ^b,d^	M2 ^b,e^	M3 ^c,d^	M4 ^c,e^
R^2^_train_	0.813	0.925	0.847	0.849
MAE_train_	0.250	0.155	0.202	0.183
R^2^_CV_	0.621	0.621	0.600	0.592
MAE_CV_	0.359	0.354	0.370	0.358
R^2^_test_	0.670	0.666	0.583	0.689
MAE_test_	0.322	0.324	0.352	0.339

^a^ R^2^: coefficient of determination, MAE: mean absolute error, CV: 10-fold cross-validation. ^b^ RF = Random Forest. ^c^ SVM = Support Vector Machines. ^d^ MACCS = Molecular ACCess System. ^e^ Morgan.

**Table 4:** Selected candidates after Pan-Assay INterference compounds (PAINs) filtering.

Compound	Species	EC_50_^a^	Cellular Line	IC_50_^b^	SI ^c^	Ro5 ^d^ Violations
**3**	*L. panamensis*	4.03	U-937	15.6	3.9	0
**84**	*L. braziliensis*	2.26	U-937	6.99	3.1	1
**85**	*L. braziliensis*	2.53	U-937	8.75	3.5	2
**191**	*L. panamensis*	0.57	U-937	8.02	14	0
**192**	*L. panamensis*	7.48	U-937	17.7	2.4	0
**341**	*L. panamensis*	4.81	U-937	12.7	2.6	0
**343**	*L. panamensis*	6.18	U-937	21.3	3.4	0
**345**	*L. panamensis*	5.90	U-937	15.6	2.6	0
**465**	*L. braziliensis*	3.30	BMDM	724	91	1
**487**	*L. panamensis*	7.07	U-937	19.7	2.8	2
**489**	*L. panamensis*	3.70	U-937	9.62	2.6	2
**490**	*L. panamensis*	3.41	U-937	9.69	2.8	1
**499**	*L. panamensis*	6.50	U-937	16.7	2.6	2
**511**	*L. panamensis*	4.47	U-937	322	72	2
**566**	*L. panamensis*	5.51	U-937	13.1	2.4	1
**804**	*L. panamensis*	0.60	U-937	3.87	3.9	2

^a^ EC_50_ = half-maximal effective concentrations (expressed in µM) determined in intracellular amastigotes of the respective *Leishmania* species; ^b^ IC_50_ = half-maximal inhibitory concentrations (expressed in µM) determined in human monocytes (U-937) or bone marrow-derived macrophages (BMDM) as listed in the respective cell line; ^c^ SI = selectivity index; ^d^ Ro5 = rule of five.

## Supplementary Materials

The following are available online, Figure S1: Distribution of the Colombian scientific literature on leishmaniasis, Figure S2: t-SNE plot using MACCS and Morgan fingerprints colored by HCA, Figure S3: Experimental versus predicted activity (pEC_50_) for machine learning models, Table S1: PRISMA checklist, Table S2: List of compounds and their antileishmanial activity retrieved from the reviewed literature, Table S3: Antileishmanial activity predicted by machine learning models.
