# Supplementary material for: Colombian Contributions Fighting Leishmaniasis: A Systematic Review on Antileishmanials Combined with Chemoinformatics Analysis
Source: Molecules. 2020 Dec 3;25(23):5704. doi: 10.3390/molecules25235704 (PMC7730898; doi:10.3390/molecules25235704)
Supplement: Supplementary file 1 [file molecules-25-05704-s001.pdf]

Supplementary Material

Colombian Contributions Fighting Leishmaniasis: A Systematic Review on Antileishmanials Combined with Chemoinformatics Analysis

Jeysson Sánchez-Suárez [1], Freddy A. Bernal [2,†] and Ericsson Coy-Barrera [2,*]

[1] Bioprospecting Research Group, School of Engineering, Universidad de La Sabana, Chía 250001, Colombia
[2] Bioorganic Chemistry Laboratory, Universidad Militar Nueva Granada, Cajicá 250247, Colombia
* Correspondence: ericsson.coy@unimilitar.edu.co
† Current address: Transfer Group Anti-infectives, Leibniz Institute for Natural Product Research and Infection Biology, HKI, Beutenbergstraße 11a, 07745 Jena, Germany

Content

		Page
**Table S1.**	PRISMA 2009 Checklist	2
**Table S2.**	List of compounds and their antileishmanial activity retrieved from the literature reviewed.	4
**Table S3.**	Antileishmanial activity predicted by machine learning models.	51
**Figure S1.**	Distribution of the Colombian scientific literature on leishmaniasis.	63
**Figure S2.**	t-SNE plot using MACCS and Morgan fingerprints colored by HCA.	63
**Figure S3.**	Experimental versus predicted activity (pEC_{50}) for machine learning models.	64

Table S1. PRISMA 2009 Checklist

Section/topic	#	Checklist item	Reported on page #
**TITLE: Colombian Contributions Fighting Leishmaniasis: A Systematic Review on Antileishmanials Combined with Chemoinformatics Analysis**			
Title	1	Identify the report as a systematic review, meta-analysis, or both.	1
**ABSTRACT**			
Structured summary	2	Provide a structured summary including, as applicable: background; objectives; data sources; study eligibility criteria, participants, and interventions; study appraisal and synthesis methods; results; limitations; conclusions and implications of key findings; systematic review registration number.	1
**INTRODUCTION**			
Rationale	3	Describe the rationale for the review in the context of what is already known.	1-2
Objectives	4	Provide an explicit statement of questions being addressed with reference to participants, interventions, comparisons, outcomes, and study design (PICOS).	2
**METHODS**			
Protocol and registration	5	Indicate if a review protocol exists, if and where it can be accessed (e.g., Web address), and, if available, provide registration information including registration number.	N/A
Eligibility criteria	6	Specify study characteristics (e.g., PICOS, length of follow-up) and report characteristics (e.g., years considered, language, publication status) used as criteria for eligibility, giving rationale.	15
Information sources	7	Describe all information sources (e.g., databases with dates of coverage, contact with study authors to identify additional studies) in the search and date last searched.	15
Search	8	Present full electronic search strategy for at least one database, including any limits used, such that it could be repeated.	15
Study selection	9	State the process for selecting studies (i.e., screening, eligibility, included in systematic review, and, if applicable, included in the meta-analysis).	15
Data collection process	10	Describe method of data extraction from reports (e.g., piloted forms, independently, in duplicate) and any processes for obtaining and confirming data from investigators.	15
Data items	11	List and define all variables for which data were sought (e.g., PICOS, funding sources) and any assumptions and simplifications made.	N/A
Risk of bias in individual studies	12	Describe methods used for assessing risk of bias of individual studies (including specification of whether this was done at the study or outcome level), and how this information is to be used in any data synthesis.	N/A
Summary measures	13	State the principal summary measures (e.g., risk ratio, difference in means).	N/A
Synthesis of results	14	Describe the methods of handling data and combining results of studies, if done, including measures of consistency (e.g., I^2) for each meta-analysis.	15-16
Risk of bias across studies	15	Specify any assessment of risk of bias that may affect the cumulative evidence (e.g., publication bias, selective reporting within studies).	N/A
Additional analyses	16	Describe methods of additional analyses (e.g., sensitivity or subgroup analyses, meta-regression), if done, indicating which were pre-specified.	16-17

**RESULTS**			
Study selection	17	Give numbers of studies screened, assessed for eligibility, and included in the review, with reasons for exclusions at each stage, ideally with a flow diagram.	3
Study characteristics	18	For each study, present characteristics for which data were extracted (e.g., study size, PICOS, follow-up period) and provide the citations.	3
Risk of bias within studies	19	Present data on risk of bias of each study and, if available, any outcome level assessment (see item 12).	N/A
Results of individual studies	20	For all outcomes considered (benefits or harms), present, for each study: (a) simple summary data for each intervention group (b) effect estimates and confidence intervals, ideally with a forest plot.	N/A
Synthesis of results	21	Present results of each meta-analysis done, including confidence intervals and measures of consistency.	3-7
Risk of bias across studies	22	Present results of any assessment of risk of bias across studies (see Item 15).	N/A
Additional analysis	23	Give results of additional analyses, if done (e.g., sensitivity or subgroup analyses, meta-regression [see Item 16]).	7-15
**DISCUSSION**			
Summary of evidence	24	Summarize the main findings including the strength of evidence for each main outcome; consider their relevance to key groups (e.g., healthcare providers, users, and policy makers).	3-7
Limitations	25	Discuss limitations at study and outcome level (e.g., risk of bias), and at review-level (e.g., incomplete retrieval of identified research, reporting bias).	3-7
Conclusions	26	Provide a general interpretation of the results in the context of other evidence, and implications for future research.	17-18
**FUNDING**			
Funding	27	Describe sources of funding for the systematic review and other support (e.g., supply of data); role of funders for the systematic review.	18

From: Moher D, Liberati A, Tetzlaff J, Altman DG, The PRISMA Group (2009). Preferred Reporting Items for Systematic Reviews and Meta-Analyses: The PRISMA Statement. PLoS Med 6(7): e1000097. doi:10.1371/journal.pmed1000097

Table S2. List of compounds and their antileishmanial activity retrieved from the literature reviewed.

Comp[a]	Ori[b]	SMILES[c]	*Leishmania* species[d]	Parasite form[e]	Leishmanicidal Potential[f]	EC_{50} (μM)[g]	Reference
**1**	S	CC(OC1=C(N=C(/C=C/C2=CC=CC(OC(C)=O)=C2OC(C)=O)C=C3)C3=CC=C1)=O	*L. panamensis*	IA	ND	13.8	(Sánchez et al., 2014)
**2**	S	O=C(C)OC(C(OC)=C1)=CC=C1/C=C/C2=NC3=CC=CC=C3C=C2	*L. panamensis*	IA	ND	213.4	(Sánchez et al., 2014)
**3**	S	CC(OC1=C(OC(C)=O)C=CC=C1/C=C/C2=NC3=CC=CC=C3C=C2)=O	*L. panamensis*	IA	High	4.03	(Sánchez et al., 2014)
**4**	N	O=C1C2=C(O)C=C(O)C=C2OC(C3=CC=C(OC4=C(O)C=C(O)C5=C4OC(C6=CC=C(O)C=C6)=CC5=O)C=C3)=C1	*L. donovani*	AA	High	7.25	(Weniger et al., 2004)
**5**	S	O=C1NC2=CC=CC=C2C=C1C3NC(C=CC=C4)=C4C(NC3)=O	*L. panamensis*	IA	Low	361.2	(Insuasty et al., 2017)
**6**	S	O=C1NC2=CC=C(C)C=C2C=C1C3NC(C=CC=C4)=C4C(NC3)=O	*L. panamensis*	IA	Low	71.8	(Insuasty et al., 2017)
**7**	S	O=C1NC2=CC=C(Cl)C=C2C=C1C3NC(C=CC=C4)=C4C(NC3)=O	*L. panamensis*	IA	Low	57.3	(Insuasty et al., 2017)
**8**	S	O=C1NC2=CC(Cl)=CC=C2C=C1C3NC(C=CC=C4)=C4C(NC3)=O	*L. panamensis*	IA	Low	229.8	(Insuasty et al., 2017)
**9**	S	O=C1NC2=CC=C(Br)C=C2C=C1C3NC(C=CC=C4)=C4C(NC3)=O	*L. panamensis*	IA	Low	39.8	(Insuasty et al., 2017)
**10**	S	O=C1NC2=C3C(C=CC=C3)=CC=C2C=C1C4NC(C=CC=C5)=C5C(NC4)=O	*L. panamensis*	IA	Intermediate	14.0	(Insuasty et al., 2017)
**11**	S	O=C(NC1)C2=C(C=CC=C2)NC1C3=CC4=CC=CC(C)=C4N=C3OCCCC	*L. panamensis*	IA	High	8.93	(Insuasty et al., 2017)
**12**	S	O=C(NC1)C2=C(C=CC=C2)NC1C3=CC4=CC(Br)=CC=C4N=C3OCCCC	*L. panamensis*	IA	Low	77.9	(Insuasty et al., 2017)
**13**	S	[H]C1=C([H])C=C2C(=C1)[N+]([O-])=C(C)C(C)=[N+]2[O-]	*L. infantum*	AA	ND	100	(Quiliano et al., 2017)
**14**	S	[H]C1=C(OC)C=C2C(=C1)[N+]([O-])=C(C)C(C)=[N+]2[O-]	*L. infantum*	AA	ND	100	(Quiliano et al., 2017)
**15**	S	[H]C1=C(Cl)C=C2C(=C1)[N+]([O-])=C(C)C(C)=[N+]2[O-]	*L. infantum*	AA	ND	101	(Quiliano et al., 2017)
**16**	S	[H]C1=C([H])C=C2C(=C1)[N+]([O-])=C(C)C(=[N+]2[O-])C(F)(F)F	*L. infantum*	AA	Low	30.5	(Quiliano et al., 2017)
**17**	S	[H]C1=C([H])C=C2C(=C1)[N+]([O-])=C(C)C(\C=N\NC1=CC=CC=C1)=[N+]2[O-]	*L. infantum*	AA	ND	100	(Quiliano et al., 2017)
**18**	S	[H]C1=C([H])C=C2C(=C1)[N+]([O-])=C(C)C(\C=N\NC1=CC=CC(Cl)=C1)=[N+]2[O-]	*L. infantum*	AA	ND	100	(Quiliano et al., 2017)
**19**	S	[H]C1=C([H])C=C2C(=C1)[N+]([O-])=C(C)C(\C=N\NC1=CC=C(Cl)C(Cl)=C1)=[N+]2[O-]	*L. infantum*	AA	ND	100	(Quiliano et al., 2017)
**20**	S	[H]C1=C([H])C=C2C(=C1)[N+]([O-])=C(C)C(\C=N\NC1=C(Cl)C=C(Cl)C=C1Cl)=[N+]2[O-]	*L. infantum*	AA	ND	100	(Quiliano et al., 2017)
**21**	S	[H]C1=C([H])C=C2C(=C1)[N+]([O-])=C(C)C(\C=N\NC1=CC=C(C=C1)C(O)=O)=[N+]2[O-]	*L. infantum*	AA	ND	100	(Quiliano et al., 2017)
**22**	S	[H]C1=C([H])C=C2C(=C1)[N+]([O-])=C(C)C(\C=N\NS(=O)(=O)C1=CC=C(C)C=C1)=[N+]2[O-]	*L. infantum*	AA	Low	53.4	(Quiliano et al., 2017)
**23**	S	[H]C1=C([H])C=C2C(=C1)[N+]([O-])=C(C)C(\C=N\NC(=O)C1=CC=CC=C1)=[N+]2[O-]	*L. infantum*	AA	Low	59.1	(Quiliano et al., 2017)
**24**	S	[H]C1=C([H])C=C2C(=C1)[N+]([O-])=C(C)C(\C=N\NC(=O)C1=CC=NC=C1)=[N+]2[O-]	*L. infantum*	AA	ND	100	(Quiliano et al., 2017)

Comp[a]	Ori[b]	SMILES[c]	*Leishmania* species[d]	Parasite form[e]	Leishmanicidal Potential[f]	EC_{50} (μM)[g]	Reference
**25**	S	[H]C1=C([H])C=C2C(=C1)[N+]([O-])=C(C)C(\C=N\NC(=O)C1=CC=C(OC)C=C1)=[N+]2[O-]	*L. infantum*	AA	ND	100	(Quiliano et al., 2017)
**26**	S	[H]C1=C([H])C=C2C(=C1)[N+]([O-])=C(C)C(\C=N\NC(=O)C1=CC=C(Cl)C=C1)=[N+]2[O-]	*L. infantum*	AA	Low	52.3	(Quiliano et al., 2017)
**27**	S	[H]C1=C([H])C=C2C(=C1)[N+]([O-])=C(C)C(\C=N\NC(C)=O)=[N+]2[O-]	*L. infantum*	AA	ND	100	(Quiliano et al., 2017)
**28**	S	[H]C1=C(OC)C=C2C(=C1)[N+]([O-])=C(C)C(\C=N\NC1=C(Cl)C=C(Cl)C=C1Cl)=[N+]2[O-]	*L. infantum*	AA	ND	100	(Quiliano et al., 2017)
**29**	S	[H]C1=C(Cl)C=C2C(=C1)[N+]([O-])=C(C)C(\C=N\NC1=C(Cl)C=C(Cl)C=C1Cl)=[N+]2[O-]	*L. infantum*	AA	ND	100	(Quiliano et al., 2017)
**30**	S	[H]C1=C(OC)C=C2C(=C1)[N+]([O-])=C(C)C(\C=N\NC1=CC=NC3=C1C=CC(Cl)=C3)=[N+]2[O-]	*L. infantum*	AA	ND	100	(Quiliano et al., 2017)
**31**	S	[H]C1=C(Cl)C=C2C(=C1)[N+]([O-])=C(C)C(\C=N\NC1=CC=NC3=C1C=CC(Cl)=C3)=[N+]2[O-]	*L. infantum*	AA	ND	100	(Quiliano et al., 2017)
**32**	S	O=C(C1=CC=C(Cl)C=C1)/C=C/C2=CC=C(OC3=NC4=C(C(N(C)C(N4C)=O)=O)N3C)C(OC)=C2	*L. panamensis*	IA	Low	26.868	(Insuasty et al., 2015)
**33**	S	O=C(C1=CC=C(C)C=C1)/C=C/C2=CC=C(OC3=NC4=C(C(N(C)C(N4C)=O)=O)N3C)C(OC)=C2	*L. panamensis*	IA	High	5.65	(Insuasty et al., 2015)
**34**	S	O=C(C1=CC=C(OC)C=C1)/C=C/C2=CC=C(OC3=NC4=C(C(N(C)C(N4C)=O)=O)N3C)C(OC)=C2	*L. panamensis*	IA	Low	29.821	(Insuasty et al., 2015)
**35**	S	CN1C(OC2=C(OC)C=C(C3CC(C4=CC=C(Br)C=C4)=NN3)C=C2)=NC5=C1C(N(C)C(N5C)=O)=O	*L. panamensis*	IA	Low	36.239	(Insuasty et al., 2015)
**36**	S	CN1C(OC2=C(OC)C=C(C3CC(C4=CC(OC)=C(OC)C(OC)=C4)=NN3)C=C2)=NC5=C1C(N(C)C(N5C)=O)=O	*L. panamensis*	IA	Low	28.716	(Insuasty et al., 2015)
**37**	S	CN1C(OC2=C(OC)C=C(C3CC(C4=CC=C(Br)C=C4)=NN3C(C)=O)C=C2)=NC5=C1C(N(C)C(N5C)=O)=O	*L. panamensis*	IA	Intermediate	24.651	(Insuasty et al., 2015)
**38**	S	CN1C(OC2=C(OC)C=C(C3CC(C4=CC=C(C)C=C4)=NN3C(C)=O)C=C2)=NC5=C1C(N(C)C(N5C)=O)=O	*L. panamensis*	IA	Low	35.063	(Insuasty et al., 2015)
**39**	S	CN1C(OC2=C(OC)C=C(C3CC(C4=CC=C(OC)C=C4)=NN3C(C)=O)C=C2)=NC5=C1C(N(C)C(N5C)=O)=O	*L. panamensis*	IA	Low	27.433	(Insuasty et al., 2015)
**40**	S	CN1C(OC2=C(OC)C=C(C3CC(C4=CC(OC)=C(OC)C(OC)=C4)=NN3C(C)=O)C=C2)=NC5=C1C(N(C)C(N5C)=O)=O	*L. panamensis*	IA	Intermediate	21.276	(Insuasty et al., 2015)
**41**	S	CN1C(OC2=C(OC)C=C(C3CC(C4=CC=C(Br)C=C4)=NN3C=O)C=C2)=NC5=C1C(N(C)C(N5C)=O)=O	*L. panamensis*	IA	Low	28.441	(Insuasty et al., 2015)
**42**	S	O=C(C1=CC=CC=C1)/C=C/C2=CC=C(OC3=NC4=C(C(N(C)C(N4C)=O)=O)N3C)C(OC)=C2	*L. panamensis*	IA	N/A		(Insuasty et al., 2015)
**43**	S	O=C(C1=CC=C(Br)C=C1)/C=C/C2=CC=C(OC3=NC4=C(C(N(C)C(N4C)=O)=O)N3C)C(OC)=C2	*L. panamensis*	IA	N/A		(Insuasty et al., 2015)

Comp[a]	Ori[b]	SMILES[c]	*Leishmania* species[d]	Parasite form[e]	Leishmanicidal Potential[f]	EC_{50} (μM)[g]	Reference
**44**	S	O=C(C1=CC(OC)=C(OC)C(OC)=C1)/C=C/C2=CC=C(OC3=NC4=C(C(N(C)C(N4C)=O)=O)N3C)C(OC)=C2	*L. panamensis*	IA	High	4.849	(Insuasty et al., 2015)
**45**	S	CN1C(OC2=C(OC)C=C(C3CC(C4=CC=CC=C4)=NN3)C=C2)=NC5=C1C(N(C)C(N5C)=O)=O	*L. panamensis*	IA	N/A		(Insuasty et al., 2015)
**46**	S	CN1C(OC2=C(OC)C=C(C3CC(C4=CC=C(Cl)C=C4)=NN3)C=C2)=NC5=C1C(N(C)C(N5C)=O)=O	*L. panamensis*	IA	N/A		(Insuasty et al., 2015)
**47**	S	CN1C(OC2=C(OC)C=C(C3CC(C4=CC=C(C)C=C4)=NN3)C=C2)=NC5=C1C(N(C)C(N5C)=O)=O	*L. panamensis*	IA	N/A		(Insuasty et al., 2015)
**48**	S	CN1C(OC2=C(OC)C=C(C3CC(C4=CC=C(OC)C=C4)=NN3)C=C2)=NC5=C1C(N(C)C(N5C)=O)=O	*L. panamensis*	IA	N/A		(Insuasty et al., 2015)
**49**	S	CN1C(OC2=C(OC)C=C(C3CC(C4=CC=CC=C4)=NN3C(C)=O)C=C2)=NC5=C1C(N(C)C(N5C)=O)=O	*L. panamensis*	IA	N/A		(Insuasty et al., 2015)
**50**	S	CN1C(OC2=C(OC)C=C(C3CC(C4=CC=C(Cl)C=C4)=NN3C(C)=O)C=C2)=NC5=C1C(N(C)C(N5C)=O)=O	*L. panamensis*	IA	N/A		(Insuasty et al., 2015)
**51**	S	CN1C(OC2=C(OC)C=C(C3CC(C4=CC=CC=C4)=NN3C=O)C=C2)=NC5=C1C(N(C)C(N5C)=O)=O	*L. panamensis*	IA	N/A		(Insuasty et al., 2015)
**52**	S	CN1C(OC2=C(OC)C=C(C3CC(C4=CC=C(Cl)C=C4)=NN3C=O)C=C2)=NC5=C1C(N(C)C(N5C)=O)=O	*L. panamensis*	IA	N/A		(Insuasty et al., 2015)
**53**	S	CN1C(OC2=C(OC)C=C(C3CC(C4=CC=C(C)C=C4)=NN3C=O)C=C2)=NC5=C1C(N(C)C(N5C)=O)=O	*L. panamensis*	IA	N/A		(Insuasty et al., 2015)
**54**	S	CN1C(OC2=C(OC)C=C(C3CC(C4=CC=C(OC)C=C4)=NN3C=O)C=C2)=NC5=C1C(N(C)C(N5C)=O)=O	*L. panamensis*	IA	N/A		(Murillo et al., 2019)
**55**	S	CN1C(OC2=C(OC)C=C(C3CC(C4=CC(OC)=C(OC)C(OC)=C4)=NN3C=O)C=C2)=NC5=C1C(N(C)C(N5C)=O)=O	*L. panamensis*	IA	N/A		(Peniche et al., 2015)
**56**	S	CN1C(OC2=CC=C(C3NC(N(C4=CC=CC=C4)N=C5C)=C5N=C(C6=CC=CC=C6)C3)C=C2OC)=NC7=C1C(N(C)C(N7C)=O)=O	*L. panamensis*	IA	N/A		(Osorio et al., 2006)
**57**	S	CN1C(OC2=CC=C(C3NC(N(C4=CC=CC=C4)N=C5C)=C5N=C(C6=CC=C(Cl)C=C6)C3)C=C2OC)=NC7=C1C(N(C)C(N7C)=O)=O	*L. panamensis*	IA	N/A		(López et al., 2009)
**58**	S	CN1C(OC2=CC=C(C3NC(N(C4=CC=CC=C4)N=C5C)=C5N=C(C6=CC=C(F)C=C6)C3)C=C2OC)=NC7=C1C(N(C)C(N7C)=O)=O	*L. panamensis*	IA	N/A		(Insuasty et al., 2015)
**59**	S	CN1C(OC2=CC=C(C3NC(N(C4=CC=CC=C4)N=C5C)=C5N=C(C6=CC=C(C)C=C6)C3)C=C2OC)=NC7=C1C(N(C)C(N7C)=O)=O	*L. panamensis*	IA	N/A		(Insuasty et al., 2015)
**60**	S	CN1C(OC2=CC=C(C3NC(N(C4=CC=CC=C4)N=C5C)=C5N=C(C6=CC=C(OC)C=C6)C3)C=C2OC)=NC7=C1C(N(C)C(N7C)=O)=O	*L. panamensis*	IA	N/A		(Insuasty et al., 2015)
**61**	S	CN1C(OC2=CC=C(C3NC(N(C4=CC=CC=C4)N=C5C)=C5N=C(C6=CC(OC)=C(OC)C(OC)=C6)C3)C=C2OC)=NC7=C1C(N(C)C(N7C)=O)=O	*L. panamensis*	IA	N/A		(Insuasty et al., 2015)
**62**	S	[H]C1=C(Cl)C=C2C(=C1)[N+]([O-])=C(NS(=O)(=O)C1=CC=C3C=CC=CC3=C1)C(C#N)=[N+]2[O-]	*L. amazonensis*	AA	Intermediate	20	(Barea et al., 2011)

Comp[a]	Ori[b]	SMILES[c]	*Leishmania* species[d]	Parasite form[e]	Leishmanicidal Potential[f]	EC_{50} (μM)[g]	Reference
**63**	S	[H]C1=C(C)C=C2C(=C1)[N+]([O-])=C(NS(=O)(=O)C1=CC=C3C=CC=CC3=C1)C(C#N)=[N+]2[O-]	*L. amazonensis*	AA	Intermediate	16.3	(Barea et al., 2011)
**64**	S	CC1=C(C)C=C2C(=C1)[N+]([O-])=C(NS(=O)(=O)C1=CC=C3C=CC=CC3=C1)C(C#N)=[N+]2[O-]	*L. amazonensis*	AA	ND	100	(Barea et al., 2011)
**65**	S	[H]C1=C(C=C2C([N+]([O-])=C(C(C#N)=[N+]2[O-])NS(=O)(C3=C([N+]([O-])=O)C=CC=C3)=O)=C1)Cl	*L. amazonensis*	AA	High	3.1	(Barea et al., 2011)
**66**	S	[H]C1=C(C=C2C([N+]([O-])=C(C(C#N)=[N+]2[O-])NS(=O)(C3=CC=C([N+]([O-])=O)C=C3)=O)=C1)Cl	*L. amazonensis*	AA	High	2.1	(Barea et al., 2011)
**67**	S	[O-][N+](C1=CC=C(S(=O)(NC2=[N+](C3=CC(Cl)=C(C=C3[N+]([O-])=C2C#N)Cl)[O-])=O)C=C1)=O	*L. amazonensis*	AA	Intermediate	15.9	(Barea et al., 2011)
**68**	S	[H]C1=C(C=C2C([N+]([O-])=C(C(C#N)=[N+]2[O-])NS(=O)(C3=CC=C([N+]([O-])=O)C=C3)=O)=C1)[H]	*L. amazonensis*	AA	Low	86.3	(Barea et al., 2011)
**69**	S	[H]C1=C([H])C=C2C(=C1)[N+]([O-])=C(NC(=O)C1=CC=CC=C1OC(C)=O)C(C#N)=[N+]2[O-]	*L. amazonensis*	AA	Low	111.8	(Barea et al., 2011)
**70**	S	[H]C1=C(Cl)C=C2C(=C1)[N+]([O-])=C(NC(=O)C1=CC=CC=C1OC(C)=O)C(C#N)=[N+]2[O-]	*L. amazonensis*	AA	Low	33.6	(Barea et al., 2011)
**71**	S	[H]C1=C(C)C=C2C(=C1)[N+]([O-])=C(NC(=O)C1=CC=CC=C1OC(C)=O)C(C#N)=[N+]2[O-]	*L. amazonensis*	AA	Intermediate	18.8	(Barea et al., 2011)
**72**	S	[H]C1=C(OC)C=C2C(=C1)[N+]([O-])=C(NC(=O)C1=CC=CC=C1OC(C)=O)C(C#N)=[N+]2[O-]	*L. amazonensis*	AA	Low	42.4	(Barea et al., 2011)
**73**	S	CC(=O)OC1=CC=CC=C1C(=O)NC1=[N+]([O-])C2=CC(F)=C(F)C=C2[N+]([O-])=C1C#N	*L. amazonensis*	AA	Intermediate	14.8	(Barea et al., 2011)
**74**	S	CC(=O)OC1=CC=CC=C1C(=O)NC1=[N+]([O-])C2=CC(C)=C(C)C=C2[N+]([O-])=C1C#N	*L. amazonensis*	AA	Intermediate	17.6	(Barea et al., 2011)
**75**	S	[H]C1=C(F)C=C2C(=C1)[N+]([O-])=C(NC(=O)C1=CC=CC=C1OC(C)=O)C(C#N)=[N+]2[O-]	*L. amazonensis*	AA	High	7.3	(Barea et al., 2011)
**76**	S	C[C@@]1(CCC[C@]2(CO)C)[C@]2([H])CC[C@@]3(C=C4)[C@@]1([H])CC[C@@]4(C)C3	*L. braziliensis*	IA	Intermediate	15.959	(Murillo et al., 2019)
**77**	S	C[C@@]1(CCC[C@]2(C)CO)[C@]2([H])CC[C@@]3(C=C4)[C@@]1([H])CC[C@@]4(C)C3	*L. braziliensis*	IA	Intermediate	18.387	(Murillo et al., 2019)
**78**	S	C[C@@]1(CCC[C@]2(CO)C)[C@]2([H])CC[C@@]3(CC4)[C@@]1([H])CC[C@@]4(C)C3	*L. braziliensis*	IA	Intermediate	18.259	(Murillo et al., 2019)
**79**	S	C[C@@]1(CCC[C@]2(C(O)=O)C)[C@]2([H])CC[C@@]3(C=C4)[C@@]1([H])CC[C@@]4(C)C3	*L. braziliensis*	IA	ND	66.176	(Murillo et al., 2019)
**80**	S	C[C@@]1(CCC[C@]2(C(O)=O)C)[C@]2([H])CC[C@@]3([C@@H]4[C@H]5O4)[C@@]1([H])CC[C@@]5(C)C3	*L. braziliensis*	IA	Low	43.366	(Murillo et al., 2019)
**81**	S	C[C@@]1(CCC[C@]2(CO)C)[C@]2([H])CC[C@@]3([C@@H]4[C@H]5O4)[C@@]1([H])CC[C@@]5(C)C3	*L. braziliensis*	IA	ND	63.108	(Murillo et al., 2019)

Comp[a]	Ori[b]	SMILES[c]	*Leishmania* species[d]	Parasite form[e]	Leishmanicidal Potential[f]	EC_{50} (μM)[g]	Reference
**82**	S	C[C@@]1(CCC[C@]2(C(O)=O)C)[C@]2([H])CC[C@@]3(CC4=O)[C@@]1([H])CC[C@@]4(C)C3	*L. braziliensis*	IA	ND	20.112	(Murillo et al., 2019)
**83**	S	C[C@@]1(CCC[C@]2(C(OCC)=O)C)[C@]2([H])CC[C@@]3(CC4=O)[C@@]1([H])CC[C@@]4(C)C3	*L. braziliensis*	IA	Low	60.361	(Murillo et al., 2019)
**84**	S	C[C@@]1(CCC[C@]2(C(O)=O)C)[C@]2([H])CC[C@@]3(C/C4=N/NS(C5=CC=C(C)C=C5)(=O)=O)[C@@]1([H])CC[C@@]4(C)C3	*L. braziliensis*	IA	High	2.262	(Murillo et al., 2019)
**85**	S	C[C@@]1(CCC[C@]2(C(OCC)=O)C)[C@]2([H])CC[C@@]3(C/C4=N/NS(C5=CC=C(C)C=C5)(=O)=O)[C@@]1([H])CC[C@@]4(C)C3	*L. braziliensis*	IA	High	2.528	(Murillo et al., 2019)
**86**	S	C[C@@]1(CCC[C@]2(C(OCC)=O)C)[C@]2([H])CC[C@@]3(C/C4=N/O)[C@@]1([H])CC[C@@]4(C)C3	*L. braziliensis*	IA	High	8.581	(Murillo et al., 2019)
**87**	S	C[C@@]1(CCC[C@]2(C(OCC)=O)C)[C@]2([H])CC[C@@]3(CC#N)[C@@]1([H])CC=C(C)C3	*L. braziliensis*	IA	Intermediate	14.858	(Murillo et al., 2019)
**88**	S	C[C@@]1(CCC[C@]2(C(OCC)=O)C)[C@]2([H])CC[C@@]3(C[C@H]4O)[C@@]1([H])CC[C@@]4(C)C3	*L. braziliensis*	IA	Intermediate	20.1	(Murillo et al., 2019)
**89**	S	S=C(SSC(N(CC)CC)=S)N(CC)CC	*L. major*	IA	High	0.044	(Peniche et al., 2015)
**89**	S	S=C(SSC(N(CC)CC)=S)N(CC)CC	*L. donovani*	IA	High	0.023	(Peniche et al., 2015)
**90**	S	S=C(SSC(N1CCCCC1)=S)N2CCCCC2	*L. major*	IA	High	0.058	(Peniche et al., 2015)
**90**	S	S=C(SSC(N1CCCCC1)=S)N2CCCCC2	*L. donovani*	IA	High	0.062	(Peniche et al., 2015)
**91**	S	S=C(SSC(N(C)C)=S)N(C)C	*L. major*	IA	High	0.035	(Peniche et al., 2015)
**91**	S	S=C(SSC(N(C)C)=S)N(C)C	*L. donovani*	IA	High	2.931	(Peniche et al., 2015)
**92**	S	S=C(SSC(N(C(C)C)C(C)C)=S)N(C(C)C)C(C)C	*L. major*	IA	High	0.636	(Peniche et al., 2015)
**92**	S	S=C(SSC(N(C(C)C)C(C)C)=S)N(C(C)C)C(C)C	*L. donovani*	IA	Intermediate	17.35	(Peniche et al., 2015)
**93**	S	S=C(SSC(N1CCOCC1)=S)N2CCOCC2	*L. major*	IA	High	0.03	(Peniche et al., 2015)
**93**	S	S=C(SSC(N1CCOCC1)=S)N2CCOCC2	*L. donovani*	IA	High	0.618	(Peniche et al., 2015)
**94**	S	S=C(SSC(N(CCCC)CCCC)=S)N(CCCC)CCCC	*L. major*	IA	High	2.879	(Peniche et al., 2015)
**94**	S	S=C(SSC(N(CCCC)CCCC)=S)N(CCCC)CCCC	*L. donovani*	IA	Intermediate	18.44	(Peniche et al., 2015)
**95**	S	S=C(SSC(N1CCN(C)CC1)=S)N2CCN(C)CC2	*L. major*	IA	High	0.278	(Peniche et al., 2015)
**95**	S	S=C(SSC(N1CCN(C)CC1)=S)N2CCN(C)CC2	*L. donovani*	IA	High	0.39	(Peniche et al., 2015)
**96**	S	S=C(SSC(N(CC(C)C)CC1=CC=CS1)=S)N(CC(C)C)CC2=CC=CS2	*L. major*	IA	High	1.699	(Peniche et al., 2015)
**96**	S	S=C(SSC(N(CC(C)C)CC1=CC=CS1)=S)N(CC(C)C)CC2=CC=CS2	*L. donovani*	IA	Intermediate	16.85	(Peniche et al., 2015)
**97**	S	S=C(SSC(N(C)C1CCS(C1)(=O)=O)=S)N(C)C2CCS(C2)(=O)=O	*L. major*	IA	High	1.49	(Peniche et al., 2015)
**97**	S	S=C(SSC(N(C)C1CCS(C1)(=O)=O)=S)N(C)C2CCS(C2)(=O)=O	*L. donovani*	IA	High	9.06	(Peniche et al., 2015)
**98**	S	ClCCN(CCCl)C1=NC(Cl)=C(S1)C=O	*L. panamensis*	IA	Low	53.37	(Cuartas et al., 2020)
**99**	S	ClCCN(CCCl)C1=NC(Cl)=C(S1)\C=C\C(=O)C1=CC=C(Cl)C=C1	*L. panamensis*	IA	ND	4	(Cuartas et al., 2020)
**100**	S	ClCCN(CCCl)C1=NC(Cl)=C(S1)\C=C\C(=O)C1=CC=C(Br)C=C1	*L. panamensis*	IA	ND	7	(Cuartas et al., 2020)
**101**	S	FC1=CC=C(C=C1)C(=O)\C=C\C1=C(Cl)N=C(S1)N(CCCl)CCCl	*L. panamensis*	IA	ND	13	(Cuartas et al., 2020)
**102**	S	ClCCN(CCCl)C1=NC(Cl)=C(S1)\C=C\C(=O)C1=CC2=C(OCO2)C=C1	*L. panamensis*	IA	ND	30	(Cuartas et al., 2020)
**103**	S	COC1=CC=C(C=C1)C(=O)\C=C\C1=C(Cl)N=C(S1)N(CCCl)CCCl	*L. panamensis*	IA	ND	5	(Cuartas et al., 2020)

Comp[a]	Ori[b]	SMILES[c]	*Leishmania* species[d]	Parasite form[e]	Leishmanicidal Potential[f]	EC_{50} (μM)[g]	Reference
**104**	S	COC1=CC(=CC(OC)=C1OC)C(=O)\C=C\C1=C(Cl)N=C(S1)N(CCCl)CCCl	*L. panamensis*	IA	ND	10	(Cuartas et al., 2020)
**105**	S	ClCCN(CCCl)C1=NC(Cl)=C(S1)\C=C\C(=O)C1=CC=CC=C1	*L. panamensis*	IA	ND	4	(Cuartas et al., 2020)
**106**	S	CC(=O)N1N=C(CC1C1=C(Cl)N=C(S1)N(CCCl)CCCl)C1=CC=C(Cl)C=C1	*L. panamensis*	IA	ND	4	(Cuartas et al., 2020)
**107**	S	CC(=O)N1N=C(CC1C1=C(Cl)N=C(S1)N(CCCl)CCCl)C1=CC=C(Br)C=C1	*L. panamensis*	IA	ND	7	(Cuartas et al., 2020)
**108**	S	CC(=O)N1N=C(CC1C1=C(Cl)N=C(S1)N(CCCl)CCCl)C1=CC=C(F)C=C1	*L. panamensis*	IA	ND	3	(Cuartas et al., 2020)
**109**	S	CC(=O)N1N=C(CC1C1=C(Cl)N=C(S1)N(CCCl)CCCl)C1=CC=C2OCOC2=C1	*L. panamensis*	IA	ND	7	(Cuartas et al., 2020)
**110**	S	ClCCN(CCCl)C1=NC(Cl)=C(S1)C1CC(=NN1C1=CC=CC=C1)C1=CC=C(Cl)C=C1	*L. panamensis*	IA	Low	31.49	(Cuartas et al., 2020)
**111**	S	ClCCN(CCCl)C1=NC(Cl)=C(S1)C1CC(=NN1C1=CC=CC=C1)C1=CC=C(Br)C=C1	*L. panamensis*	IA	Intermediate	10.62	(Cuartas et al., 2020)
**112**	S	FC1=CC=C(C=C1)C1=NN(C(C1)C1=C(Cl)N=C(S1)N(CCCl)CCCl)C1=CC=CC=C1	*L. panamensis*	IA	Intermediate	16.95	(Cuartas et al., 2020)
**113**	S	ClCCN(CCCl)C1=NC(Cl)=C(S1)C1CC(=NN1C1=CC=CC=C1)C1=CC2=C(OCO2)C=C1	*L. panamensis*	IA	Intermediate	15.76	(Cuartas et al., 2020)
**114**	S	COC1=CC=C(C=C1)C1=NN(C(C1)C1=C(Cl)N=C(S1)N(CCCl)CCCl)C1=CC=CC=C1	*L. panamensis*	IA	Intermediate	20.2	(Cuartas et al., 2020)
**115**	S	COC1=CC(=CC(OC)=C1OC)C1=NN(C(C1)C1=C(Cl)N=C(S1)N(CCCl)CCCl)C1=CC=CC=C1	*L. panamensis*	IA	High	6.46	(Cuartas et al., 2020)
**116**	S	ClCCN(CCCl)C1=NC(Cl)=C(S1)C1CC(=NN1C1=CC=CC=C1)C1=CC=CC=C1	*L. panamensis*	IA	Intermediate	10.14	(Cuartas et al., 2020)
**117**	S	ClCCN(CCCl)C1=NC(Cl)=C(S1)C1CC(=NN1C1=CC(Cl)=CC(Cl)=C1)C1=CC=C(Cl)C=C1	*L. panamensis*	IA	Low	41.81	(Cuartas et al., 2020)
**118**	S	ClCCN(CCCl)C1=NC(Cl)=C(S1)C1CC(=NN1C1=CC(Cl)=CC(Cl)=C1)C1=CC=C(Br)C=C1	*L. panamensis*	IA	Intermediate	17.78	(Cuartas et al., 2020)
**119**	S	FC1=CC=C(C=C1)C1=NN(C(C1)C1=C(Cl)N=C(S1)N(CCCl)CCCl)C1=CC(Cl)=CC(Cl)=C1	*L. panamensis*	IA	Intermediate	20.09	(Cuartas et al., 2020)
**120**	S	ClCCN(CCCl)C1=NC(Cl)=C(S1)C1CC(=NN1C1=CC(Cl)=CC(Cl)=C1)C1=CC2=C(OCO2)C=C1	*L. panamensis*	IA	Intermediate	10.56	(Cuartas et al., 2020)
**121**	S	COC1=CC=C(C=C1)C1=NN(C(C1)C1=C(Cl)N=C(S1)N(CCCl)CCCl)C1=CC(Cl)=CC(Cl)=C1	*L. panamensis*	IA	Intermediate	14.42	(Cuartas et al., 2020)
**122**	S	COC1=CC(=CC(OC)=C1OC)C1=NN(C(C1)C1=C(Cl)N=C(S1)N(CCCl)CCCl)C1=CC(Cl)=CC(Cl)=C1	*L. panamensis*	IA	Low	41.64	(Cuartas et al., 2020)
**123**	S	ClCCN(CCCl)C1=NC(Cl)=C(S1)C1CC(=NN1C1=CC(Cl)=CC(Cl)=C1)C1=CC=CC=C1	*L. panamensis*	IA	Low	54.14	(Cuartas et al., 2020)

Comp[a]	Ori[b]	SMILES[c]	*Leishmania* species[d]	Parasite form[e]	Leishmanicidal Potential[f]	EC_{50} (μM)[g]	Reference
**124**	N	COC1=CC2=C(C(O)=C1)C(=O)C1=CC=CC=C1N2C	*L. panamensis*	IA	N/A		(Marin et al., 2016)
**125**	S	CC1=CC(O)=C(C(C)C)C=C1	*L. panamensis*	IA	ND	2664.811	(Osorio et al., 2006)
**125**	S	CC1=CC(O)=C(C(C)C)C=C1	*L. panamensis*	IA	ND	2664.811	(Robledo et al., 2005)
**125**	S	CC1=CC(O)=C(C(C)C)C=C1	*L. panamensis*	P	Low	1294.432	(Robledo et al., 2005)
**126**	S	CC1=C([N+]([O-])=O)C(O)=C(C(C)C)C=C1[N+]([O-])=O	*L. panamensis*	IA	Low	244.924	(Osorio et al., 2006)
**126**	S	CC1=C([N+]([O-])=O)C(O)=C(C(C)C)C=C1[N+]([O-])=O	*L. panamensis*	IA	Low	244.924	(Robledo et al., 2005)
**126**	S	CC1=C([N+]([O-])=O)C(O)=C(C(C)C)C=C1[N+]([O-])=O	*L. panamensis*	P	High	1.666	(Robledo et al., 2005)
**127**	S	CC1=C([N+]([O-])=O)C(OC)=C(C(C)C)C=C1[N+]([O-])=O	*L. panamensis*	IA	Low	693.848	(Osorio et al., 2006)
**127**	S	CC1=C([N+]([O-])=O)C(OC)=C(C(C)C)C=C1[N+]([O-])=O	*L. panamensis*	IA	Low	693.848	(Robledo et al., 2005)
**127**	S	CC1=C([N+]([O-])=O)C(OC)=C(C(C)C)C=C1[N+]([O-])=O	*L. panamensis*	P	High	1.22	(Robledo et al., 2005)
**128**	S	CC1=C([N+]([O-])=O)C(OC)=C(C(C)C)C=C1N	*L. panamensis*	IA	Low	1418.907	(Osorio et al., 2006)
**128**	S	CC1=C([N+]([O-])=O)C(OC)=C(C(C)C)C=C1N	*L. panamensis*	IA	Low	1418.907	(Robledo et al., 2005)
**128**	S	CC1=C([N+]([O-])=O)C(OC)=C(C(C)C)C=C1N	*L. panamensis*	P	Low	76.3	(Robledo et al., 2005)
**129**	S	CC1=C([N+]([O-])=O)C(OC)=C(C(C)C)C=C1NN(O)C2=CC(C(C)C)=C(OC)C([N+]([O-])=O)=C2C	*L. panamensis*	IA	Low	454.338	(Osorio et al., 2006)
**130**	S	CC1=C(N)C(OC)=C(C(C)C)C=C1N	*L. panamensis*	IA	Low	137.528	(Osorio et al., 2006)
**130**	S	CC1=C(N)C(OC)=C(C(C)C)C=C1N	*L. panamensis*	IA	Low	137.528	(Robledo et al., 2005)
**130**	S	CC1=C(N)C(OC)=C(C(C)C)C=C1N	*L. panamensis*	P	Low	42.237	(Robledo et al., 2005)
**131**	S	CC1=C([N+]([O-])=O)C(OC)=C(C(C)C)C=C1O	*L. panamensis*	IA	Low	192.803	(Osorio et al., 2006)
**131**	S	CC1=C([N+]([O-])=O)C(OC)=C(C(C)C)C=C1O	*L. panamensis*	IA	Low	192.803	(Robledo et al., 2005)
**131**	S	CC1=C([N+]([O-])=O)C(OC)=C(C(C)C)C=C1O	*L. panamensis*	P	ND	444.247	(Robledo et al., 2005)
**132**	S	CC1=C([N+]([O-])=O)C(OC)=C(C(C)C)C=C1OC	*L. panamensis*	IA	Low	549.943	(Osorio et al., 2006)
**132**	S	CC1=C([N+]([O-])=O)C(OC)=C(C(C)C)C=C1OC	*L. panamensis*	IA	Low	549.943	(Robledo et al., 2005)
**132**	S	CC1=C([N+]([O-])=O)C(OC)=C(C(C)C)C=C1OC	*L. panamensis*	P	ND	418.207	(Robledo et al., 2005)
**133**	S	CC1=C(N)C(OC)=C(C(C)C)C=C1OC	*L. panamensis*	IA	Low	65.028	(Osorio et al., 2006)
**133**	S	CC1=C(N)C(OC)=C(C(C)C)C=C1OC	*L. panamensis*	IA	Low	65.028	(Robledo et al., 2005)
**133**	S	CC1=C(N)C(OC)=C(C(C)C)C=C1OC	*L. panamensis*	P	ND	478.145	(Robledo et al., 2005)
**134**	N	OC1=C2C(OC(C3=CC=C(OC)C(O)=C3)CC2=O)=CC(OC)=C1	*L. panamensis*	IA	Low	53.781	(Robledo et al., 2015)
**135**	S	[H][C@@]12COC3=C(C=C(OC)C(OC)=C3)[C@]1([H])C(=O)C1=C(O2)C2=C(O[C@H](C2)C(C)=C)C=C1	*L. panamensis*	IA	Low	127.2	(Upegui et al., 2014)
**136**	S	[H][C@@]12COC3=C(C=C(OC)C(OC)=C3)[C@]1([H])C(=O)C1=C(O2)C2=C(O[C@H](C2)C2(C)CO2)C=C1	*L. panamensis*	IA	ND	126	(Upegui et al., 2014)
**137**	S	[H][C@@]12COC3=C(C=C(OC)C(OC)=C3)[C@]1([H])C(O)C1=C(O2)C2=C(O[C@H](C2)C(C)=C)C=C1	*L. panamensis*	IA	ND	130	(Upegui et al., 2014)
**138**	S	[H][C@@]12COC3=C(C=C(OC)C(OC)=C3)[C@]1([H])C(=O)C1=C(O2)C2=C(O[C@H](C2)C(C)C)C=C1	*L. panamensis*	IA	ND	126	(Upegui et al., 2014)

Comp[a]	Ori[b]	SMILES[c]	*Leishmania* species[d]	Parasite form[e]	Leishmanicidal Potential[f]	EC_{50} (μM)[g]	Reference
**139**	S	[H][C@@]12COC3=C(C=C(OC)C(OC)=C3)[C@]1([H])C(O)C1=C(O2)C2=C(O[C@H](C2)C(C)C)C=C1	*L. panamensis*	IA	ND	125	(Upegui et al., 2014)
**140**	S	[H][C@@]12COC3=C(C=C(OC)C(OC)=C3)[C@]1([H])CC1=C(O2)C2=C(O[C@H](C2)C(C)=C)C=C1	*L. panamensis*	IA	Intermediate	12.7	(Upegui et al., 2014)
**141**	S	[H][C@@]12COC3=C(C=C(OC)C(OC)=C3)C1([H])CC1=C(O2)C2=C(O[C@H](C2)C(C)C)C=C1	*L. panamensis*	IA	ND	119	(Upegui et al., 2014)
**142**	N	[H][C@@]12C[C@@H](O)C3[C@@]([H])(C(CC(C(C)(C)O)C33C(O)CC(=O)O[C@H]3O)OC(C)=O)C11OC1C(=O)O[C@H]2C1=COC=C1	*L. panamensis*	IA	Low	59	(Granados-Falla et al., 2016)
**142**	N	[H][C@@]12C[C@@H](O)C3[C@@]([H])(C(CC(C(C)(C)O)C33C(O)CC(=O)O[C@H]3O)OC(C)=O)C11OC1C(=O)O[C@H]2C1=COC=C1	*L. panamensis*	IA	Intermediate	16.226	(Coy Barrera et al., 2011)
**143**	N	CN1C(OC2=CC=CC=C2)=CC(=O)C2=CC=CC=C12	*L. panamensis*	IA	ND	119.477	(Coy Barrera et al., 2011)
**144**	N	[H]C1C[C@@]2([H])[C@@H](OC(=O)C3OC23[C@@]2([H])C1C1(C([H])OC(=O)CC1O)C(CC2=O)C(C)(C)O)C1=COC=C1	*L. panamensis*	IA	ND	65.193	(Coy Barrera et al., 2011)
**145**	N	COC1=C2N(C)C(=O)C3=C(OC(C)(C)C=C3)C2=CC=C1	*L. panamensis*	IA	ND	110.652	(Coy Barrera et al., 2011)
**146**	N	COC1=C(OC)C=C2C(OC)=C3C=COC3=NC2=C1	*L. panamensis*	IA	ND	135.091	(Coy Barrera et al., 2011)
**147**	N	COC1=C2C=COC2=NC2=CC=CC=C12	*L. panamensis*	IA	ND	376.765	(Coy Barrera et al., 2011)
**148**	S	O=C(C1=CC=CC=C1)NCCC2=CC(I)=C(O)C(I)=C2	*L. panamensis*	IA	Low	93.994	(Restrepo et al., 2019)
**149**	S	O=C(C1=CC=C(C)C=C1)NCCC2=CC(I)=C(O)C(I)=C2	*L. panamensis*	IA	Low	35.311	(Restrepo et al., 2019)
**150**	S	O=C(C1=CC=C(OC)C=C1)NCCC2=CC(I)=C(O)C(I)=C2	*L. panamensis*	IA	Low	98.563	(Restrepo et al., 2019)
**151**	S	O=C(C1=CC=C([N+]([O-])=O)C=C1)NCCC2=CC(I)=C(O)C(I)=C2	*L. panamensis*	IA	ND	92.956	(Restrepo et al., 2019)
**152**	S	O=C(C1=CC=CC=C1)NCCC2=CC(I)=C(OC)C(I)=C2	*L. panamensis*	IA	Low	66.618	(Restrepo et al., 2019)
**153**	S	O=C(C1=CC=C(C)C=C1)NCCC2=CC(I)=C(OC)C(I)=C2	*L. panamensis*	IA	Low	73.349	(Restrepo et al., 2019)
**154**	S	O=C(C1=CC=C(OC)C=C1)NCCC2=CC(I)=C(OC)C(I)=C2	*L. panamensis*	IA	Low	32.593	(Restrepo et al., 2019)
**155**	S	O=C(C1=CC=C([N+]([O-])=O)C=C1)NCCC2=CC(I)=C(OC)C(I)=C2	*L. panamensis*	IA	Low	67.82	(Restrepo et al., 2019)
**156**	S	O=C(O1)C=CC[C@@H]1C[C@@H](O)CCCC[C@@H](O)CCCCCCCCCCCCCCC	*L. panamensis*	IA	High	0.16	(Cardona et al., 2006b)
**157**	S	O=C(O1)C=CC[C@@H]1CCC[C@@H](O)CC[C@@H](O)CCCCCCCCCCCCCCC	*L. panamensis*	IA	High	0.525	(Cardona et al., 2006b)
**158**	S	O=C(O1)C=CC[C@@H]1CCCCCC[C@@H](O)CCCCCCCCCCCCCCC	*L. panamensis*	IA	High	1.657	(Cardona et al., 2006b)
**159**	S	O=C(O1)C=CC[C@@H]1CC[C@@H](O)C[C@@H](O)CCCCCCCCCCCCCC	*L. panamensis*	IA	High	0.219	(Cardona et al., 2006b)
**160**	S	O=C(O1)C=CC[C@@H]1C[C@@H](O)CC[C@@H](O)CCCCCCCCCCCCCC	*L. panamensis*	IA	High	0.609	(Cardona et al., 2006b)
**161**	S	O=C(O1)C=CC[C@@H]1CCCC[C@@H](O)CCCCCCCCCCCCCCC	*L. panamensis*	IA	High	1.522	(Cardona et al., 2006b)
**162**	S	O=C(O1)C=CC[C@@H]1C[C@@H](O)C[C@@H](O)CCCCCCCCCCCCCC	*L. panamensis*	IA	High	1.262	(Cardona et al., 2006b)
**163**	S	O=C(O1)C=CC[C@@H]1CCC[C@@H](O)CCCCCCCCCCCCCCC	*L. panamensis*	IA	High	1.42	(Cardona et al., 2006b)
**164**	S	O=C(O1)C=CC[C@@H]1CCC[C@@H](O)C[C@@H](O)CCCCCCCCCCCCCCC	*L. panamensis*	IA	High	0.707	(Cardona et al., 2006b)

Comp[a]	Ori[b]	SMILES[c]	*Leishmania* species[d]	Parasite form[e]	Leishmanicidal Potential[f]	EC_{50} (μM)[g]	Reference
**165**	S	O=C(O1)C=CC[C@@H]1C[C@@H](O)CCC[C@@H](O)CCCCCCCCCCCCCCC	*L. panamensis*	IA	High	0.071	(Cardona et al., 2006b)
**166**	S	O=C(O1)C=CC[C@@H]1CC[C@@H](O)CCCCCCCCCCCCCCC	*L. panamensis*	IA	High	0.682	(Cardona et al., 2006b)
**167**	S	O=C(O1)C=CC[C@@H]1C[C@@H](O)CCCCCCCCCCCCCC	*L. panamensis*	IA	High	1.419	(Cardona et al., 2006b)
**168**	S	O=C(O1)C=CC[C@@H]1CCCCCCCCCCCCCCC	*L. panamensis*	IA	High	3.536	(Cardona et al., 2006b)
**168**	S	O=C(O1)C=CC[C@@H]1CCCCCCCCCCCCCCC	*L. panamensis*	IA	High	0.714	(Castano et al., 2009)
**169**	S	O=C(O1)C=CC[C@H]1CCCCCCCCCCCCCCC	*L. panamensis*	IA	High	3.374	(Cardona et al., 2006b)
**169**	S	O=C(O1)C=CC[C@H]1CCCCCCCCCCCCCCC	*L. panamensis*	IA	High	0.649	(Castano et al., 2009)
**170**	S	O=C(O1)CCC[C@@H]1CCCCCCCCCCCCCCC	*L. panamensis*	IA	Low	64.134	(Cardona et al., 2006b)
**171**	S	CCCCCCCCC[C@@H]1CC=CC(O1)=O	*L. panamensis*	IA	Low	79.401	(Cardona et al., 2006b)
**172**	S	O=C(N1)CCC[C@@H]1CCCCCCCCCCCCCCC	*L. panamensis*	IA	Intermediate	11.057	(Cardona et al., 2006b)
**173**	S	O=C(O1)C=CC[C@@H]1C[C@@H](O)C[C@@H](O)C[C@@H](O)CCCCCCCCCCCCCCC	*L. panamensis*	IA	High	2.112	(Cardona et al., 2006b)
**174**	S	O=C(O1)C=CC[C@H]1C[C@@H](O)C[C@@H](O)C[C@@H](O)CCCCCCCCCCCCCCC	*L. panamensis*	IA	High	2.203	(Cardona et al., 2006b)
**175**	S	O=C(O1)C=CC[C@@H]1C[C@H](O)C[C@@H](O)C[C@@H](O)CCCCCCCCCCCCCCC	*L. panamensis*	IA	High	1.135	(Cardona et al., 2006b)
**176**	S	O=C(O1)C=CC[C@H]1C[C@H](O)C[C@@H](O)C[C@@H](O)CCCCCCCCCCCCCCC	*L. panamensis*	IA	High	1.68	(Cardona et al., 2006b)
**177**	S	O=C(O1)C=CC[C@@H]1C[C@@H](OC(C)=O)C[C@@H](OC(C)=O)C[C@@H](OC(C)=O)CCCCCCCCCCCCCCC	*L. panamensis*	IA	Intermediate	11.123	(Cardona et al., 2006b)
**178**	S	O=C(O1)C=CC[C@@H]1C[C@H](OC(C)=O)C[C@@H](OC(C)=O)C[C@@H](OC(C)=O)CCCCCCCCCCCCCCC	*L. panamensis*	IA	High	1.677	(Cardona et al., 2006b)
**179**	S	O=C(O1)C=CC[C@H]1C[C@H](OC(C)=O)C[C@@H](OC(C)=O)C[C@@H](OC(C)=O)CCCCCCCCCCCCCCC	*L. panamensis*	IA	High	2.119	(Cardona et al., 2006b)
**180**	S	O=C(O1)C=CC[C@@H]1C[C@@H](O)C[C@@H](O)CC[C@H](O)CCCCCCCCCCCCCC	*L. panamensis*	IA	High	2.112	(Cardona et al., 2006b)
**181**	S	O=C(O1)C=CC[C@H]1C[C@@H](O)C[C@@H](O)CC[C@H](O)CCCCCCCCCCCCCC	*L. panamensis*	IA	High	0.295	(Cardona et al., 2006b)
**182**	S	O=C(O1)C=CC[C@H]1C[C@@H](O)C[C@@H](O)CC[C@@H](O)CCCCCCCCCCCCCC	*L. panamensis*	IA	High	0.999	(Cardona et al., 2006b)
**183**	S	O=C(O1)C=CC[C@@H]1C[C@@H](O)C[C@@H](O)CC[C@@H](O)CCCCCCCCCCCCCC	*L. panamensis*	IA	High	0.818	(Cardona et al., 2006b)
**184**	S	CCN1C=C(C(=O)C2=CC(=C(N=C21)N3CCNCC3)F)C(=O)O	*L. panamensis*	IA	Low	54.9	(Romero et al., 2005)
**185**	S	C1CC1N2C=C(C(=O)C3=CC(=C(C=C32)N4CCNCC4)F)C(=O)O	*L. panamensis*	IA	Low	83.4	(Romero et al., 2005)
**186**	S	CCN1C=C(C(=O)C2=CC(=C(C(=C21)F)N3CCNC(C3)C)F)C(=O)O	*L. panamensis*	IA	Low	115.6	(Romero et al., 2005)
**187**	S	CC1COC2=C3N1C=C(C(=O)C3=CC(=C2N4CCN(CC4)C)F)C(=O)O	*L. panamensis*	IA	Low	151.6	(Romero et al., 2005)
**188**	S	CCN1C=C(C(=O)C2=CC(=C(C=C21)N3CCNCC3)F)C(=O)O	*L. panamensis*	IA	Low	150.2	(Romero et al., 2005)
**189**	S	CCN1C2=CC3=C(C=C2C(=O)C(=N1)C(=O)O)OCO3	*L. panamensis*	IA	Low	715	(Romero et al., 2005)

Comp[a]	Ori[b]	SMILES[c]	*Leishmania* species[d]	Parasite form[e]	Leishmanicidal Potential[f]	EC_{50} (μM)[g]	Reference
**190**	S	CCN1C=C(C(=O)C2=C1N=C(C=C2)C)C(=O)O	*L. panamensis*	IA	ND	500	(Romero et al., 2005)
**191**	S	CCOC1=CC(\C=C\C2=NC3=C(O)C=CC=C3C=C2)=CC=C1OC(C)=O	*L. panamensis*	IA	High	0.573	(Torres et al., 2020)
**192**	S	CC(=O)OC1=C(\C=C\C2=NC3=CC=CC=C3C=C2)C=C(C=C1)[N+]([O-])=O	*L. panamensis*	IA	High	7.483	(Torres et al., 2020)
**193**	S	COC1=CC(\C=C\C2=NC3=CC=CC=C3C=C2)=CC=C1	*L. panamensis*	IA	N/A		(Torres et al., 2020)
**194**	S	CC(=O)OC1=C(OC(C)=O)C(\C=C\C2=NC3=C(OC(C)=O)C=CC=C3C=C2)=CC=C1	*L. panamensis*	IA	ND	13.823	(Sánchez et al., 2014)
**195**	S	COC1=CC(\C=C\C2=NC3=CC=CC=C3C=C2)=CC=C1OC(C)=O	*L. panamensis*	IA	ND	213.399	(Sánchez et al., 2014)
**196**	S	CC(=O)OC1=C(OC(C)=O)C(\C=C\C2=NC3=CC=CC=C3C=C2)=CC=C1	*L. panamensis*	IA	High	4.033	(Sánchez et al., 2014)
**197**	S	BrC1=CC(C[C@@H](C(O)=O)N)=CC=C1O	*L. panamensis*	IA	Low	119.081	(Restrepo et al., 2018)
**198**	S	BrC1=CC(C[C@@H](C(O)=O)N)=CC(Br)=C1O	*L. panamensis*	IA	Low	106.057	(Restrepo et al., 2018)
**199**	S	ClC1=CC(C[C@@H](C(O)=O)N)=CC=C1O	*L. panamensis*	IA	Low	143.372	(Restrepo et al., 2018)
**200**	S	ClC1=CC(C[C@@H](C(O)=O)N)=CC(Cl)=C1O	*L. panamensis*	IA	Low	1128.573	(Restrepo et al., 2018)
**201**	S	BrC1=CC(C[C@@H](C(O)=O)N)=CC=C1OC	*L. panamensis*	IA	Low	301.282	(Restrepo et al., 2018)
**202**	S	BrC1=CC(C[C@@H](C(O)=O)N)=CC(Br)=C1OC	*L. panamensis*	IA	Low	394.944	(Restrepo et al., 2018)
**203**	S	ClC1=CC(C[C@@H](C(O)=O)N)=CC=C1OC	*L. panamensis*	IA	Low	154.507	(Restrepo et al., 2018)
**204**	S	ClC1=CC(C[C@@H](C(O)=O)N)=CC(Cl)=C1OC	*L. panamensis*	IA	Low	602.331	(Restrepo et al., 2018)
**205**	S	BrC1=CC(C[C@@H](C(O)=O)N(C)C)=CC=C1O	*L. panamensis*	IA	Low	240.719	(Restrepo et al., 2018)
**206**	S	ClC1=CC(C[C@@H](C(O)=O)N(C)C)=CC=C1O	*L. panamensis*	IA	Low	419.227	(Restrepo et al., 2018)
**207**	S	ClC1=CC(C[C@@H](C(O)=O)N(C)C)=CC(Cl)=C1O	*L. panamensis*	IA	Low	669.032	(Restrepo et al., 2018)
**208**	S	BrC1=CC(C[C@@H](C(O)=O)N(C)C)=CC=C1OC	*L. panamensis*	IA	Low	243.828	(Restrepo et al., 2018)
**209**	S	BrC1=CC(C[C@@H](C(O)=O)N(C)C)=CC(Br)=C1OC	*L. panamensis*	IA	Low	204.227	(Restrepo et al., 2018)
**210**	S	ClC1=CC(C[C@@H](C(O)=O)N(C)C)=CC=C1OC	*L. panamensis*	IA	Low	2078.365	(Restrepo et al., 2018)
**211**	S	ClC1=CC(C[C@@H](C(O)=O)N(C)C)=CC(Cl)=C1OC	*L. panamensis*	IA	Low	518.721	(Restrepo et al., 2018)
**212**	S	ClC1=CC(C[C@@H](C(O)=O)[N+](C)(C)C)=CC=C1O	*L. panamensis*	IA	Low	612.037	(Restrepo et al., 2018)
**212**	S	ClC1=CC(C[C@@H](C(O)=O)[N+](C)(C)C)=CC=C1O	*L. panamensis*	IA	Low	576.235	(Restrepo et al., 2018)
**213**	S	ClC1=CC(C[C@@H](C(O)=O)[N+](C)(C)C)=CC(Cl)=C1O	*L. panamensis*	IA	Low	259.099	(Restrepo et al., 2018)
**213**	S	ClC1=CC(C[C@@H](C(O)=O)[N+](C)(C)C)=CC(Cl)=C1O	*L. panamensis*	IA	Low	97.072	(Restrepo et al., 2018)
**214**	S	BrC1=CC(C[C@@H](C(O)=O)[N+](C)(C)C)=CC=C1O	*L. panamensis*	IA	Low	79.89	(Restrepo et al., 2018)
**215**	S	BrC1=CC(C[C@@H](C(O)=O)[N+](C)(C)C)=CC(Br)=C1O	*L. panamensis*	IA	Low	347.994	(Restrepo et al., 2018)
**216**	N	OC1=CC(C[C@H](N)C(O)=O)=CC(Br)=C1OC	*L. panamensis*	AA	N/A		(Galeano et al., 2012)
**216**	N	OC1=CC(C[C@H](N)C(O)=O)=CC(Br)=C1OC	*L. panamensis*	IA	N/A		(Galeano et al., 2012)
**217**	N	OC1=C(Br)C=C(C[C@H]([N+](C)(C)C)C(O)=O)C=C1	*L. panamensis*	AA	N/A		(Galeano et al., 2012)
**217**	N	OC1=C(Br)C=C(C[C@H]([N+](C)(C)C)C(O)=O)C=C1	*L. panamensis*	IA	N/A		(Galeano et al., 2012)
**218**	N	OC([C@@H]([N+](C)(C)C)CC1=CC(Br)=C(OC)C=C1)=O	*L. panamensis*	AA	N/A		(Galeano et al., 2012)
**218**	N	OC([C@@H]([N+](C)(C)C)CC1=CC(Br)=C(OC)C=C1)=O	*L. panamensis*	IA	N/A		(Galeano et al., 2012)
**219**	N	OC1=C(Br)C=C(C[C@H]([N+](C)(C)C)C(O)=O)C=C1Br	*L. panamensis*	AA	N/A		(Galeano et al., 2012)

Comp[a]	Ori[b]	SMILES[c]	*Leishmania* species[d]	Parasite form[e]	Leishmanicidal Potential[f]	EC_{50} (μM)[g]	Reference
**219**	N	OC1=C(Br)C=C(C[C@H]([N+](C)(C)C)C(O)=O)C=C1Br	*L. panamensis*	IA	N/A		(Galeano et al., 2012)
**220**	N	OC([C@@H]([N+](C)(C)C)CC1=CC(Br)=C(OC)C(Br)=C1)=O	*L. panamensis*	AA	N/A		(Galeano et al., 2012)
**220**	N	OC([C@@H]([N+](C)(C)C)CC1=CC(Br)=C(OC)C(Br)=C1)=O	*L. panamensis*	IA	N/A		(Galeano et al., 2012)
**221**	N	BrC1=C[C@@](CC#N)(O)[C@H](O)C(Br)=C1OC	*L. panamensis*	AA	N/A		(Galeano et al., 2012)
**221**	N	BrC1=C[C@@](CC#N)(O)[C@H](O)C(Br)=C1OC	*L. panamensis*	AA	N/A		(Galeano et al., 2011)
**221**	N	BrC1=C[C@@](CC#N)(O)[C@H](O)C(Br)=C1OC	*L. panamensis*	IA	N/A		(Galeano et al., 2012)
**221**	N	BrC1=C[C@@](CC#N)(O)[C@H](O)C(Br)=C1OC	*L. panamensis*	IA	N/A		(Galeano et al., 2011)
**222**	N	OC(CNC(C1=NO[C@]2(C=C(Br)C(OC)=C(Br)[C@@H]2O)C1)=O)C(O)CNC(C3=NO[C@@]4([C@@H](O)C(Br)=C(OC)C(Br)=C4)C3)=O	*L. panamensis*	AA	N/A		(Galeano et al., 2011)
**222**	N	OC(CNC(C1=NO[C@]2(C=C(Br)C(OC)=C(Br)[C@@H]2O)C1)=O)C(O)CNC(C3=NO[C@@]4([C@@H](O)C(Br)=C(OC)C(Br)=C4)C3)=O	*L. panamensis*	IA	N/A		(Galeano et al., 2011)
**223**	N	OC1=C(Br)C=C(CC[N+](C)(C)C)C=C1Br	*L. panamensis*	AA	N/A		(Galeano et al., 2011)
**223**	N	OC1=C(Br)C=C(CC[N+](C)(C)C)C=C1Br	*L. panamensis*	IA	N/A		(Galeano et al., 2011)
**224**	N	BrC1=C(OC)C(Br)=CC(CC[N+](C)(C)C)=C1	*L. panamensis*	AA	N/A		(Galeano et al., 2011)
**224**	N	BrC1=C(OC)C(Br)=CC(CC[N+](C)(C)C)=C1	*L. panamensis*	IA	N/A		(Galeano et al., 2011)
**225**	N	NC(C1=NO[C@]2(C=C(Br)C(OC)=C(Br)[C@@H]2O)C1)=O	*L. panamensis*	AA	N/A		(Galeano et al., 2011)
**225**	N	NC(C1=NO[C@]2(C=C(Br)C(OC)=C(Br)[C@@H]2O)C1)=O	*L. panamensis*	IA	N/A		(Galeano et al., 2011)
**226**	N	OC(CNC(C1=NO[C@]2(C=C(Br)C(OC)=C(Br)[C@@H]2O)C1)=O)COC3=C(Br)C=C(CCNC(C4=NO[C@@]5([C@@H](O)C(Br)=C(OC)C(Br)=C5)C4)=O)C=C3Br	*L. panamensis*	AA	N/A		(Galeano et al., 2011)
**226**	N	OC(CNC(C1=NO[C@]2(C=C(Br)C(OC)=C(Br)[C@@H]2O)C1)=O)COC3=C(Br)C=C(CCNC(C4=NO[C@@]5([C@@H](O)C(Br)=C(OC)C(Br)=C5)C4)=O)C=C3Br	*L. panamensis*	IA	N/A		(Galeano et al., 2011)
**227**	N	O=C(C1=NO[C@]2(C=C(Br)C(OC)=C(Br)[C@@H]2O)C1)NCCCOC3=C(Br)C=C(CC[N+](C)(C)C)C=C3Br	*L. panamensis*	AA	N/A		(Galeano et al., 2011)
**227**	N	O=C(C1=NO[C@]2(C=C(Br)C(OC)=C(Br)[C@@H]2O)C1)NCCCOC3=C(Br)C=C(CC[N+](C)(C)C)C=C3Br	*L. panamensis*	IA	N/A		(Galeano et al., 2011)
**228**	N	O=C(NCC(CCNC(C1=NO[C@@]2([C@H](O)C(Br)=C(OC)C(Br)=C2)C1)=O)O)C3=NO[C@@]4(C=C(Br)C(OC)=C(Br)[C@H]4O)C3	*L. panamensis*	AA	N/A		(Galeano et al., 2011)
**228**	N	O=C(NCC(CCNC(C1=NO[C@@]2([C@H](O)C(Br)=C(OC)C(Br)=C2)C1)=O)O)C3=NO[C@@]4(C=C(Br)C(OC)=C(Br)[C@H]4O)C3	*L. panamensis*	IA	N/A		(Galeano et al., 2011)
**229**	N	O[C@H](CNC(C1=NO[C@]2(C=C(Br)C(OC)=C(Br)[C@@H]2O)C1)=O)COC3=C(Br)C=C(C(O)CNC(C4=NO[C@@]5([C@@H](O)C(Br)=C(OC)C(Br)=C5)C4)=O)C=C3Br	*L. panamensis*	AA	N/A		(Galeano et al., 2011)
**229**	N	O[C@H](CNC(C1=NO[C@]2(C=C(Br)C(OC)=C(Br)[C@@H]2O)C1)=O)COC3=C(Br)C=C(C(O)CNC(C4=NO[C@@]5([C@@H](O)C(Br)=C(OC)C(Br)=C5)C4)=O)C=C3Br	*L. panamensis*	IA	N/A		(Galeano et al., 2011)
**230**	S	O=C(\C=C\C1=CC=CO1)C1=CC=C(OCCCOC2=CC3=C(C=C2)C(=O)C=CO3)C=C1	*L. panamensis*	IA	Low	108.28	(García et al., 2018)

Comp[a]	Ori[b]	SMILES[c]	*Leishmania* species[d]	Parasite form[e]	Leishmanicidal Potential[f]	EC_{50} (μM)[g]	Reference
**231**	S	O=C(\C=C\C1=CC=CO1)C1=CC=C(OCCCCOC2=CC3=C(C=C2)C(=O)C=CO3)C=C1	*L. panamensis*	IA	ND	44.23	(García et al., 2018)
**232**	S	O=C(\C=C\C1=CC=CO1)C1=CC=C(OCCCCCOC2=CC3=C(C=C2)C(=O)C=CO3)C=C1	*L. panamensis*	IA	Low	125.38	(García et al., 2018)
**233**	S	O=C(\C=C\C1=CC=CO1)C1=CC=C(OCCCCCCCCOC2=CC3=C(C=C2)C(=O)C=CO3)C=C1	*L. panamensis*	IA	Low	44.02	(García et al., 2018)
**234**	S	O=C(\C=C\C1=CC=CO1)C1=CC=C(OCCCCCCCCCOC2=CC3=C(C=C2)C(=O)C=CO3)C=C1	*L. panamensis*	IA	Low	46	(García et al., 2018)
**235**	S	O=C(\C=C\C1=CC=CO1)C1=CC=C(OCCCCCCCCCCCCOC2=CC3=C(C=C2)C(=O)C=CO3)C=C1	*L. panamensis*	IA	ND	17.72	(García et al., 2018)
**236**	S	CN1C=C[N+](CCCOC2=CC=C(C=C2)C(=O)\C=C\C2=CC=CO2)=C1	*L. panamensis*	IA	High	4.65	(García et al., 2018)
**237**	S	CN1C=C[N+](CCCCOC2=CC=C(C=C2)C(=O)\C=C\C2=CC=CO2)=C1	*L. panamensis*	IA	Intermediate	21.78	(García et al., 2018)
**238**	S	CN1C=C[N+](CCCCCOC2=CC=C(C=C2)C(=O)\C=C\C2=CC=CO2)=C1	*L. panamensis*	IA	Intermediate	16.43	(García et al., 2018)
**239**	S	CN1C=C[N+](CCCCCCCCOC2=CC=C(C=C2)C(=O)\C=C\C2=CC=CO2)=C1	*L. panamensis*	IA	High	8.39	(García et al., 2018)
**240**	S	CN1C=C[N+](CCCCCCCCCOC2=CC=C(C=C2)C(=O)\C=C\C2=CC=CO2)=C1	*L. panamensis*	IA	High	0.78	(García et al., 2018)
**241**	S	CN1C=C[N+](CCCCCCCCCCCCOC2=CC=C(C=C2)C(=O)\C=C\C2=CC=CO2)=C1	*L. panamensis*	IA	High	2.12	(García et al., 2018)
**242**	S	O=C(\C=C\C1=CC=CO1)C1=CC=C(OCCCCOC2=CC=CC3=C2N=CC=C3)C=C1	*L. panamensis*	IA	Low	33.64	(García et al., 2018)
**243**	S	O=C(\C=C\C1=CC=CO1)C1=CC=C(OCCCCCOC2=CC=CC3=C2N=CC=C3)C=C1	*L. panamensis*	IA	Intermediate	13.78	(García et al., 2018)
**244**	S	O=C(\C=C\C1=CC=CO1)C1=CC=C(OCCCCCCCCOC2=CC=CC3=C2N=CC=C3)C=C1	*L. panamensis*	IA	Low	58.33	(García et al., 2018)
**245**	S	O=C(\C=C\C1=CC=CO1)C1=CC=C(OCCCCCCCCCOC2=CC=CC3=C2N=CC=C3)C=C1	*L. panamensis*	IA	ND	20.69	(García et al., 2018)
**246**	S	O=C(\C=C\C1=CC=CO1)C1=CC=C(OCCCCCCCCCCCCOC2=CC=CC3=C2N=CC=C3)C=C1	*L. panamensis*	IA	Low	207.36	(García et al., 2018)
**247**	S	OC1=CC=C(C=C1)C(=O)\C=C\C1=CC=CO1	*L. panamensis*	IA	Low	71.28	(García et al., 2018)
**248**	S	OC1=C2N=CC=CC2=CC=C1	*L. panamensis*	IA	High	2.48	(García et al., 2018)
**249**	S	OC1=CC=C2C(=O)C=COC2=C1	*L. panamensis*	IA	Low	718.45	(García et al., 2018)
**250**	S	N#CC1=CC2=CC=CC=C2SC1	*L. panamensis*	IA	High	6.1	(Ortiz et al., 2020)
**251**	S	COC(=O)C1=CC2=CC=CC=C2SC1	*L. panamensis*	IA	Low	126	(Ortiz et al., 2020)
**252**	S	CC(=O)C1=CC2=CC=CC=C2SC1C1=CC=CC=C1	*L. panamensis*	IA	ND	2.6	(Ortiz et al., 2020)
**253**	S	CCCCCC1SC2=CC=CC=C2C=C1C(C)=O	*L. panamensis*	IA	High	8.3	(Ortiz et al., 2020)
**254**	S	FCC(=O)C1=CC2=CC=CC=C2SC1C1=CC=CC=C1	*L. panamensis*	IA	Low	29.6	(Ortiz et al., 2020)

Comp[a]	Ori[b]	SMILES[c]	*Leishmania* species[d]	Parasite form[e]	Leishmanicidal Potential[f]	EC_{50} (μM)[g]	Reference
**255**	S	[H]C(=O)C1=CC2=CC=CC=C2SC1C1=CC=CC=C1	*L. panamensis*	IA	ND	23.8	(Ortiz et al., 2020)
**256**	S	[H]C(=O)C1=CC2=CC=CC=C2SC1C1=CC=C(F)C=C1	*L. panamensis*	IA	Low	29.7	(Ortiz et al., 2020)
**257**	S	[H]C(=O)C1=CC2=CC=CC=C2SC1C1=CC=C(Cl)C=C1	*L. panamensis*	IA	Low	37	(Ortiz et al., 2020)
**258**	S	[H]C(=O)C1=CC2=CC=CC=C2SC1C1=CC=CC=C1[N+]([O-])=O	*L. panamensis*	IA	High	9.2	(Ortiz et al., 2020)
**259**	S	O=C(C1=CC=CC=C1)C1=CC2=CC=CC=C2SC1C1=CC=CC=C1	*L. panamensis*	IA	Low	43.9	(Ortiz et al., 2020)
**260**	S	FC(F)(F)C1=CC=C(C=C1)C1SC2=CC=CC=C2C=C1C(=O)C1=CC=C(Cl)C=C1	*L. panamensis*	IA	Low	37.9	(Ortiz et al., 2020)
**261**	S	[O-][N+](=O)C1=CC=C(C=C1)C1SC2=CC=CC=C2C=C1C(=O)C1=CC=C(Cl)C=C1	*L. panamensis*	IA	Low	38.1	(Ortiz et al., 2020)
**262**	S	O=C1C(CSC2=CC=CC=C12)C#N	*L. panamensis*	IA	Low	107.7	(Ortiz et al., 2020)
**263**	S	COC(=O)C1CSC2=CC=CC=C2C1=O.COC(=O)C1=C(O)C2=CC=CC=C2SC1	*L. panamensis*	IA	Low	133.6	(Ortiz et al., 2020)
**264**	S	CCCCCC1SC2=CC=CC=C2C(O)=C1C(C)=O	*L. panamensis*	IA	Intermediate	12.5	(Ortiz et al., 2020)
**265**	S	OC(=O)C1CC(=O)C2=CC=CC=C2S1	*L. panamensis*	IA	Low	414.1	(Ortiz et al., 2020)
**265**	S	OC(=O)C1CC(=O)C2=CC=CC=C2S1	*L. panamensis*	IA	ND	96.14	
**266**	S	OC(=O)C1CC(=O)C2=CC(F)=CC=C2S1	*L. panamensis*	IA	Low	3517.1	(Ortiz et al., 2020)
**267**	S	COC1=CC=C2SC(CC(=O)C2=C1)C(O)=O	*L. panamensis*	IA	Low	493.6	(Ortiz et al., 2020)
**268**	S	COC1=CC=C2C(=O)CC(SC2=C1)C(O)=O	*L. panamensis*	IA	Low	277	(Ortiz et al., 2020)
**269**	S	CCOC(=O)C1CC(=O)C2=CC=CC=C2S1	*L. panamensis*	IA	ND	68.5	(Ortiz et al., 2020)
**270**	S	CCCCOC(=O)C1CC(=O)C2=CC=CC=C2S1	*L. panamensis*	IA	ND	189.3	(Ortiz et al., 2020)
**271**	S	CCCCCCOC(=O)C1CC(=O)C2=CC=CC=C2S1	*L. panamensis*	IA	ND	102.7	(Ortiz et al., 2020)
**272**	S	CCCCCCCCCCOC(=O)C1CC(=O)C2=CC=CC=C2S1	*L. panamensis*	IA	ND	143.6	(Ortiz et al., 2020)
**273**	S	CCCCCCNC(=O)C1CC(=O)C2=CC=CC=C2S1	*L. panamensis*	IA	Low	216.2	(Ortiz et al., 2020)
**274**	S	CCCCCCCCCCCCNC(=O)C1CC(=O)C2=CC=CC=C2S1	*L. panamensis*	IA	ND	80	(Ortiz et al., 2020)
**275**	S	CC1=CC=CC=C1NC(=O)C1CC(=O)C2=CC=CC=C2S1	*L. panamensis*	IA	ND	101	(Ortiz et al., 2020)
**276**	S	OC(=O)[C@@H]1CC(=O)C2=CC=CC=C2S1	*L. panamensis*	IA	Low	505.3	(Ortiz et al., 2020)
**277**	S	OC(=O)[C@H]1CC(=O)C2=CC=CC=C2S1	*L. panamensis*	IA	Low	53.5	(Ortiz et al., 2020)
**278**	S	CCCCOC(=O)[C@@H]1CC(=O)C2=CC=CC=C2S1	*L. panamensis*	IA	Low	368	(Ortiz et al., 2020)
**279**	S	CCCCOC(=O)[C@H]1CC(=O)C2=CC=CC=C2S1	*L. panamensis*	IA	Low	53	(Ortiz et al., 2020)
**280**	S	CCOC(=O)C1C\C(=N/NC(=O)C2=CC=CC=C2)C2=CC=CC=C2S1	*L. panamensis*	IA	Low	28.5	(Ortiz et al., 2020)
**281**	S	CCOC(=O)C1C\C(=N/NC(=O)C2=NC=CC=C2)C2=CC=CC=C2S1	*L. panamensis*	IA	Low	53.2	(Ortiz et al., 2020)
**282**	S	NC(=O)C(=O)N\N=C1/CC(SC2=CC=CC=C12)C(O)=O	*L. panamensis*	IA	ND	188.6	(Ortiz et al., 2020)
**283**	S	O=C(N1CCN(C2=C([N+]([O-])=O)C=C(C(F)(F)F)C=C2)CC1)NC3=[N+]([O-])C4=CC=C(Cl)C=C4[N+]([O-])=C3C#N	*L. infantum*	AA	Intermediate	21.8	(Barea et al., 2012)

Comp[a]	Ori[b]	SMILES[c]	*Leishmania* species[d]	Parasite form[e]	Leishmanicidal Potential[f]	EC_{50} (μM)[g]	Reference
**284**	S	O=C(N1CCN(C2=C([N+]([O-])=O)C=C(C(F)(F)F)C=C2)CC1)NC3=[N+]([O-])C4=CC=C(C)C=C4[N+]([O-])=C3C#N	*L. infantum*	AA	Low	36.3	(Barea et al., 2012)
**285**	S	O=C(N1CCN(C2=C([N+]([O-])=O)C=C(C(F)(F)F)C=C2)CC1)NC3=[N+]([O-])C4=CC=C(F)C=C4[N+]([O-])=C3C#N	*L. infantum*	AA	Low	41.1	(Barea et al., 2012)
**286**	S	O=C(N1CCN(C2=C([N+]([O-])=O)C=C(C(F)(F)F)C=C2)CC1)NC3=[N+]([O-])C4=CC(Cl)=C(Cl)C=C4[N+]([O-])=C3C#N	*L. infantum*	AA	Intermediate	22.7	(Barea et al., 2012)
**287**	S	O=C(N1CCN(C2=CC=C(C(F)(F)F)C=C2)CC1)NC3=[N+]([O-])C4=CC=C(Cl)C=C4[N+]([O-])=C3C#N	*L. infantum*	AA	High	7.6	(Barea et al., 2012)
**288**	S	O=C(N1CCN(C2=CC=C(C(F)(F)F)C=C2)CC1)NC3=[N+]([O-])C4=CC=C(C)C=C4[N+]([O-])=C3C#N	*L. infantum*	AA	Intermediate	23.3	(Barea et al., 2012)
**289**	S	O=C(N1CCN(C2=CC=C(C(F)(F)F)C=C2)CC1)NC3=[N+]([O-])C4=CC=C(F)C=C4[N+]([O-])=C3C#N	*L. infantum*	AA	Low	28.8	(Barea et al., 2012)
**290**	S	O=C(N1CCN(C2=CC=C(C(F)(F)F)C=C2)CC1)NC3=[N+]([O-])C4=CC(Cl)=C(Cl)C=C4[N+]([O-])=C3C#N	*L. infantum*	AA	High	5.7	(Barea et al., 2012)
**291**	S	O=C(N1CCN(C2=CC=C(F)C=C2)CC1)NC3=[N+]([O-])C4=CC=C(C)C=C4[N+]([O-])=C3C#N	*L. infantum*	AA	Intermediate	23	(Barea et al., 2012)
**292**	S	O=C(N1CCN(C2=CC=C(F)C=C2)CC1)NC3=[N+]([O-])C4=CC=C(F)C=C4[N+]([O-])=C3C#N	*L. infantum*	AA	Low	31.3	(Barea et al., 2012)
**293**	S	O=C(N1CCN(C2=CC=C(OC)C=C2)CC1)NC3=[N+]([O-])C4=CC=C(Cl)C=C4[N+]([O-])=C3C#N	*L. infantum*	AA	Intermediate	18.8	(Barea et al., 2012)
**294**	S	O=C(N1CCN(C2=CC=C(OC)C=C2)CC1)NC3=[N+]([O-])C4=CC=C(C)C=C4[N+]([O-])=C3C#N	*L. infantum*	AA	Low	30	(Barea et al., 2012)
**295**	S	O=C(N1CCN(C2=CC=C(OC)C=C2)CC1)NC3=[N+]([O-])C4=CC(Cl)=C(Cl)C=C4[N+]([O-])=C3C#N	*L. infantum*	AA	Intermediate	10.9	(Barea et al., 2012)
**296**	N	O=C1C=CC[C@@H](/C=C/C=C/C(CC)=O)O1	*L. panamensis*	IA	High	9.219	(Carmona et al., 2003)
**297**	N	O=C1C=CC[C@H](/C=C\C=C\C(CC)=O)O1	*L. panamensis*	IA	High	2.038	(Carmona et al., 2003)
**298**	S	CC1CC(=O)C2=CC=CC=C2S1	*L. panamensis*	IA	Low	444.6	(Vargas et al., 2017)
**299**	S	CC1CC(=O)C2=CC(F)=CC=C2S1	*L. panamensis*	IA	Low	422	(Vargas et al., 2017)
**300**	S	FC1=CC=C2SCCC(=O)C2=C1	*L. panamensis*	IA	ND	109.88	(Vargas et al., 2017)
**301**	S	O=C1CCSC2=C3C(=O)CCSC3=CC=C12	*L. panamensis*	IA	ND	80	(Vargas et al., 2017)
**302**	S	[O-][N+](=O)C1=CC=C2SCCC(=O)C2=C1	*L. panamensis*	IA	ND	95.59	(Vargas et al., 2017)
**303**	S	NC1=CC=C2SCCC(=O)C2=C1	*L. panamensis*	IA	ND	111.58	(Vargas et al., 2017)
**304**	S	O=C1CC(SC2=CC=CC=C12)C1=CC=CC=C1	*L. panamensis*	IA	Low	44.06	(Vargas et al., 2017)
**305**	S	FC(F)(F)C1=CC=C(C=C1)C1CC(=O)C2=CC=CC=C2S1	*L. panamensis*	IA	Low	61.43	(Vargas et al., 2017)
**306**	S	FC1=CC=C2SC(CC(=O)C2=C1)C1=CC=C(C=C1)C(F)(F)F	*L. panamensis*	IA	Low	80.29	(Vargas et al., 2017)
**307**	S	ClC1=CC=C(C=C1)C1CC(=O)C2=CC=CC=C2S1	*L. panamensis*	IA	ND	72.79	(Vargas et al., 2017)

Comp[a]	Ori[b]	SMILES[c]	*Leishmania* species[d]	Parasite form[e]	Leishmanicidal Potential[f]	EC_{50} (μM)[g]	Reference
**308**	S	FC1=CC=C(C=C1)C1CC(=O)C2=CC=CC=C2S1	*L. panamensis*	IA	Low	93.57	(Vargas et al., 2017)
**309**	S	[O-][N+](=O)C1=CC=C(C=C1)C1CC(=O)C2=CC=CC=C2S1	*L. panamensis*	IA	ND	70.1	(Vargas et al., 2017)
**310**	S	[O-][N+](=O)C1=CC=C2SC=CC(=O)C2=C1	*L. panamensis*	IA	Low	36.76	(Vargas et al., 2017)
**311**	S	FC(F)(F)C1=CC=C(C=C1)C1=CC(=O)C2=CC=CC=C2S1	*L. panamensis*	IA	Low	160.97	(Vargas et al., 2017)
**312**	S	FC1=CC=C2SC(=CC(=O)C2=C1)C1=CC=C(C=C1)C(F)(F)F	*L. panamensis*	IA	Low	52.26	(Vargas et al., 2017)
**313**	S	ClC1=CC=C(C=C1)C1=CC(=O)C2=CC=CC=C2S1	*L. panamensis*	IA	ND	73.3	(Vargas et al., 2017)
**314**	S	FC1=CC=C(C=C1)C1=CC(=O)C2=CC=CC=C2S1	*L. panamensis*	IA	ND	78.04	(Vargas et al., 2017)
**315**	S	[O-][N+](=O)C1=CC=C(C=C1)C1=CC(=O)C2=CC=CC=C2S1	*L. panamensis*	IA	ND	70.6	(Vargas et al., 2017)
**316**	S	CC1CC(=O)C2=CC=CC=C2S1(=O)=O	*L. panamensis*	IA	ND	95.22	(Vargas et al., 2017)
**317**	S	CC1CC(=O)C2=CC(F)=CC=C2S1(=O)=O	*L. panamensis*	IA	ND	87.71	(Vargas et al., 2017)
**318**	S	FC1=CC=C2C(=C1)C(=O)CCS2(=O)=O	*L. panamensis*	IA	ND	93.45	(Vargas et al., 2017)
**319**	S	O=C1CC(C2=CC=CC=C2)S(=O)(=O)C2=CC=CC=C12	*L. panamensis*	IA	Low	217.02	(Vargas et al., 2017)
**320**	S	FC(F)(F)C1=CC=C(C=C1)C1CC(=O)C2=CC=CC=C2S1(=O)=O	*L. panamensis*	IA	Low	84.81	(Vargas et al., 2017)
**321**	S	FC1=CC=C2C(=C1)C(=O)CC(C1=CC=C(C=C1)C(F)(F)F)S2(=O)=O	*L. panamensis*	IA	Low	79.11	(Vargas et al., 2017)
**322**	S	ClC1=CC=C(C=C1)C1CC(=O)C2=CC=CC=C2S1(=O)=O	*L. panamensis*	IA	Low	137.09	(Vargas et al., 2017)
**323**	S	FC1=CC=C(C=C1)C1CC(=O)C2=CC=CC=C2S1(=O)=O	*L. panamensis*	IA	ND	68.9	(Vargas et al., 2017)
**324**	S	[O-][N+](=O)C1=CC=C(C=C1)C1CC(=O)C2=CC=CC=C2S1(=O)=O	*L. panamensis*	IA	ND	63.03	(Vargas et al., 2017)
**325**	S	O=C1C=C(C2=CC=CC=C2)S(=O)(=O)C2=CC=CC=C12	*L. panamensis*	IA	High	7.56	(Vargas et al., 2017)
**326**	S	FC(F)(F)C1=CC=C(C=C1)C1=CC(=O)C2=CC=CC=C2S1(=O)=O	*L. panamensis*	IA	High	7.3	(Vargas et al., 2017)
**327**	S	FC1=CC=C2C(=C1)C(=O)C=C(C1=CC=C(C=C1)C(F)(F)F)S2(=O)=O	*L. panamensis*	IA	High	3.24	(Vargas et al., 2017)
**328**	S	ClC1=CC=C(C=C1)C1=CC(=O)C2=CC=CC=C2S1(=O)=O	*L. panamensis*	IA	High	7.47	(Vargas et al., 2017)
**329**	S	FC1=CC=C(C=C1)C1=CC(=O)C2=CC=CC=C2S1(=O)=O	*L. panamensis*	IA	High	6.72	(Vargas et al., 2017)
**330**	S	[O-][N+](=O)C1=CC=C(C=C1)C1=CC(=O)C2=CC=CC=C2S1(=O)=O	*L. panamensis*	IA	High	7.23	(Vargas et al., 2017)
**331**	S	O=C1CCOC2=CC=CC=C21	*L. panamensis*	IA	Low	203.982	(Upegui et al., 2019)
**331**	S	O=C1CCOC2=CC=CC=C21	*L. braziliensis*	IA	Low	224.245	(Upegui et al., 2019)
**332**	S	O=C1CCSC2=CC=CC=C21	*L. panamensis*	IA	Low	290.192	(Upegui et al., 2019)
**332**	S	O=C1CCSC2=CC=CC=C21	*L. braziliensis*	IA	Low	163.995	(Upegui et al., 2019)
**332**	S	O=C1CCSC2=CC=CC=C21	*L. panamensis*	IA	Low	343.8	(Vargas et al., 2018)
**333**	S	O=C(C1=CC=CC=C1)N/N=C2CCSC3=CC=CC=C3\2	*L. panamensis*	IA	Low	88.626	(Upegui et al., 2019)
**333**	S	O=C(C1=CC=CC=C1)N/N=C2CCSC3=CC=CC=C3\2	*L. braziliensis*	IA	Low	82.6	(Upegui et al., 2019)
**333**	S	O=C(C1=CC=CC=C1)N/N=C2CCSC3=CC=CC=C3\2	*L. panamensis*	IA	Low	63.7	(Vargas et al., 2018)
**334**	S	O=C(C1=CC=CC=C1)N/N=C2CCOC3=CC=CC=C3\2	*L. panamensis*	IA	Low	75.534	(Upegui et al., 2019)
**334**	S	O=C(C1=CC=CC=C1)N/N=C2CCOC3=CC=CC=C3\2	*L. braziliensis*	IA	Low	45.471	(Upegui et al., 2019)
**335**	S	O=C(C1=CC=CC=C1)N/N=C2CCS(C3=CC=CC=C3\2)(=O)=O	*L. panamensis*	IA	ND	159.199	(Upegui et al., 2019)
**335**	S	O=C(C1=CC=CC=C1)N/N=C2CCS(C3=CC=CC=C3\2)(=O)=O	*L. braziliensis*	IA	ND	159.199	(Upegui et al., 2019)
**336**	S	O=C(C1=CC=NC=C1)N/N=C2CCOC3=CC=CC=C3\2	*L. panamensis*	IA	ND	187.195	(Upegui et al., 2019)
**336**	S	O=C(C1=CC=NC=C1)N/N=C2CCOC3=CC=CC=C3\2	*L. braziliensis*	IA	ND	187.195	(Upegui et al., 2019)

Comp[a]	Ori[b]	SMILES[c]	*Leishmania* species[d]	Parasite form[e]	Leishmanicidal Potential[f]	EC_{50} (μM)[g]	Reference
**337**	N	O=C1OC2=C([C@@H]3[C@H](O3)C(C)=C)C(OC[C@@H]4C(C)(O4)C)=CC=C2C=C1	*L. panamensis*	AA	Low	30.171	(Arango et al., 2010)
**338**	N	O=C1OC2=C(C3=COC=C3C)C(OC)=CC=C2C=C1	*L. panamensis*	AA	Low	41.004	(Arango et al., 2010)
**339**	N	O=C1OC2=C([C@@H]3[C@H](O3)C(C)=C)C(OC)=CC=C2C=C1	*L. panamensis*	AA	Low	54.632	(Arango et al., 2010)
**340**	N	O=C1OC2=C(/C(C=O)=C(C)\C)C(OC)=CC=C2C=C1	*L. panamensis*	AA	ND	387.463	(Arango et al., 2010)
**341**	S	COC1=CC(\C=C\C2=NC3=C(C=CC=C3)C=C2)=C(OC)C=C1	*L. panamensis*	AA	Intermediate	12.709	(Mesa V. et al., 2008)
**341**	S	COC1=CC(\C=C\C2=NC3=C(C=CC=C3)C=C2)=C(OC)C=C1	*L. panamensis*	IA	High	4.809	(Mesa V. et al., 2008)
**342**	S	COC1=CC(CCC2=NC3=C(C=CC=C3)C=C2)=C(OC)C=C1	*L. panamensis*	AA	Low	106.092	(Mesa V. et al., 2008)
**342**	S	COC1=CC(CCC2=NC3=C(C=CC=C3)C=C2)=C(OC)C=C1	*L. panamensis*	IA	N/A		(Mesa V. et al., 2008)
**343**	S	COC1=CC=CC(\C=C\C2=NC3=C(C=CC=C3)C=C2)=C1OC	*L. panamensis*	AA	Intermediate	15.457	(Mesa V. et al., 2008)
**343**	S	COC1=CC=CC(\C=C\C2=NC3=C(C=CC=C3)C=C2)=C1OC	*L. panamensis*	IA	High	6.183	(Mesa V. et al., 2008)
**344**	S	COC1=CC=CC(CCC2=NC3=C(C=CC=C3)C=C2)=C1OC	*L. panamensis*	AA	Low	80.507	(Mesa V. et al., 2008)
**344**	S	COC1=CC=CC(CCC2=NC3=C(C=CC=C3)C=C2)=C1OC	*L. panamensis*	IA	N/A		(Mesa V. et al., 2008)
**345**	S	CC(=O)NC1=CC=C(\C=C\C2=NC3=C(C=CC=C3)C=C2)C=C1	*L. panamensis*	AA	Low	66.29	(Mesa V. et al., 2008)
**345**	S	CC(=O)NC1=CC=C(\C=C\C2=NC3=C(C=CC=C3)C=C2)C=C1	*L. panamensis*	IA	High	5.9	(Mesa V. et al., 2008)
**346**	S	CC(=O)NC1=CC=C(CCC2=NC3=C(C=CC=C3)C=C2)C=C1	*L. panamensis*	AA	Low	204.383	(Mesa V. et al., 2008)
**346**	S	CC(=O)NC1=CC=C(CCC2=NC3=C(C=CC=C3)C=C2)C=C1	*L. panamensis*	IA	N/A		(Mesa V. et al., 2008)
**347**	S	[H][C@]12C[C@H](N(O1)C1=C3C=CC=CC3=CC=C1C2)C1=CC=CC=C1	*L. chagasi*	P	Intermediate	21.2	(Palma et al., 2009)
**348**	S	O[C@H]1C[C@H](NC2=C3C=CC=CC3=CC=C2C1)C1=CC=CC=C1	*L. chagasi*	P	Low	46	(Palma et al., 2009)
**349**	S	[H][C@]12C[C@H](N(O1)C1=C3C=CC=CC3=CC=C1C2)C1=CC=C(F)C=C1	*L. chagasi*	P	Low	70.4	(Palma et al., 2009)
**350**	S	O[C@H]1C[C@H](NC2=C3C=CC=CC3=CC=C2C1)C1=CC=C(F)C=C1	*L. chagasi*	P	Intermediate	21	(Palma et al., 2009)
**351**	S	[H][C@]12C[C@H](N(O1)C1=C3C=CC=CC3=CC=C1C2)C1=CC=C(Cl)C=C1	*L. chagasi*	P	Low	35.4	(Palma et al., 2009)
**352**	S	O[C@H]1C[C@H](NC2=C3C=CC=CC3=CC=C2C1)C1=CC=C(Cl)C=C1	*L. chagasi*	P	Low	25.9	(Palma et al., 2009)
**353**	S	[H][C@]12C[C@H](N(O1)C1=C3C=CC=CC3=CC=C1C2)C1=CC=C(Br)C=C1	*L. chagasi*	P	Low	73.4	(Palma et al., 2009)
**354**	S	O[C@H]1C[C@H](NC2=C3C=CC=CC3=CC=C2C1)C1=CC=C(Br)C=C1	*L. chagasi*	P	Intermediate	18.5	(Palma et al., 2009)
**355**	S	[H][C@]12C[C@H](N(O1)C1=C3C=CC=CC3=CC=C1C2)C1=CC=CC(Cl)=C1	*L. chagasi*	P	Low	32.7	(Palma et al., 2009)
**356**	S	O[C@H]1C[C@H](NC2=C3C=CC=CC3=CC=C2C1)C1=CC=CC(Cl)=C1	*L. chagasi*	P	High	6	(Palma et al., 2009)
**357**	S	[H][C@]12C[C@H](N(O1)C1=C3C=CC=CC3=CC=C1C2)C1=CC=CC(Br)=C1	*L. chagasi*	P	Intermediate	10.4	(Palma et al., 2009)
**358**	S	O[C@H]1C[C@H](NC2=C3C=CC=CC3=CC=C2C1)C1=CC=CC(Br)=C1	*L. chagasi*	P	Intermediate	18.3	(Palma et al., 2009)
**359**	S	[H][C@]12C[C@H](N(O1)C1=C3C=CC=CC3=CC=C1C2)C1=CC=CC(OC)=C1	*L. chagasi*	P	Intermediate	16.4	(Palma et al., 2009)

Comp[a]	Ori[b]	SMILES[c]	*Leishmania* species[d]	Parasite form[e]	Leishmanicidal Potential[f]	EC50 (μM)[g]	Reference
**360**	S	COC1=CC(=CC=C1)[C@@H]1C[C@H](O)CC2=CC=C3C=CC=CC3=C2N1	*L. chagasi*	P	Intermediate	17.9	(Palma et al., 2009)
**361**	S	[H][C@]12C[C@H](N(O1)C1=C3C=CC=CC3=CC=C1C2)C1=CC=CC(C)=C1	*L. chagasi*	P	Low	35.2	(Palma et al., 2009)
**362**	S	CC1=CC(=CC=C1)[C@@H]1C[C@H](O)CC2=CC=C3C=CC=CC3=C2N1	*L. chagasi*	P	Low	41.4	(Palma et al., 2009)
**363**	S	[H][C@]12C[C@H](N(O1)C1=C3C=CC=CC3=CC=C1C2)C1=CC=CC=C1Cl	*L. chagasi*	P	Low	183.8	(Palma et al., 2009)
**364**	S	O[C@H]1C[C@H](NC2=C3C=CC=CC3=CC=C2C1)C1=CC=CC=C1Cl	*L. chagasi*	P	Low	30.6	(Palma et al., 2009)
**365**	S	[H][C@]12C[C@H](N(O1)C1=C3C=CC=CC3=CC=C1C2)C1=CC=C(Cl)C=C1Cl	*L. chagasi*	P	Intermediate	22.1	(Palma et al., 2009)
**366**	S	O[C@H]1C[C@H](NC2=C3C=CC=CC3=CC=C2C1)C1=CC=C(Cl)C=C1Cl	*L. chagasi*	P	Intermediate	11	(Palma et al., 2009)
**367**	S	[H][C@]12C[C@H](N(O1)C1=C3C=CC=CC3=CC=C1C2)C1=CC=CC=C1[N+]([O-])=O	*L. chagasi*	P	Low	40.9	(Palma et al., 2009)
**368**	S	OC1=C(\C=N\NC(=O)COC2=CC(Cl)=CC=C2OC2=CC=C(Cl)C=C2Cl)C=CC=C1	*L. panamensis*	IA	ND	1.29	(Vergara et al., 2017)
**369**	S	OC1=CC=CC(\C=N\NC(=O)COC2=CC(Cl)=CC=C2OC2=CC=C(Cl)C=C2Cl)=C1O	*L. panamensis*	IA	Intermediate	24.7	(Vergara et al., 2017)
**370**	S	OC1=C(O)C=C(\C=N\NC(=O)COC2=CC(Cl)=CC=C2OC2=CC=C(Cl)C=C2Cl)C=C1	*L. panamensis*	IA	Low	25.08	(Vergara et al., 2017)
**371**	S	OC1=CC(O)=C(\C=N\NC(=O)COC2=CC(Cl)=CC=C2OC2=CC=C(Cl)C=C2Cl)C=C1	*L. panamensis*	IA	High	1.64	(Vergara et al., 2017)
**372**	S	OC1=CC(\C=N\NC(=O)COC2=CC(Cl)=CC=C2OC2=CC=C(Cl)C=C2Cl)=C(O)C=C1	*L. panamensis*	IA	Low	70.33	(Vergara et al., 2017)
**373**	S	COC1=C(O)C=CC(\C=N\NC(=O)COC2=CC(Cl)=CC=C2OC2=CC=C(Cl)C=C2Cl)=C1	*L. panamensis*	IA	High	6.88	(Vergara et al., 2017)
**374**	S	COC1=CC(O)=C(\C=N\NC(=O)COC2=CC(Cl)=CC=C2OC2=CC=C(Cl)C=C2Cl)C=C1	*L. panamensis*	IA	High	2.36	(Vergara et al., 2017)
**375**	S	OC1=C(O)C(O)=C(\C=N\NC(=O)COC2=CC(Cl)=CC=C2OC2=CC=C(Cl)C=C2Cl)C=C1	*L. panamensis*	IA	Low	39.24	(Vergara et al., 2017)
**376**	S	COC1=C(O)C(O)=CC(\C=N\NC(=O)COC2=CC(Cl)=CC=C2OC2=CC=C(Cl)C=C2Cl)=C1	*L. panamensis*	IA	Intermediate	11.92	(Vergara et al., 2017)
**377**	S	COC1=CC(\C=N\NC(=O)COC2=CC(Cl)=CC=C2OC2=CC=C(Cl)C=C2Cl)=CC(OC)=C1O	*L. panamensis*	IA	High	9.3	(Vergara et al., 2017)
**378**	S	OC1=CC(O)=C(\C=N\NC(=O)COC2=CC(Cl)=CC=C2OC2=CC=C(Cl)C=C2Cl)C(O)=C1	*L. panamensis*	IA	Low	45.61	(Vergara et al., 2017)
**379**	S	COC(=O)\C=C\C1=CC(OC)=C(OC)C=C1	*L. panamensis*	AA	Low	247.648	(Otero et al., 2014a)
**380**	S	CCCOC(=O)\C=C\C1=CC(OC)=C(OC)C=C1	*L. panamensis*	AA	Low	131.936	(Otero et al., 2014a)

Comp[a]	Ori[b]	SMILES[c]	*Leishmania* species[d]	Parasite form[e]	Leishmanicidal Potential[f]	EC_{50} (μM)[g]	Reference
**381**	S	CCCCCOC(=O)\C=C\C1=CC(OC)=C(OC)C=C1	*L. panamensis*	AA	Low	136.976	(Otero et al., 2014a)
**382**	S	CCCCCCOC(=O)\C=C\C1=CC(OC)=C(OC)C=C1	*L. panamensis*	AA	Low	205.362	(Otero et al., 2014a)
**383**	S	CCCCCCCCOC(=O)\C=C\C1=CC(OC)=C(OC)C=C1	*L. panamensis*	AA	ND	312.306	(Otero et al., 2014a)
**384**	S	CCCCCCCCCCCOC(=O)\C=C\C1=CC=C(OC)C(OC)=C1	*L. panamensis*	AA	Low	156.806	(Otero et al., 2014a)
**385**	S	CCCCCCCCCCCCCCCCCCOC(=O)\C=C\C1=CC=C(OC)C(OC)=C1	*L. panamensis*	AA	Low	235.036	(Otero et al., 2014a)
**386**	S	CCCCCCCCCCCCCCCCOC(=O)\C=C\C1=CC=C(OC)C(OC)=C1	*L. panamensis*	AA	Low	128.145	(Otero et al., 2014a)
**387**	S	CCCCCCCCCCCCCCCCOC(=O)\C=C\C1=C(OC)C(OC)=CC=C1	*L. panamensis*	AA	ND	231.308	(Otero et al., 2014a)
**388**	S	CCCCCCCCCCCCCCCCOC(=O)\C=C\C1=C(OC)C=CC(OC)=C1	*L. panamensis*	AA	ND	231.308	(Otero et al., 2014a)
**389**	S	CCCCCCCCCCCCCCCCOC(=O)\C=C\C1=CC=C(OC)C=C1	*L. panamensis*	AA	ND	248.562	(Otero et al., 2014a)
**390**	S	CCCCCOC(=O)CCC1=CC(OC)=C(OC)C=C1	*L. panamensis*	AA	ND	356.929	(Otero et al., 2014a)
**391**	S	CCCCCCCCCCCCCCCCOC(=O)\C=C\C1=CC=CC=C1	*L. panamensis*	AA	ND	268.599	(Otero et al., 2014a)
**392**	S	CCCCCCCCOC(=O)\C=C\C1=CC(O)=C(O)C=C1	*L. panamensis*	AA	High	7.872	(Otero et al., 2014a)
**393**	S	COC1=CC=CC(=C1OC)C1=CC=C(C=C1)C(=O)\C=C\C1=CC=CO1	*L. panamensis*	IA	Low	52.84	(Ochoa et al., 2019)
**394**	S	COC1=CC(OC)=C(C=C1)C1=CC=C(C=C1)C(=O)\C=C\C1=CC=CO1	*L. panamensis*	IA	High	9.36	(Ochoa et al., 2019)
**395**	S	COC1=CC(=C(OC)C=C1)C1=CC=C(C=C1)C(=O)\C=C\C1=CC=CO1	*L. panamensis*	IA	Intermediate	15.12	(Ochoa et al., 2019)
**396**	S	COC1=CC=CC(OC)=C1C1=CC=C(C=C1)C(=O)\C=C\C1=CC=CO1	*L. panamensis*	IA	Intermediate	13.44	(Ochoa et al., 2019)
**397**	S	COC1=C(OC)C=C(C=C1)C1=CC=C(C=C1)C(=O)\C=C\C1=CC=CO1	*L. panamensis*	IA	Intermediate	23.38	(Ochoa et al., 2019)
**398**	S	O=C(\C=C\C1=CC=CO1)C1=CC=C(C=C1)C1=CC=CC=C1	*L. panamensis*	IA	Low	180.61	(Ochoa et al., 2019)
**399**	S	FC1=CC=C(C=C1)C1=CC=C(C=C1)C(=O)\C=C\C1=CC=CO1	*L. panamensis*	IA	Low	719.61	(Ochoa et al., 2019)
**400**	S	COC1=CC=CC(=C1OC)C1=C(O)C=CC(=C1)C(=O)\C=C\C1=CC=CO1	*L. panamensis*	IA	High	6.53	(Ochoa et al., 2019)
**401**	S	COC1=CC(OC)=C(C=C1)C1=C(O)C=CC(=C1)C(=O)\C=C\C1=CC=C<br>O1	*L. panamensis*	IA	High	8.87	(Ochoa et al., 2019)
**402**	S	COC1=CC(=C(OC)C=C1)C1=C(O)C=CC(=C1)C(=O)\C=C\C1=CC=C<br>O1	*L. panamensis*	IA	Intermediate	19.15	(Ochoa et al., 2019)
**403**	S	COC1=C(OC)C=C(C=C1)C1=C(O)C=CC(=C1)C(=O)\C=C\C1=CC=C<br>O1	*L. panamensis*	IA	High	4.87	(Ochoa et al., 2019)
**404**	S	OC1=C(C=C(C=C1)C(=O)\C=C\C1=CC=CO1)C1=CC=CC=C1	*L. panamensis*	IA	Intermediate	24.49	(Ochoa et al., 2019)
**405**	S	OC1=C(C=C(C=C1)C(=O)\C=C\C1=CC=CO1)C1=CC=C(F)C=C1	*L. panamensis*	IA	High	6.09	(Ochoa et al., 2019)
**406**	S	CC1=CC=C2C=CC=C(OCCCOC3=C4N=C(C)C=CC4=CC=C3)C2=N1	*L. panamensis*	AA	Intermediate	23.732	(Cardona et al., 2013)
**407**	S	CC1=CC=C2C=CC=C(OCCCCOC3=C4N=C(C)C=CC4=CC=C3)C2=N1	*L. panamensis*	AA	ND	268.684	(Cardona et al., 2013)
**408**	S	CC1=CC=C2C=CC=C(OCCCCCOC3=C4N=C(C)C=CC4=CC=C3)C2=<br>N1	*L. panamensis*	AA	Intermediate	16.054	(Cardona et al., 2013)
**409**	S	CC1=CC=C2C=CC=C(OCCCCCCCCOC3=C4N=C(C)C=CC4=CC=C3)<br>C2=N1	*L. panamensis*	AA	ND	233.51	(Cardona et al., 2013)
**410**	S	CC1=CC=C2C=CC=C(OCCCCCCCCCOC3=C4N=C(C)C=CC4=CC=C3<br>)C2=N1	*L. panamensis*	AA	Low	25.55	(Cardona et al., 2013)
**411**	S	CC1=CC=C2C=CC=C(OCCCCCCCCCCCCOC3=C4N=C(C)C=CC4=C<br>C=C3)C2=N1	*L. panamensis*	AA	ND	206.48	(Cardona et al., 2013)

Comp[a]	Ori[b]	SMILES[c]	*Leishmania* species[d]	Parasite form[e]	Leishmanicidal Potential[f]	EC_{50} (μM)[g]	Reference
**412**	S	COC1=C(OCCCOC2=C3N=C(C)C=CC3=CC=C2)C=CC(CC=C)=C1	*L. panamensis*	AA	Intermediate	18.999	(Arango et al., 2012)
**413**	S	COC1=C(OCCCCOC2=C3N=C(C)C=CC3=CC=C2)C=CC(CC=C)=C1	*L. panamensis*	AA	Low	103.129	(Arango et al., 2012)
**414**	S	COC1=C(OCCCCCOC2=C3N=C(C)C=CC3=CC=C2)C=CC(CC=C)=C1	*L. panamensis*	AA	Intermediate	24.283	(Arango et al., 2012)
**415**	S	COC1=C(OCCCCCCCCOC2=C3N=C(C)C=CC3=CC=C2)C=CC(CC=C)=C1	*L. panamensis*	AA	Low	68.319	(Arango et al., 2012)
**416**	S	COC1=C(OCCCCCCCCCOC2=C3N=C(C)C=CC3=CC=C2)C=CC(CC=C)=C1	*L. panamensis*	AA	Low	47.174	(Arango et al., 2012)
**417**	S	COC1=C(OCCCCCCCCCCOC2=C3N=C(C)C=CC3=CC=C2)C=CC(CC=C)=C1	*L. panamensis*	AA	ND	216.782	(Arango et al., 2012)
**418**	S	CC1=NC2=C(OCCCOC3=C(OC4=CC=C(Cl)C=C4Cl)C=CC(Cl)=C3)C=CC=C2C=C1	*L. panamensis*	AA	Low	48.455	(Arango et al., 2012)
**419**	S	CC1=NC2=C(OCCCCOC3=C(OC4=CC=C(Cl)C=C4Cl)C=CC(Cl)=C3)C=CC=C2C=C1	*L. panamensis*	AA	Intermediate	19.359	(Arango et al., 2012)
**420**	S	CC1=NC2=C(OCCCCCOC3=C(OC4=CC=C(Cl)C=C4Cl)C=CC(Cl)=C3)C=CC=C2C=C1	*L. panamensis*	AA	High	7.96	(Arango et al., 2012)
**421**	S	CC1=NC2=C(OCCCCCCCCOC3=C(OC4=CC=C(Cl)C=C4Cl)C=CC(Cl)=C3)C=CC=C2C=C1	*L. panamensis*	AA	ND	179.492	(Arango et al., 2012)
**422**	S	CC1=NC2=C(OCCCCCCCCCOC3=C(OC4=CC=C(Cl)C=C4Cl)C=CC(Cl)=C3)C=CC=C2C=C1	*L. panamensis*	AA	ND	175.087	(Arango et al., 2012)
**423**	S	CC1=NC2=C(OCCCCCCCCCCCCOC3=C(OC4=CC=C(Cl)C=C4Cl)C=CC(Cl)=C3)C=CC=C2C=C1	*L. panamensis*	AA	ND	163.081	(Arango et al., 2012)
**424**	S	C1C2CC3=C(C=CC=C3)N(O2)C1C1=CC=CS1	*L. infantum*	P	Intermediate	15.9	(Blanco et al., 2014)
**425**	S	BrC1=CC=C(S1)C1CC2CC3=C(C=CC=C3)N1O2	*L. infantum*	P	Low	36.1	(Blanco et al., 2014)
**426**	S	ClC1=CC2=C(C=C1)N1OC(CC1C1=CC=CS1)C2	*L. infantum*	P	Intermediate	20.7	(Blanco et al., 2014)
**427**	S	CC1=CC=C(S1)C1CC2CC3=C(C=CC(Cl)=C3)N1O2	*L. infantum*	P	Low	32.3	(Blanco et al., 2014)
**428**	S	CC1=C(SC=C1)C1CC2CC3=C(C=CC(Cl)=C3)N1O2	*L. infantum*	P	Low	32.3	(Blanco et al., 2014)
**429**	S	ClC1=CC2=C(C=C1)N1OC(CC1C1=CC=C(Br)S1)C2	*L. infantum*	P	Intermediate	14.3	(Blanco et al., 2014)
**430**	S	FC1=CC2=C(C=C1)N1OC(CC1C1=CC=CS1)C2	*L. infantum*	P	Intermediate	11.5	(Blanco et al., 2014)
**431**	S	FC1=CC2=C(C=C1)N1OC(CC1C1=CC=C(Br)S1)C2	*L. infantum*	P	High	7.3	(Blanco et al., 2014)
**432**	S	CC1=CC=C(S1)C1CC2CC3=C(C=CC(OC(F)(F)F)=C3)N1O2	*L. infantum*	P	Intermediate	10.5	(Blanco et al., 2014)
**433**	S	CC1=C(SC=C1)C1CC2CC3=C(C=CC(OC(F)(F)F)=C3)N1O2	*L. infantum*	P	Intermediate	11.4	(Blanco et al., 2014)
**434**	S	C1C2CC3=C(C=CC=C3)N(O2)C1C1=CC=CO1	*L. infantum*	P	Low	45.3	(Blanco et al., 2014)
**435**	S	CC1=CC=C(O1)C1CC2CC3=C(C=CC=C3)N1O2	*L. infantum*	P	Low	27.3	(Blanco et al., 2014)
**436**	S	ClC1=CC2=C(C=C1)N1OC(CC1C1=CC=CO1)C2	*L. infantum*	P	Low	47.3	(Blanco et al., 2014)
**437**	S	CC1=CC=C(O1)C1CC2CC3=C(C=CC(Cl)=C3)N1O2	*L. infantum*	P	Intermediate	19.1	(Blanco et al., 2014)
**438**	S	[O-][N+](=O)C1=CC=C(O1)C1CC2CC3=C(C=CC(F)=C3)N1O2	*L. infantum*	P	Intermediate	10.7	(Blanco et al., 2014)
**439**	S	FC(F)(F)OC1=CC2=C(C=C1)N1OC(CC1C1=CC=CO1)C2	*L. infantum*	P	Intermediate	16	(Blanco et al., 2014)
**440**	S	CC1=CC=C(O1)C1CC2CC3=C(C=CC(OC(F)(F)F)=C3)N1O2	*L. infantum*	P	Intermediate	10.8	(Blanco et al., 2014)

Comp[a]	Ori[b]	SMILES[c]	*Leishmania* species[d]	Parasite form[e]	Leishmanicidal Potential[f]	EC_{50} (μM)[g]	Reference
**441**	S	FC(F)(F)OC1=CC2=C(C=C1)N1OC(CC1C1=COC=C1)C2	*L. infantum*	P	Low	25.2	(Blanco et al., 2014)
**442**	S	O[C@H]1C[C@H](NC2=C(C1)C=C(Cl)C=C2)C1=CC=CS1	*L. infantum*	P	Low	49.5	(Blanco et al., 2014)
**443**	S	CC1=CC=C(S1)[C@@H]1C[C@H](O)CC2=C(N1)C=CC(F)=C2	*L. infantum*	P	Intermediate	16.9	(Blanco et al., 2014)
**444**	S	O[C@H]1C[C@H](NC2=C(C1)C=C(OC(F)(F)F)C=C2)C1=CC=CS1	*L. infantum*	P	Intermediate	13.8	(Blanco et al., 2014)
**445**	S	CC1=CC=C(S1)[C@@H]1C[C@H](O)CC2=C(N1)C=CC(OC(F)(F)F)=C2	*L. infantum*	P	Intermediate	11.3	(Blanco et al., 2014)
**446**	S	CC1=C(SC=C1)[C@@H]1C[C@H](O)CC2=C(N1)C=CC(OC(F)(F)F)=C2	*L. infantum*	P	Intermediate	16.4	(Blanco et al., 2014)
**447**	S	O[C@H]1C[C@H](NC2=C(C1)C=C(OC(F)(F)F)C=C2)C1=CC=CO1	*L. infantum*	P	Low	45.9	(Blanco et al., 2014)
**448**	S	CC1=CC=C(O1)[C@@H]1C[C@H](O)CC2=C(N1)C=CC(OC(F)(F)F)=C2	*L. infantum*	P	Intermediate	12.8	(Blanco et al., 2014)
**449**	S	O[C@H]1C[C@H](NC2=C(C1)C=C(OC(F)(F)F)C=C2)C1=COC=C1	*L. infantum*	P	Low	43.1	(Blanco et al., 2014)
**450**	S	[I-].C[N+](C)(CCl)CCC=C(C1=CC=CC=C1)C1=CC=CC=C1	*L. panamensis*	IA	Low	89.918	(Duque-Benítez et al., 2016)
**451**	S	[I-].C[N+](C)(CCl)CCCC=C(C1=CC=CC=C1)C1=CC=CC=C1	*L. panamensis*	IA	Low	106.105	(Duque-Benítez et al., 2016)
**452**	S	[I-].C[N+](C)(CCl)CCCCC=C(C1=CC=CC=C1)C1=CC=CC=C1	*L. panamensis*	IA	Low	108.551	(Duque-Benítez et al., 2016)
**453**	S	[I-].C[N+](C)(CI)CCC=C(C1=CC=CC=C1)C1=CC=CC=C1	*L. panamensis*	IA	Low	53.565	(Duque-Benítez et al., 2016)
**454**	S	[I-].C[N+](C)(CI)CCCC=C(C1=CC=CC=C1)C1=CC=CC=C1	*L. panamensis*	IA	Low	45.027	(Duque-Benítez et al., 2016)
**455**	S	[I-].C[N+](C)(CI)CCCCC=C(C1=CC=CC=C1)C1=CC=CC=C1	*L. panamensis*	IA	Low	32.174	(Duque-Benítez et al., 2016)
**456**	S	[I-].C[N+](C)(C)CCC=C(C1=CC=CC=C1)C1=CC=CC=C1	*L. panamensis*	IA	Low	76.317	(Duque-Benítez et al., 2016)
**457**	S	[I-].C[N+](C)(C)CCCC=C(C1=CC=CC=C1)C1=CC=CC=C1	*L. panamensis*	IA	Low	60.671	(Duque-Benítez et al., 2016)
**458**	S	[I-].C[N+](C)(C)CCCCC=C(C1=CC=CC=C1)C1=CC=CC=C1	*L. panamensis*	IA	Low	98.783	(Duque-Benítez et al., 2016)
**459**	S	[I-].C[N+](C)(CCl)CCO	*L. panamensis*	IA	Low	111.709	(Duque-Benítez et al., 2016)
**460**	S	[I-].C[N+](C)(CI)CCO	*L. panamensis*	IA	Low	66.964	(Duque-Benítez et al., 2016)
**461**	S	[I-].C[N+](C)(C)CCO	*L. panamensis*	IA	Low	647.152	(Duque-Benítez et al., 2016)
**462**	S	C[C@@H]1[C@@H](C2=CC=C(O)C(OC)=C2)C3=CC=CC=C3NC14C(C=CC=C5)=C5NC4=O	*L. braziliensis*	IA	N/A		(Leañez et al., 2019)
**462**	S	C[C@@H]1[C@@H](C2=CC=C(O)C(OC)=C2)C3=CC=CC=C3NC14C(C=CC=C5)=C5NC4=O	*L. braziliensis*	P	N/A		(Leañez et al., 2019)
**463**	S	CC1=C2C([C@H](C3=CC=C(O)C(OC)=C3)[C@@H](C)C4(C(C=CC=C5)=C5NC4=O)N2)=CC=C1	*L. braziliensis*	IA	N/A		(Leañez et al., 2019)

Comp[a]	Ori[b]	SMILES[c]	*Leishmania* species[d]	Parasite form[e]	Leishmanicidal Potential[f]	EC_{50} (μM)[g]	Reference
**463**	S	CC1=C2C([C@H](C3=CC=C(O)C(OC)=C3)[C@@H](C)C4(C(C=CC=C5)=C5NC4=O)N2)=CC=C1	*L. braziliensis*	P	N/A		(Leañez et al., 2019)
**464**	S	C[C@@H]1[C@@H](C2=CC=C(O)C(OC)=C2)C3=CC(C)=CC=C3NC14C(C=CC=C5)=C5NC4=O	*L. braziliensis*	IA	N/A		(Leañez et al., 2019)
**464**	S	C[C@@H]1[C@@H](C2=CC=C(O)C(OC)=C2)C3=CC(C)=CC=C3NC14C(C=CC=C5)=C5NC4=O	*L. braziliensis*	P	N/A		(Leañez et al., 2019)
**465**	S	CCC1=C2C([C@H](C3=CC=C(O)C(OC)=C3)[C@@H](C)C4(C(C=CC=C5)=C5NC4=O)N2)=CC=C1	*L. braziliensis*	IA	High	3.3	(Leañez et al., 2019)
**465**	S	CCC1=C2C([C@H](C3=CC=C(O)C(OC)=C3)[C@@H](C)C4(C(C=CC=C5)=C5NC4=O)N2)=CC=C1	*L. braziliensis*	P	High	6	(Leañez et al., 2019)
**466**	S	C[C@@H]1[C@@H](C2=CC=C(O)C(OC)=C2)C3=CC(CC)=CC=C3NC14C(C=CC=C5)=C5NC4=O	*L. braziliensis*	IA	N/A		(Leañez et al., 2019)
**466**	S	C[C@@H]1[C@@H](C2=CC=C(O)C(OC)=C2)C3=CC(CC)=CC=C3NC14C(C=CC=C5)=C5NC4=O	*L. braziliensis*	P	N/A		(Leañez et al., 2019)
**467**	S	C[C@@H]1[C@@H](C2=CC=C(O)C(OC)=C2)C3=CC=CC(C#N)=C3NC14C(C=CC=C5)=C5NC4=O	*L. braziliensis*	IA	N/A		(Leañez et al., 2019)
**467**	S	C[C@@H]1[C@@H](C2=CC=C(O)C(OC)=C2)C3=CC=CC(C#N)=C3NC14C(C=CC=C5)=C5NC4=O	*L. braziliensis*	P	N/A		(Leañez et al., 2019)
**468**	S	C[C@@H]1[C@@H](C2=CC=C(O)C(OC)=C2)C3=CC(Cl)=CC=C3NC14C(C=CC=C5)=C5NC4=O	*L. braziliensis*	IA	N/A		(Leañez et al., 2019)
**468**	S	C[C@@H]1[C@@H](C2=CC=C(O)C(OC)=C2)C3=CC(Cl)=CC=C3NC14C(C=CC=C5)=C5NC4=O	*L. braziliensis*	P	N/A		(Leañez et al., 2019)
**469**	S	C[C@@H]1[C@@H](C2=CC=C(O)C(OC)=C2)C3=CC(Br)=CC=C3NC14C(C=CC=C5)=C5NC4=O	*L. braziliensis*	IA	N/A		(Leañez et al., 2019)
**469**	S	C[C@@H]1[C@@H](C2=CC=C(O)C(OC)=C2)C3=CC(Br)=CC=C3NC14C(C=CC=C5)=C5NC4=O	*L. braziliensis*	P	N/A		(Leañez et al., 2019)
**470**	S	O=C1NC2=C(C=CC=C2)C13N(C4=CC=CC=C4)C(CS3)=O	*L. braziliensis*	IA	N/A		(Leañez et al., 2019)
**470**	S	O=C1NC2=C(C=CC=C2)C13N(C4=CC=CC=C4)C(CS3)=O	*L. braziliensis*	P	N/A		(Leañez et al., 2019)
**471**	S	O=C1NC2=C(C=CC=C2)C13OC(CS3)=O	*L. braziliensis*	IA	N/A		(Leañez et al., 2019)
**471**	S	O=C1NC2=C(C=CC=C2)C13OC(CS3)=O	*L. braziliensis*	P	ND	50	(Leañez et al., 2019)
**472**	S	C12=CC=CC=C1C=CC(C3=CC=C(OCO4)C4=C3)=N2	*L. braziliensis*	IA	N/A		(Bompart et al., 2013)
**472**	S	C12=CC=CC=C1C=CC(C3=CC=C(OCO4)C4=C3)=N2	*L. braziliensis*	P	ND	50	(Bompart et al., 2013)
**473**	S	CC1=CC=C(N=C(C2=CC=C(OCO3)C3=C2)C=C4)C4=C1	*L. braziliensis*	IA	N/A		(Bompart et al., 2013)
**473**	S	CC1=CC=C(N=C(C2=CC=C(OCO3)C3=C2)C=C4)C4=C1	*L. braziliensis*	P	ND	50	(Bompart et al., 2013)
**474**	S	CCC1=CC=C(N=C(C2=CC=C(OCO3)C3=C2)C=C4)C4=C1	*L. braziliensis*	IA	N/A		(Bompart et al., 2013)
**474**	S	CCC1=CC=C(N=C(C2=CC=C(OCO3)C3=C2)C=C4)C4=C1	*L. braziliensis*	P	ND	50	(Bompart et al., 2013)
**475**	S	COC1=CC=C(N=C(C2=CC=C(OCO3)C3=C2)C=C4)C4=C1	*L. braziliensis*	IA	N/A		(Bompart et al., 2013)
**475**	S	COC1=CC=C(N=C(C2=CC=C(OCO3)C3=C2)C=C4)C4=C1	*L. braziliensis*	P	ND	50	(Bompart et al., 2013)
**476**	S	CC1=C2C(N=C(C3=CC=C(OCO4)C4=C3)C=C2)=CC(C)=C1	*L. braziliensis*	IA	N/A		(Bompart et al., 2013)

Comp[a]	Ori[b]	SMILES[c]	*Leishmania* species[d]	Parasite form[e]	Leishmanicidal Potential[f]	EC_{50} (μM)[g]	Reference
**476**	S	CC1=C2C(N=C(C3=CC=C(OCO4)C4=C3)C=C2)=CC(C)=C1	*L. braziliensis*	P	ND	50	(Bompart et al., 2013)
**477**	S	CCC1=CC=C(N=C(C2=CC=CC=C2)C=C3)C3=C1	*L. braziliensis*	IA	Intermediate	20	(Bompart et al., 2013)
**477**	S	CCC1=CC=C(N=C(C2=CC=CC=C2)C=C3)C3=C1	*L. braziliensis*	P	High	6	(Bompart et al., 2013)
**478**	S	CC1=C2C(N=C(C3=CC=CC=C3)C=C2)=CC(C)=C1	*L. braziliensis*	IA	N/A		(Bompart et al., 2013)
**478**	S	CC1=C2C(N=C(C3=CC=CC=C3)C=C2)=CC(C)=C1	*L. braziliensis*	P	N/A		(Bompart et al., 2013)
**479**	S	ClC1=CC=C2C(NC3=CC=CC(C=O)=C3)=CC=NC2=C1	*L. panamensis*	IA	High	3.404	(Ramírez–Prada et al., 2017)
**480**	S	ClC1=CC=C(C=C1)C(=O)\C=C\C1=CC(NC2=CC=NC3=CC(Cl)=CC=C23)=CC=C1	*L. panamensis*	IA	High	1.89	(Ramírez–Prada et al., 2017)
**481**	S	ClC1=CC=C2C(NC3=CC=CC(\C=C\C(=O)C4=CC=C(Br)C=C4)=C3)=CC=NC2=C1	*L. panamensis*	IA	High	1.905	(Ramírez–Prada et al., 2017)
**482**	S	COC1=CC=C(C=C1)C(=O)\C=C\C1=CC(NC2=CC=NC3=CC(Cl)=CC=C23)=CC=C1	*L. panamensis*	IA	High	6.399	(Ramírez–Prada et al., 2017)
**483**	S	COC1=CC(=CC(OC)=C1OC)C(=O)\C=C\C1=CC(NC2=CC=NC3=CC(Cl)=CC=C23)=CC=C1	*L. panamensis*	IA	Intermediate	12.486	(Ramírez–Prada et al., 2017)
**484**	S	CC1=CC=C(C=C1)C(=O)\C=C\C1=CC(NC2=CC=NC3=CC(Cl)=CC=C23)=CC=C1	*L. panamensis*	IA	High	3.014	(Ramírez–Prada et al., 2017)
**485**	S	ClC1=CC=C2C(NC3=CC=CC(\C=C\C(=O)C4=CC=CC=C4)=C3)=CC=NC2=C1	*L. panamensis*	IA	High	6.613	(Ramírez–Prada et al., 2017)
**486**	S	CC(=O)N1N=C(CC1C1=CC(NC2=CC=NC3=CC(Cl)=CC=C23)=CC=C1)C1=CC=C(Cl)C=C1	*L. panamensis*	IA	Intermediate	11.664	(Ramírez–Prada et al., 2017)
**487**	S	CC(=O)N1N=C(CC1C1=CC(NC2=CC=NC3=CC(Cl)=CC=C23)=CC=C1)C1=CC=C(Br)C=C1	*L. panamensis*	IA	High	7.065	(Ramírez–Prada et al., 2017)
**488**	S	COC1=CC=C(C=C1)C1=NN(C(C1)C1=CC(NC2=CC=NC3=CC(Cl)=CC=C23)=CC=C1)C(C)=O	*L. panamensis*	IA	High	6.338	(Ramírez–Prada et al., 2017)
**489**	S	COC1=CC(=CC(OC)=C1OC)C1=NN(C(C1)C1=CC(NC2=CC=NC3=CC(Cl)=CC=C23)=CC=C1)C(C)=O	*L. panamensis*	IA	High	3.697	(Ramírez–Prada et al., 2017)
**490**	S	CC(=O)N1N=C(CC1C1=CC(NC2=CC=NC3=CC(Cl)=CC=C23)=CC=C1)C1=CC=C(C)C=C1	*L. panamensis*	IA	High	3.413	(Ramírez–Prada et al., 2017)
**491**	S	CC(=O)N1N=C(CC1C1=CC(NC2=CC=NC3=CC(Cl)=CC=C23)=CC=C1)C1=CC=CC=C1	*L. panamensis*	IA	High	8.338	(Ramírez–Prada et al., 2017)
**492**	S	ClC1=CC=C(C=C1)C1=NN(C=O)C(C1)C1=CC(NC2=CC=NC3=CC(Cl)=CC=C23)=CC=C1	*L. panamensis*	IA	Low	610.582	(Ramírez–Prada et al., 2017)
**493**	S	ClC1=CC=C2C(NC3=CC=CC(=C3)C3CC(=NN3C=O)C3=CC=C(Br)C=C3)=CC=NC2=C1	*L. panamensis*	IA	High	4.98	(Ramírez–Prada et al., 2017)
**494**	S	COC1=CC=C(C=C1)C1=NN(C=O)C(C1)C1=CC(NC2=CC=NC3=CC(Cl)=CC=C23)=CC=C1	*L. panamensis*	IA	Low	72.654	(Ramírez–Prada et al., 2017)
**495**	S	COC1=CC(=CC(OC)=C1OC)C1=NN(C=O)C(C1)C1=CC(NC2=CC=NC3=CC(Cl)=CC=C23)=CC=C1	*L. panamensis*	IA	Intermediate	13.155	(Ramírez–Prada et al., 2017)

Comp[a]	Ori[b]	SMILES[c]	*Leishmania* species[d]	Parasite form[e]	Leishmanicidal Potential[f]	EC_{50} (μM)[g]	Reference
**496**	S	CC1=CC=C(C=C1)C1=NN(C=O)C(C1)C1=CC(NC2=CC=NC3=CC(Cl)=CC=C23)=CC=C1	*L. panamensis*	IA	High	4.976	(Ramírez–Prada et al., 2017)
**497**	S	ClC1=CC=C2C(NC3=CC=CC(=C3)C3CC(=NN3C=O)C3=CC=CC=C3)=CC=NC2=C1	*L. panamensis*	IA	High	4.036	(Ramírez–Prada et al., 2017)
**498**	S	ClC1=CC=C(C=C1)C1=NN(C(C1)C1=CC(NC2=CC=NC3=CC(Cl)=CC=C23)=CC=C1)C1=CC=CC=C1	*L. panamensis*	IA	Intermediate	23.006	(Ramírez–Prada et al., 2017)
**499**	S	ClC1=CC=C2C(NC3=CC=CC(=C3)C3CC(=NN3C3=CC=CC=C3)C3=CC=C(Br)C=C3)=CC=NC2=C1	*L. panamensis*	IA	High	6.503	(Ramírez–Prada et al., 2017)
**500**	S	COC1=CC=C(C=C1)C1=NN(C(C1)C1=CC(NC2=CC=NC3=CC(Cl)=CC=C23)=CC=C1)C1=CC=CC=C1	*L. panamensis*	IA	Intermediate	13.745	(Ramírez–Prada et al., 2017)
**501**	S	COC1=CC(=CC(OC)=C1OC)C1=NN(C(C1)C1=CC(NC2=CC=NC3=CC(Cl)=CC=C23)=CC=C1)C1=CC=CC=C1	*L. panamensis*	IA	Intermediate	23.768	(Ramírez–Prada et al., 2017)
**502**	S	CC1=CC=C(C=C1)C1=NN(C(C1)C1=CC(NC2=CC=NC3=CC(Cl)=CC=C23)=CC=C1)C1=CC=CC=C1	*L. panamensis*	IA	High	3.749	(Ramírez–Prada et al., 2017)
**503**	S	ClC1=CC=C2C(NC3=CC=CC(=C3)C3CC(=NN3C3=CC=CC=C3)C3=CC=CC=C3)=CC=NC2=C1	*L. panamensis*	IA	Low	47.811	(Ramírez–Prada et al., 2017)
**504**	S	ClC1=CC=C(C=C1)N1N=C(CC1C1=CC(NC2=CC=NC3=CC(Cl)=CC=C23)=CC=C1)C1=CC=C(Cl)C=C1	*L. panamensis*	IA	Low	168.59	(Ramírez–Prada et al., 2017)
**505**	S	ClC1=CC=C(C=C1)N1N=C(CC1C1=CC(NC2=CC=NC3=CC(Cl)=CC=C23)=CC=C1)C1=CC=C(Br)C=C1	*L. panamensis*	IA	Low	27.285	(Ramírez–Prada et al., 2017)
**506**	S	COC1=CC=C(C=C1)C1=NN(C(C1)C1=CC(NC2=CC=NC3=CC(Cl)=CC=C23)=CC=C1)C1=CC=C(Cl)C=C1	*L. panamensis*	IA	Low	51.53	(Ramírez–Prada et al., 2017)
**507**	S	COC1=CC(=CC(OC)=C1OC)C1=NN(C(C1)C1=CC(NC2=CC=NC3=CC(Cl)=CC=C23)=CC=C1)C1=CC=C(Cl)C=C1	*L. panamensis*	IA	Low	35.543	(Ramírez–Prada et al., 2017)
**508**	S	CC1=CC=C(C=C1)C1=NN(C(C1)C1=CC(NC2=CC=NC3=CC(Cl)=CC=C23)=CC=C1)C1=CC=C(Cl)C=C1	*L. panamensis*	IA	Low	94.611	(Ramírez–Prada et al., 2017)
**509**	S	ClC1=CC=C(C=C1)N1N=C(CC1C1=CC(NC2=CC=NC3=CC(Cl)=CC=C23)=CC=C1)C1=CC=CC=C1	*L. panamensis*	IA	Low	30.839	(Ramírez–Prada et al., 2017)
**510**	S	ClC1=CC=C(C=C1)C1=NN(C(C1)C1=CC(NC2=CC=NC3=CC(Cl)=CC=C23)=CC=C1)C1=CC(Cl)=CC(Cl)=C1	*L. panamensis*	IA	Intermediate	20.919	(Ramírez–Prada et al., 2017)
**511**	S	ClC1=CC=C2C(NC3=CC=CC(=C3)C3CC(=NN3C3=CC(Cl)=CC(Cl)=C3)C3=CC=C(Br)C=C3)=CC=NC2=C1	*L. panamensis*	IA	High	4.468	(Ramírez–Prada et al., 2017)
**512**	S	COC1=CC=C(C=C1)C1=NN(C(C1)C1=CC(NC2=CC=NC3=CC(Cl)=CC=C23)=CC=C1)C1=CC(Cl)=CC(Cl)=C1	*L. panamensis*	IA	Intermediate	22.042	(Ramírez–Prada et al., 2017)
**513**	S	COC1=CC(=CC(OC)=C1OC)C1=NN(C(C1)C1=CC(NC2=CC=NC3=CC(Cl)=CC=C23)=CC=C1)C1=CC(Cl)=CC(Cl)=C1	*L. panamensis*	IA	Low	26.087	(Ramírez–Prada et al., 2017)
**514**	S	CC1=CC=C(C=C1)C1=NN(C(C1)C1=CC(NC2=CC=NC3=CC(Cl)=CC=C23)=CC=C1)C1=CC(Cl)=CC(Cl)=C1	*L. panamensis*	IA	Low	32.584	(Ramírez–Prada et al., 2017)

Comp[a]	Ori[b]	SMILES[c]	*Leishmania* species[d]	Parasite form[e]	Leishmanicidal Potential[f]	EC_{50} (μM)[g]	Reference
**515**	S	ClC1=CC=C2C(NC3=CC=CC(=C3)C3CC(=NN3C3=CC(Cl)=CC(Cl)=C3)C3=CC=CC=C3)=CC=NC2=C1	*L. panamensis*	IA	Low	32.91	(Ramírez–Prada et al., 2017)
**516**	S	[H][C@]12C[C@]([H])(N(O1)C1=C(C2)C=C(Br)C2=C1C=CC=C2)C1=CC=CC=C1	*L. infantum*	P	Low	485.1	(Yépes et al., 2018)
**517**	S	[H][C@]12C[C@]([H])(N(O1)C1=C(C2)C=C(Br)C2=C1C=CC=C2)C1=C(C)C=CC=C1	*L. infantum*	P	ND	787.2	(Yépes et al., 2018)
**518**	S	[H][C@]12C[C@]([H])(N(O1)C1=C(C2)C=C(Br)C2=C1C=CC=C2)C1=CC(C)=CC=C1	*L. infantum*	P	Low	117.4	(Yépes et al., 2018)
**519**	S	[H][C@]12C[C@]([H])(N(O1)C1=C(C2)C=C(Br)C2=C1C=CC=C2)C1=CC(OC)=CC=C1	*L. infantum*	P	Low	40.1	(Yépes et al., 2018)
**520**	S	[H][C@]12C[C@]([H])(N(O1)C1=C(C2)C=C(Br)C2=C1C=CC=C2)C1=C(Cl)C=CC=C1	*L. infantum*	P	ND	751.9	(Yépes et al., 2018)
**521**	S	[H][C@]12C[C@]([H])(N(O1)C1=C(C2)C=C(Br)C2=C1C=CC=C2)C1=CC=C(Cl)C=C1	*L. infantum*	P	Low	116.4	(Yépes et al., 2018)
**522**	S	[H][C@]12C[C@]([H])(N(O1)C1=C(C2)C=C(Br)C2=C1C=CC=C2)C1=C(Cl)C=C(Cl)C=C1	*L. infantum*	P	Low	692.9	(Yépes et al., 2018)
**523**	S	[H][C@]12C[C@]([H])(N(O1)C1=C(C2)C=CC2=C1C=CC=C2)C1=C(C)C=CC=C1	*L. infantum*	P	Low	604.45	(Yépes et al., 2018)
**524**	S	[H][C@]12C[C@]([H])(N(O1)C1=C(C2)C=CC2=C1C=CC=C2)C1=CC(=CC=C1)[N+]([O-])=O	*L. infantum*	P	Low	98.6	(Yépes et al., 2018)
**525**	S	[H][C@]12C[C@]([H])(N(O1)C1=C(C2)C=CC2=C1C=CC=C2)C1=CC=C(C=C1)[N+]([O-])=O	*L. infantum*	P	High	5	(Yépes et al., 2018)
**526**	S	[H][C@]12C[C@]([H])(N(O1)C1=C(C2)C=C(Br)C2=C1C=CC=C2)C1=CC=CS1	*L. infantum*	P	Intermediate	20.1	(Yépes et al., 2018)
**527**	S	[H][C@]12C[C@]([H])(N(O1)C1=C(C2)C=C(Br)C2=C1C=CC=C2)C1=CC=C(C)S1	*L. infantum*	P	Low	29.3	(Yépes et al., 2018)
**528**	S	[H][C@]12C[C@]([H])(N(O1)C1=C(C2)C=CC2=C1C=CC=C2)C1=CC=CS1	*L. infantum*	P	Low	777.5	(Yépes et al., 2018)
**529**	S	[H][C@]12C[C@]([H])(N(O1)C1=C(C2)C=CC2=C1C=CC=C2)C1=CC=C(C)S1	*L. infantum*	P	Intermediate	10.9	(Yépes et al., 2018)
**530**	S	[H][C@]12C[C@]([H])(N(O1)C1=C(C2)C=CC2=C1C=CC=C2)C1=C(C)C=CS1	*L. infantum*	P	Low	590.4	(Yépes et al., 2018)
**531**	S	[H][C@]12C[C@]([H])(N(O1)C1=C(C2)C=CC2=C1C=CC=C2)C1=CC=C(Br)S1	*L. infantum*	P	Intermediate	21.6	(Yépes et al., 2018)
**532**	S	[H][C@]12C[C@]([H])(N(O1)C1=C(C2)C=C(Br)C2=C1C=CC=C2)C1=CC=CO1	*L. infantum*	P	Low	142.7	(Yépes et al., 2018)
**533**	S	[H][C@]12C[C@]([H])(N(O1)C1=C(C2)C=CC2=C1C=CC=C2)C1=CC=CO1	*L. infantum*	P	Low	60.3	(Yépes et al., 2018)

Comp[a]	Ori[b]	SMILES[c]	*Leishmania* species[d]	Parasite form[e]	Leishmanicidal Potential[f]	EC_{50} (μM)[g]	Reference
**534**	S	[H][C@]12C[C@]([H])(N(O1)C1=C(C2)C=CC2=C1C=CC=C2)C1=CC=C(C)O1	*L. infantum*	P	Intermediate	15.4	(Yépes et al., 2018)
**535**	S	[H][C@]12C[C@]([H])(N(O1)C1=C(C2)C=CC2=C1C=CC=C2)C1=CC=C(O1)[N+]([O-])=O	*L. infantum*	P	Intermediate	13.9	(Yépes et al., 2018)
**536**	S	[H][C@@]1(O)CC2=C(N[C@@]([H])(C1)C1=CC=CC=C1)C1=C(C=CC=C1)C(Br)=C2	*L. infantum*	P	Low	617.1	(Yépes et al., 2018)
**537**	S	[H][C@@]1(O)CC2=C(N[C@@]([H])(C1)C1=CC=CC=C1C)C1=C(C=CC=C1)C(Br)=C2	*L. infantum*	P	High	7.7	(Yépes et al., 2018)
**538**	S	[H][C@@]1(O)CC2=C(N[C@@]([H])(C1)C1=CC=CC(C)=C1)C1=C(C=CC=C1)C(Br)=C2	*L. infantum*	P	Intermediate	12.6	(Yépes et al., 2018)
**539**	S	[H][C@@]1(O)CC2=C(N[C@@]([H])(C1)C1=CC=CC(OC)=C1)C1=C(C=CC=C1)C(Br)=C2	*L. infantum*	P	High	9.6	(Yépes et al., 2018)
**540**	S	[H][C@@]1(O)CC2=C(N[C@@]([H])(C1)C1=CC=CC=C1Cl)C1=C(C=CC=C1)C(Br)=C2	*L. infantum*	P	High	8.9	(Yépes et al., 2018)
**541**	S	[H][C@@]1(O)CC2=C(N[C@@]([H])(C1)C1=CC=C(Cl)C=C1)C1=C(C=CC=C1)C(Br)=C2	*L. infantum*	P	Low	48.2	(Yépes et al., 2018)
**542**	S	[H][C@@]1(O)CC2=C(N[C@@]([H])(C1)C1=CC=C(Cl)C=C1Cl)C1=C(C=CC=C1)C(Br)=C2	*L. infantum*	P	Intermediate	11.8	(Yépes et al., 2018)
**543**	S	[H][C@@]1(O)CC2=C(N[C@@]([H])(C1)C1=CC=CC=C1C)C1=C(C=CC=C1)C=C2	*L. infantum*	P	Low	36.19	(Yépes et al., 2018)
**544**	S	[H][C@@]1(O)CC2=C(N[C@@]([H])(C1)C1=CC=CS1)C1=C(C=CC=C1)C=C2	*L. infantum*	P	Low	42.5	(Yépes et al., 2018)
**545**	S	[H][C@@]1(O)CC2=C(N[C@@]([H])(C1)C1=CC=C(C)S1)C1=C(C=CC=C1)C=C2	*L. infantum*	P	Intermediate	16.6	(Yépes et al., 2018)
**546**	S	[H][C@@]1(O)CC2=C(N[C@@]([H])(C1)C1=C(C)C=CS1)C1=C(C=CC=C1)C=C2	*L. infantum*	P	Low	58.5	(Yépes et al., 2018)
**547**	S	C1=CC=C(C=C1)C1=C2N=CC=CC2=CC=C1	*L. panamensis*	IA	Low	43.64	(Coa et al., 2020)
**548**	S	OC1=CC=C(C=C1)C1=C2N=CC=CC2=CC=C1	*L. panamensis*	IA	Low	69.385	(Coa et al., 2020)
**549**	S	COC1=CC=C(C=C1)C1=C2N=CC=CC2=CC=C1	*L. panamensis*	IA	Low	143.046	(Coa et al., 2020)
**550**	S	OC1=C(C=CC=C1)C1=C2N=CC=CC2=CC=C1	*L. panamensis*	IA	Low	114.3	(Coa et al., 2020)
**551**	S	FC1=CC=C(C=C1)C1=C2N=CC=CC2=CC=C1	*L. panamensis*	IA	Low	76.251	(Coa et al., 2020)
**552**	S	[O-][N+](=O)C1=CC=C(C=C1)C1=C2N=CC=CC2=CC=C1	*L. panamensis*	IA	Low	55.424	(Coa et al., 2020)
**553**	S	COC1=CC=CC(=C1OC)C1=C2N=CC=CC2=CC=C1	*L. panamensis*	IA	Low	482.252	(Coa et al., 2020)
**554**	S	COC1=CC(OC)=C(C=C1)C1=C2N=CC=CC2=CC=C1	*L. panamensis*	IA	Low	99.808	(Coa et al., 2020)
**555**	S	COC1=CC(=C(OC)C=C1)C1=C2N=CC=CC2=CC=C1	*L. panamensis*	IA	Low	155.671	(Coa et al., 2020)
**556**	S	COC1=CC=CC(OC)=C1C1=C2N=CC=CC2=CC=C1	*L. panamensis*	IA	Low	380.823	(Coa et al., 2020)
**557**	S	COC1=C(OC)C=C(C=C1)C1=C2N=CC=CC2=CC=C1	*L. panamensis*	IA	Low	61.333	(Coa et al., 2020)
**558**	S	COC1=CC=C(\C=C\C(=O)C2=CC=C(OCCCOC3=CC(Cl)=CC=C3OC3=C(Cl)C=C(Cl)C=C3)C=C2)C=C1OC	*L. panamensis*	IA	Intermediate	15.357	(Otero et al., 2014b)

Comp[a]	Ori[b]	SMILES[c]	*Leishmania* species[d]	Parasite form[e]	Leishmanicidal Potential[f]	EC_{50} (μM)[g]	Reference
**559**	S	COC1=CC=C(\C=C\C(=O)C2=CC=C(OCCCCOC3=CC(Cl)=CC=C3OC3=C(Cl)C=C(Cl)C=C3)C=C2)C=C1OC	*L. panamensis*	IA	Intermediate	16.291	(Otero et al., 2014b)
**560**	S	COC1=CC=C(\C=C\C(=O)C2=CC=C(OCCCCCOC3=CC(Cl)=CC=C3OC3=C(Cl)C=C(Cl)C=C3)C=C2)C=C1OC	*L. panamensis*	IA	Intermediate	21.09	(Otero et al., 2014b)
**561**	S	COC1=CC=C(\C=C\C(=O)C2=CC=C(OCCCCCCCCOC3=CC(Cl)=CC=C3OC3=C(Cl)C=C(Cl)C=C3)C=C2)C=C1OC	*L. panamensis*	IA	ND	29.318	(Otero et al., 2014b)
**562**	S	CC1=CC(=O)OC2=C1C=CC(OCCCOC1=CC(Cl)=CC=C1OC1=C(Cl)C=C(Cl)C=C1)=C2	*L. panamensis*	IA	ND	39.68	(Otero et al., 2014b)
**563**	S	CC1=CC(=O)OC2=C1C=CC(OCCCCOC1=CC(Cl)=CC=C1OC1=C(Cl)C=C(Cl)C=C1)=C2	*L. panamensis*	IA	Low	53.084	(Otero et al., 2014b)
**564**	S	CC1=CC(=O)OC2=C1C=CC(OCCCCCOC1=CC(Cl)=CC=C1OC1=C(Cl)C=C(Cl)C=C1)=C2	*L. panamensis*	IA	ND	37.59	(Otero et al., 2014b)
**565**	S	CC1=CC(=O)OC2=C1C=CC(OCCCCCCCCOC1=CC(Cl)=CC=C1OC1=C(Cl)C=C(Cl)C=C1)=C2	*L. panamensis*	IA	ND	34.837	(Otero et al., 2014b)
**566**	S	ClC1=CC(Cl)=C(OC2=CC=C(Cl)C=C2OCCCOC2=CC3=C(C=C2)C(=O)C=CO3)C=C1	*L. panamensis*	IA	High	5.51	(Otero et al., 2014b)
**567**	S	ClC1=CC(Cl)=C(OC2=CC=C(Cl)C=C2OCCCCOC2=CC3=C(C=C2)C(=O)C=CO3)C=C1	*L. panamensis*	IA	Intermediate	14.88	(Otero et al., 2014b)
**568**	S	ClC1=CC(Cl)=C(OC2=CC=C(Cl)C=C2OCCCCCOC2=CC3=C(C=C2)C(=O)C=CO3)C=C1	*L. panamensis*	IA	Low	30.885	(Otero et al., 2014b)
**569**	S	ClC1=CC(Cl)=C(OC2=CC=C(Cl)C=C2OCCCCCCCCOC2=CC3=C(C=C2)C(=O)C=CO3)C=C1	*L. panamensis*	IA	ND	35.708	(Otero et al., 2014b)
**570**	S	COC1=C(OC)C=C(\C=C\C(=O)C2=CC=C(OCCCOC3=CC=CC4=CC=CN=C34)C=C2)C=C1	*L. panamensis*	IA	Low	25.128	(Coa et al., 2017)
**571**	S	COC1=C(OC)C=C(\C=C\C(=O)C2=CC=C(OCCCCOC3=CC=CC4=CC=CN=C34)C=C2)C=C1	*L. panamensis*	IA	Intermediate	12.914	(Coa et al., 2017)
**572**	S	COC1=C(OC)C=C(\C=C\C(=O)C2=CC=C(OCCCCCOC3=CC=CC4=CC=CN=C34)C=C2)C=C1	*L. panamensis*	IA	Intermediate	24.878	(Coa et al., 2017)
**573**	S	COC1=C(OC)C=C(\C=C\C(=O)C2=CC=C(OCCCCCCCCOC3=CC=CC4=CC=CN=C34)C=C2)C=C1	*L. panamensis*	IA	Intermediate	15.818	(Coa et al., 2017)
**574**	S	COC1=C(OC)C=C(\C=C\C(=O)C2=CC=C(OCCCCCCCCCOC3=CC=CC4=CC=CN=C34)C=C2)C=C1	*L. panamensis*	IA	Low	29.659	(Coa et al., 2017)
**575**	S	COC1=C(OC)C=C(\C=C\C(=O)C2=CC=C(OCCCCCCCCCCCCOC3=CC=CC4=CC=CN=C34)C=C2)C=C1	*L. panamensis*	IA	Low	36.954	(Coa et al., 2017)
**576**	S	O=C1C=COC2=C1C=CC(OCCCCOC1=CC=CC3=C1N=CC=C3)=C2	*L. panamensis*	IA	Intermediate	16.919	(Coa et al., 2017)
**577**	S	O=C1C=COC2=C1C=CC(OCCCCCOC1=CC=CC3=C1N=CC=C3)=C2	*L. panamensis*	IA	Low	128.723	(Coa et al., 2017)
**578**	S	O=C1C=COC2=C1C=CC(OCCCCCCCCOC1=CC=CC3=C1N=CC=C3)=C2	*L. panamensis*	IA	Low	51.631	(Coa et al., 2017)

Comp[a]	Ori[b]	SMILES[c]	*Leishmania* species[d]	Parasite form[e]	Leishmanicidal Potential[f]	EC_{50} (μM)[g]	Reference
**579**	S	O=C1C=COC2=C1C=CC(OCCCCCCCCCOC1=CC=CC3=C1N=CC=C3)=C2	*L. panamensis*	IA	Intermediate	17.045	(Coa et al., 2017)
**580**	S	O=C1C=COC2=C1C=CC(OCCCCCCCCCCCCOC1=CC=CC3=C1N=CC=C3)=C2	*L. panamensis*	IA	Low	34.189	(Coa et al., 2017)
**581**	S	OC1=C(\C=N\NC(=O)C2=NC3=CC=CC=C3C=C2)C=CC=C1	*L. panamensis*	IA	ND	68.705	(Coa et al., 2015)
**582**	S	OC1=CC=CC(\C=N\NC(=O)C2=NC3=CC=CC=C3C=C2)=C1O	*L. panamensis*	IA	Intermediate	21.166	(Coa et al., 2015)
**583**	S	OC1=CC(O)=C(\C=N\NC(=O)C2=NC3=CC=CC=C3C=C2)C=C1	*L. panamensis*	IA	High	2.605	(Coa et al., 2015)
**584**	S	OC1=CC(\C=N\NC(=O)C2=NC3=CC=CC=C3C=C2)=C(O)C=C1	*L. panamensis*	IA	Low	121.135	(Coa et al., 2015)
**585**	S	OC1=C(O)C(O)=C(\C=N\NC(=O)C2=NC3=CC=CC=C3C=C2)C=C1	*L. panamensis*	IA	Low	47.665	(Coa et al., 2015)
**586**	S	OC1=CC=CC(\C=N\NC(=O)C2=CC=NC3=CC=CC=C23)=C1O	*L. panamensis*	IA	ND	32.563	(Coa et al., 2015)
**587**	S	OC1=CC(O)=C(\C=N\NC(=O)C2=CC=NC3=CC=CC=C23)C=C1	*L. panamensis*	IA	ND	16.282	(Coa et al., 2015)
**588**	S	OC1=CC(\C=N\NC(=O)C2=CC=NC3=CC=CC=C23)=C(O)C=C1	*L. panamensis*	IA	ND	65.126	(Coa et al., 2015)
**589**	S	OC1=C(O)C(O)=C(\C=N\NC(=O)C2=CC=NC3=CC=CC=C23)C=C1	*L. panamensis*	IA	ND	61.902	(Coa et al., 2015)
**590**	S	OC1=CC=C(\C=N\NC(=O)C2=NC=CC3=CC=CC=C23)C(O)=C1	*L. panamensis*	IA	Intermediate	11.071	(Coa et al., 2015)
**591**	S	C1C2CC3=CC=CC=C3N(O2)[C@@H]1C1=CC=CC=C1	*L. chagasi*	IA	ND	100	(Gómez-Ayala et al., 2010)
**592**	S	FC(F)(F)OC1=CC=C2N3OC(C[C@H]3C3=CC=CC=C3)CC2=C1	*L. chagasi*	IA	ND	100	(Gómez-Ayala et al., 2010)
**593**	S	BrC1=CC=C2N3OC(C[C@H]3C3=CC=CC=C3)CC2=C1	*L. chagasi*	IA	ND	100	(Gómez-Ayala et al., 2010)
**594**	S	ClC1=CC=C2N3OC(C[C@H]3C3=CC=CC=C3)CC2=C1	*L. chagasi*	IA	ND	100	(Gómez-Ayala et al., 2010)
**595**	S	FC1=CC=C2N3OC(C[C@H]3C3=CC=CC=C3)CC2=C1	*L. chagasi*	IA	ND	100	(Gómez-Ayala et al., 2010)
**596**	S	ClC1=CC=C(C=C1)[C@@H]1CC2CC3=CC=CC=C3N1O2	*L. chagasi*	IA	ND	100	(Gómez-Ayala et al., 2010)
**597**	S	FC1=CC=C(C=C1)[C@@H]1CC2CC3=CC=CC=C3N1O2	*L. chagasi*	IA	ND	100	(Gómez-Ayala et al., 2010)
**598**	S	CCC1=CC(=CC=C1)[C@@H]1CC2CC3=CC=CC=C3N1O2	*L. chagasi*	IA	ND	100	(Gómez-Ayala et al., 2010)
**599**	S	CC1=CC(=CC=C1)[C@@H]1CC2CC3=CC=CC=C3N1O2	*L. chagasi*	IA	ND	100	(Gómez-Ayala et al., 2010)
**600**	S	ClC1=CC(=CC=C1)[C@@H]1CC2CC3=CC=CC=C3N1O2	*L. chagasi*	IA	ND	100	(Gómez-Ayala et al., 2010)
**601**	S	CC1=CC=C(C=C1)[C@@H]1CC2CC3=CC(C)=CC=C3N1O2	*L. chagasi*	IA	ND	100	(Gómez-Ayala et al., 2010)
**602**	S	ClC1=CC=C(C=C1)[C@@H]1CC2CC3=CC(Cl)=CC=C3N1O2	*L. chagasi*	IA	ND	100	(Gómez-Ayala et al., 2010)

Comp[a]	Ori[b]	SMILES[c]	*Leishmania* species[d]	Parasite form[e]	Leishmanicidal Potential[f]	EC_{50} (μM)[g]	Reference
**603**	S	ClC1=CC=C2N3OC(C[C@H]3C3=CC=C(Br)C=C3)CC2=C1	*L. chagasi*	IA	ND	95.42	(Gómez-Ayala et al., 2010)
**604**	S	FC1=CC=C2N3OC(C[C@H]3C3=CC=C(Cl)C=C3)CC2=C1	*L. chagasi*	IA	ND	100	(Gómez-Ayala et al., 2010)
**605**	S	ClC1=CC=CC=C1[C@@H]1CC2CC3=CC=CC=C3N1O2	*L. chagasi*	IA	ND	100	(Gómez-Ayala et al., 2010)
**606**	S	FC1=CC=CC=C1[C@@H]1CC2CC3=CC=CC=C3N1O2	*L. chagasi*	IA	ND	100	(Gómez-Ayala et al., 2010)
**607**	S	CC1=CC=C2N3OC(C[C@H]3C3=CC=CC=C3Cl)CC2=C1	*L. chagasi*	IA	ND	100	(Gómez-Ayala et al., 2010)
**608**	S	ClC1=CC=C2N3OC(C[C@H]3C3=CC=CC=C3Cl)CC2=C1	*L. chagasi*	IA	ND	100	(Gómez-Ayala et al., 2010)
**609**	S	FC1=CC=CC=C1[C@@H]1CC2CC3=CC(Cl)=CC=C3N1O2	*L. chagasi*	IA	ND	100	(Gómez-Ayala et al., 2010)
**610**	S	ClC1=CC=CC(Cl)=C1[C@@H]1CC2CC3=CC=CC=C3N1O2	*L. chagasi*	IA	ND	100	(Gómez-Ayala et al., 2010)
**611**	S	FC1=C([C@@H]2CC3CC4=CC=CC=C4N2O3)C(Cl)=CC=C1	*L. chagasi*	IA	ND	100	(Gómez-Ayala et al., 2010)
**612**	S	ClC1=CC=C2N3OC(C[C@H]3C3=C(Cl)C=CC=C3Cl)CC2=C1	*L. chagasi*	IA	ND	98.23	(Gómez-Ayala et al., 2010)
**613**	S	FC1=C([C@@H]2CC3CC4=CC(Cl)=CC=C4N2O3)C(Cl)=CC=C1	*L. chagasi*	IA	ND	100	(Gómez-Ayala et al., 2010)
**614**	S	O[C@H]1C[C@H](NC2=CC=CC=C2C1)C1=CC=CC=C1	*L. chagasi*	IA	ND	100	(Gómez-Ayala et al., 2010)
**615**	S	O[C@H]1C[C@H](NC2=CC=C(OC(F)(F)F)C=C2C1)C1=CC=CC=C1	*L. chagasi*	IA	ND	100	(Gómez-Ayala et al., 2010)
**616**	S	O[C@H]1C[C@H](NC2=CC=C(Br)C=C2C1)C1=CC=CC=C1	*L. chagasi*	IA	Low	97.65	(Gómez-Ayala et al., 2010)
**617**	S	O[C@H]1C[C@H](NC2=CC=C(Cl)C=C2C1)C1=CC=CC=C1	*L. chagasi*	IA	ND	100	(Gómez-Ayala et al., 2010)
**618**	S	O[C@H]1C[C@H](NC2=CC=C(F)C=C2C1)C1=CC=CC=C1	*L. chagasi*	IA	ND	100	(Gómez-Ayala et al., 2010)
**619**	S	O[C@H]1C[C@H](NC2=CC=CC=C2C1)C1=CC=C(Cl)C=C1	*L. chagasi*	IA	ND	100	(Gómez-Ayala et al., 2010)
**620**	S	O[C@H]1C[C@H](NC2=CC=CC=C2C1)C1=CC=C(F)C=C1	*L. chagasi*	IA	ND	100	(Gómez-Ayala et al., 2010)
**621**	S	COC1=CC(=CC=C1)[C@@H]1C[C@H](O)CC2=CC=CC=C2N1	*L. chagasi*	IA	ND	100	(Gómez-Ayala et al., 2010)

Comp[a]	Ori[b]	SMILES[c]	*Leishmania* species[d]	Parasite form[e]	Leishmanicidal Potential[f]	EC_{50} (μM)[g]	Reference
**622**	S	CC1=CC(=CC=C1)[C@@H]1C[C@H](O)CC2=CC=CC=C2N1	*L. chagasi*	IA	ND	100	(Gómez-Ayala et al., 2010)
**623**	S	O[C@H]1C[C@H](NC2=CC=CC=C2C1)C1=CC=CC(Cl)=C1	*L. chagasi*	IA	ND	100	(Gómez-Ayala et al., 2010)
**624**	S	CC1=CC=C(C=C1)[C@@H]1C[C@H](O)CC2=CC(C)=CC=C2N1	*L. chagasi*	IA	ND	100	(Gómez-Ayala et al., 2010)
**625**	S	O[C@H]1C[C@H](NC2=CC=C(Cl)C=C2C1)C1=CC=C(Cl)C=C1	*L. chagasi*	IA	ND	100	(Gómez-Ayala et al., 2010)
**626**	S	O[C@H]1C[C@H](NC2=CC=C(Cl)C=C2C1)C1=CC=C(Br)C=C1	*L. chagasi*	IA	ND	94.87	(Gómez-Ayala et al., 2010)
**627**	S	O[C@H]1C[C@H](NC2=CC=C(F)C=C2C1)C1=CC=C(Cl)C=C1	*L. chagasi*	IA	ND	100	(Gómez-Ayala et al., 2010)
**628**	S	O[C@H]1C[C@H](NC2=CC=CC=C2C1)C1=CC=CC=C1Cl	*L. chagasi*	IA	ND	100	(Gómez-Ayala et al., 2010)
**629**	S	O[C@H]1C[C@H](NC2=CC=CC=C2C1)C1=CC=CC=C1F	*L. chagasi*	IA	ND	100	(Gómez-Ayala et al., 2010)
**630**	S	CC1=CC=C2N[C@@H](C[C@H](O)CC2=C1)C1=CC=CC=C1Cl	*L. chagasi*	IA	ND	100	(Gómez-Ayala et al., 2010)
**631**	S	O[C@H]1C[C@H](NC2=CC=C(Cl)C=C2C1)C1=CC=CC=C1Cl	*L. chagasi*	IA	ND	100	(Gómez-Ayala et al., 2010)
**632**	S	O[C@H]1C[C@H](NC2=CC=C(Cl)C=C2C1)C1=CC=CC=C1F	*L. chagasi*	IA	ND	100	(Gómez-Ayala et al., 2010)
**633**	S	O[C@H]1C[C@H](NC2=CC=CC=C2C1)C1=C(Cl)C=CC=C1Cl	*L. chagasi*	IA	ND	100	(Gómez-Ayala et al., 2010)
**634**	S	O[C@H]1C[C@H](NC2=CC=CC=C2C1)C1=C(Cl)C=CC=C1F	*L. chagasi*	IA	ND	100	(Gómez-Ayala et al., 2010)
**635**	S	O[C@H]1C[C@H](NC2=CC=C(Cl)C=C2C1)C1=C(Cl)C=CC=C1Cl	*L. chagasi*	IA	ND	32.55	(Gómez-Ayala et al., 2010)
**636**	S	O[C@H]1C[C@H](NC2=CC=C(Cl)C=C2C1)C1=C(F)C=CC=C1Cl	*L. chagasi*	IA	Low	73.22	(Gómez-Ayala et al., 2010)
**637**	S	OC1=CC=C(\C=C\C(=O)OCCCOC2=CC(Cl)=CC=C2OC2=C(Cl)C=C(Cl)C=C2)C=C1O	*L. panamensis*	IA	Intermediate	24.34	(Otero et al., 2017)
**638**	S	OC1=CC=C(\C=C\C(=O)OCCCCOC2=CC(Cl)=CC=C2OC2=C(Cl)C=C(Cl)C=C2)C=C1O	*L. panamensis*	IA	High	3.82	(Otero et al., 2017)
**639**	S	OC1=CC=C(\C=C\C(=O)OCCCCCOC2=CC(Cl)=CC=C2OC2=C(Cl)C=C(Cl)C=C2)C=C1O	*L. panamensis*	IA	Intermediate	15.3	(Otero et al., 2017)
**640**	S	OC1=CC=C(\C=C\C(=O)OCCCCCCCOC2=CC(Cl)=CC=C2OC2=C(Cl)C=C(Cl)C=C2)C=C1O	*L. panamensis*	IA	Intermediate	11.65	(Otero et al., 2017)

Comp[a]	Ori[b]	SMILES[c]	*Leishmania* species[d]	Parasite form[e]	Leishmanicidal Potential[f]	EC_{50} (μM)[g]	Reference
**641**	S	OC1=CC=C(\C=C\C(=O)OCCCCCCCCOC2=CC(Cl)=CC=C2OC2=C(Cl)C=C(Cl)C=C2)C=C1O	*L. panamensis*	IA	Intermediate	12.92	(Otero et al., 2017)
**642**	S	OC1=CC=C(\C=C\C(=O)OCCCCCCCCCOC2=CC(Cl)=CC=C2OC2=C(Cl)C=C(Cl)C=C2)C=C1O	*L. panamensis*	IA	Low	30.22	(Otero et al., 2017)
**643**	S	OC1=CC=C(\C=C\C(=O)OCCCCCCCCCCCCOC2=CC(Cl)=CC=C2OC2=C(Cl)C=C(Cl)C=C2)C=C1O	*L. panamensis*	IA	Low	42.55	(Otero et al., 2017)
**644**	S	COC1=CC=C(\C=C\C(=O)OCCCOC2=CC(Cl)=CC=C2OC2=C(Cl)C=C(Cl)C=C2)C=C1OC	*L. panamensis*	IA	Low	38.06	(Otero et al., 2017)
**645**	S	COC1=CC=C(\C=C\C(=O)OCCCCOC2=CC(Cl)=CC=C2OC2=C(Cl)C=C(Cl)C=C2)C=C1OC	*L. panamensis*	IA	Low	53.19	(Otero et al., 2017)
**646**	S	COC1=CC=C(\C=C\C(=O)OCCCCCCCOC2=CC(Cl)=CC=C2OC2=C(Cl)C=C(Cl)C=C2)C=C1OC	*L. panamensis*	IA	Low	99.05	(Otero et al., 2017)
**647**	S	COC1=CC=C(\C=C\C(=O)OCCCCCCCCCOC2=CC(Cl)=CC=C2OC2=C(Cl)C=C(Cl)C=C2)C=C1OC	*L. panamensis*	IA	Low	48.46	(Otero et al., 2017)
**648**	S	COC1=CC=C(CCC(=O)OCCCOC2=CC(Cl)=CC=C2OC2=C(Cl)C=C(Cl)C=C2)C=C1OC	*L. panamensis*	IA	Low	40.25	(Otero et al., 2017)
**649**	S	[O-][N+](=O)C1=CC(=CC=C1)C1CC(N2CCCC2=O)C2=CC(Br)=CC=C2N1	*L. chagasi*	IA	ND	100	(Kouznetsov et al., 2007)
**650**	S	CC1=CC=C2NC(CC(N3CCCC3=O)C2=C1)C1=CC=CC(=C1)[N+]([O-])=O	*L. chagasi*	IA	ND	100	(Kouznetsov et al., 2007)
**651**	S	CCC1=CC=C2NC(CC(N3CCCC3=O)C2=C1)C1=CC=CC(=C1)[N+]([O-])=O	*L. chagasi*	IA	ND	100	(Kouznetsov et al., 2007)
**652**	S	ClC1=CC=C2N=C(C=CC2=C1)C1=CC=CO1	*L. chagasi*	IA	ND	100	(Kouznetsov et al., 2007)
**653**	S	BrC1=CC=C2N=C(C=CC2=C1)C1=CC=CO1	*L. chagasi*	IA	ND	100	(Kouznetsov et al., 2007)
**654**	S	CC1=CC=C2N=C(C=CC2=C1)C1=CC=CO1	*L. chagasi*	IA	ND	100	(Kouznetsov et al., 2007)
**655**	S	COC1=CC=C2N=C(C=CC2=C1)C1=CC=CO1	*L. chagasi*	IA	ND	100	(Kouznetsov et al., 2007)
**656**	S	COC1=CC(OC)=C2N=C(C=CC2=C1)C1=CC=CO1	*L. chagasi*	IA	ND	100	(Kouznetsov et al., 2007)
**657**	S	ClC1=CC=C2N=C(C=CC2=C1)C1=CC=CS1	*L. chagasi*	IA	ND	100	(Kouznetsov et al., 2007)
**658**	S	CC1=CC=C2N=C(C=CC2=C1)C1=CC=CS1	*L. chagasi*	IA	ND	100	(Kouznetsov et al., 2007)
**659**	S	COC1=CC=C2N=C(C=CC2=C1)C1=CC=CS1	*L. chagasi*	IA	ND	100	(Kouznetsov et al., 2007)
**660**	S	COC1=CC(OC)=C2N=C(C=CC2=C1)C1=CC=CS1	*L. chagasi*	IA	ND	100	(Kouznetsov et al., 2007)
**661**	S	CC(C)C1=C2N=C(C=C(C)C2=CC=C1)C1=CC=CN=C1	*L. chagasi*	IA	Intermediate	24.05	(Kouznetsov et al., 2007)
**662**	S	CC(C)C1=C2N=C(C=C(C)C2=CC=C1)C1=CC=NC=C1	*L. chagasi*	IA	ND	100	(Kouznetsov et al., 2007)
**663**	S	C1=CC=C(C=C1)C1=NC2=CC=CC=C2C=C1	*L. chagasi*	IA	ND	100	(Kouznetsov et al., 2007)
**664**	S	[O-][N+](=O)C1=CC(=CC=C1)C1=NC2=CC=C(Cl)C=C2C=C1	*L. chagasi*	IA	ND	100	(Kouznetsov et al., 2007)
**665**	S	NC1=CC(=CC=C1)C1=NC2=CC=C(Cl)C=C2C=C1	*L. chagasi*	IA	ND	100	(Kouznetsov et al., 2007)
**666**	S	O=C1CCSC2=CC=C(F)C=C21	*L. panamensis*	IA	ND	109.9	(Vargas et al., 2018)
**667**	S	O=C1CC(C)SC2=CC=CC=C21	*L. panamensis*	IA	Low	444.6	(Vargas et al., 2018)
**668**	S	O=C1CC(C)SC2=CC=C(F)C=C21	*L. panamensis*	IA	Low	422	(Vargas et al., 2018)

Comp[a]	Ori[b]	SMILES[c]	*Leishmania* species[d]	Parasite form[e]	Leishmanicidal Potential[f]	EC_{50} (μM)[g]	Reference
**669**	S	O=C1CC(C2=CC=CC=C2)SC3=CC=CC=C31	*L. panamensis*	IA	Low	44.1	(Vargas et al., 2018)
**670**	S	O=C(C1=CC=NC=C1)N/N=C2CCSC3=CC=CC=C3\2	*L. panamensis*	IA	Low	56.8	(Vargas et al., 2018)
**671**	S	NC(N/N=C1CCSC2=CC=CC=C2\1)=O	*L. panamensis*	IA	Low	91.5	(Vargas et al., 2018)
**672**	S	NC(N/N=C1CCSC2=CC=CC=C2\1)=S	*L. panamensis*	IA	Low	55.7	(Vargas et al., 2018)
**673**	S	O=C(C1=CC=CC=C1)N/N=C2CCSC3=CC=C(F)C=C3\2	*L. panamensis*	IA	Low	37.3	(Vargas et al., 2018)
**674**	S	O=C(C1=CC=NC=C1)N/N=C2CCSC3=CC=C(F)C=C3\2	*L. panamensis*	IA	Low	39.9	(Vargas et al., 2018)
**675**	S	O=C(C1=CC=C(N)C=C1)N/N=C2CCSC3=CC=C(F)C=C3\2	*L. panamensis*	IA	Low	95.5	(Vargas et al., 2018)
**676**	S	O=C(C1=CC=CC=C1)N/N=C2CC(C)SC3=CC=CC=C3\2	*L. panamensis*	IA	Low	38.1	(Vargas et al., 2018)
**677**	S	O=C(C1=CC=NC=C1)N/N=C2CC(C)SC3=CC=CC=C3\2	*L. panamensis*	IA	Low	56.6	(Vargas et al., 2018)
**678**	S	O=C(C1=CC=C(N)C=C1)N/N=C2CC(C)SC3=CC=CC=C3\2	*L. panamensis*	IA	Low	91.8	(Vargas et al., 2018)
**679**	S	O=C(C1=CC=CC=C1)N/N=C2CC(C)SC3=CC=C(F)C=C3\2	*L. panamensis*	IA	Low	43.9	(Vargas et al., 2018)
**680**	S	O=C(C1=CC=NC=C1)N/N=C2CC(C)SC3=CC=C(F)C=C3\2	*L. panamensis*	IA	Low	98.9	(Vargas et al., 2018)
**681**	S	O=C(C1=CC=C(N)C=C1)N/N=C2CC(C)SC3=CC=C(F)C=C3\2	*L. panamensis*	IA	Low	160.7	(Vargas et al., 2018)
**682**	S	COC1=CC(C=O)=CC=C1OC2=CC=NC3=CC(Cl)=CC=C32	*L. donovani*	IA	High	0.66	(Valdivieso et al., 2018)
**682**	S	COC1=CC(C=O)=CC=C1OC2=CC=NC3=CC(Cl)=CC=C32	*L. donovani*	P	Intermediate	13.03	(Valdivieso et al., 2018)
**683**	S	COC1=CC(/C=N/NC(N)=S)=CC=C1OC2=CC=NC3=CC(Cl)=CC=C32	*L. donovani*	IA	High	1.02	(Valdivieso et al., 2018)
**683**	S	COC1=CC(/C=N/NC(N)=S)=CC=C1OC2=CC=NC3=CC(Cl)=CC=C32	*L. donovani*	P	High	7.9	(Valdivieso et al., 2018)
**684**	S	C[C@H]1[C@H](C2=CC=CC=C2)NC3=CC=CC=C3[C@@H]1C4=CC=C(OC)C=C4	*L. chagasi*	IA	ND	90	(Bohórquez et al., 2012)
**684**	S	C[C@H]1[C@H](C2=CC=CC=C2)NC3=CC=CC=C3[C@@H]1C4=CC=C(OC)C=C4	*L. chagasi*	P	Low	64.29	(Bohórquez et al., 2012)
**685**	S	C[C@H]1[C@H](C2=CC=CC=C2)NC3=CC=C(C)C=C3[C@@H]1C4=CC=C(OC)C=C4	*L. chagasi*	IA	N/A		(Bohórquez et al., 2012)
**685**	S	C[C@H]1[C@H](C2=CC=CC=C2)NC3=CC=C(C)C=C3[C@@H]1C4=CC=C(OC)C=C4	*L. chagasi*	P	N/A		(Bohórquez et al., 2012)
**686**	S	C[C@H]1[C@H](C2=CC=CC=C2)NC3=CC=C(CC)C=C3[C@@H]1C4=CC=C(OC)C=C4	*L. chagasi*	IA	ND	90	(Bohórquez et al., 2012)
**686**	S	C[C@H]1[C@H](C2=CC=CC=C2)NC3=CC=C(CC)C=C3[C@@H]1C4=CC=C(OC)C=C4	*L. chagasi*	P	Intermediate	17.62	(Bohórquez et al., 2012)
**687**	S	C[C@H]1[C@H](C2=CC=CC=C2)NC3=CC=C(OC)C=C3[C@@H]1C4=CC=C(OC)C=C4	*L. chagasi*	IA	ND	90	(Bohórquez et al., 2012)
**687**	S	C[C@H]1[C@H](C2=CC=CC=C2)NC3=CC=C(OC)C=C3[C@@H]1C4=CC=C(OC)C=C4	*L. chagasi*	P	High	4.34	(Bohórquez et al., 2012)
**688**	S	C[C@H]1[C@H](C2=CC=CC=C2)NC3=CC=C(Cl)C=C3[C@@H]1C4=CC=C(OC)C=C4	*L. chagasi*	IA	ND	90	(Bohórquez et al., 2012)
**688**	S	C[C@H]1[C@H](C2=CC=CC=C2)NC3=CC=C(Cl)C=C3[C@@H]1C4=CC=C(OC)C=C4	*L. chagasi*	P	Intermediate	12.45	(Bohórquez et al., 2012)
**689**	S	C[C@H]1[C@H](C2=CC=CC=C2)NC3=CC=C([N+]([O-])=O)C=C3[C@@H]1C4=CC=C(OC)C=C4	*L. chagasi*	IA	ND	90	(Bohórquez et al., 2012)

Comp[a]	Ori[b]	SMILES[c]	*Leishmania* species[d]	Parasite form[e]	Leishmanicidal Potential[f]	EC_{50} (μM)[g]	Reference
**689**	S	C[C@H]1[C@H](C2=CC=CC=C2)NC3=CC=C([N+]([O-])=O)C=C3[C@@H]1C4=CC=C(OC)C=C4	*L. chagasi*	P	High	4.25	(Bohórquez et al., 2012)
**690**	S	C[C@H]1[C@H](C2=CC=CC=C2)NC3=CC([N+]([O-])=O)=CC=C3[C@@H]1C4=CC=C(OC)C=C4	*L. chagasi*	IA	ND	90	(Bohórquez et al., 2012)
**690**	S	C[C@H]1[C@H](C2=CC=CC=C2)NC3=CC([N+]([O-])=O)=CC=C3[C@@H]1C4=CC=C(OC)C=C4	*L. chagasi*	P	Low	29.51	(Bohórquez et al., 2012)
**691**	S	C[C@H]1[C@H](C2=CC=CC=C2)NC3=C([N+]([O-])=O)C=CC=C3[C@@H]1C4=CC=C(OC)C=C4	*L. chagasi*	IA	ND	90	(Bohórquez et al., 2012)
**691**	S	C[C@H]1[C@H](C2=CC=CC=C2)NC3=C([N+]([O-])=O)C=CC=C3[C@@H]1C4=CC=C(OC)C=C4	*L. chagasi*	P	High	0.27	(Bohórquez et al., 2012)
**692**	S	C[C@H]1[C@H](C2=CC=CC=C2)NC3=CC=CC([N+]([O-])=O)=C3[C@@H]1C4=CC=C(OC)C=C4	*L. chagasi*	IA	ND	90	(Bohórquez et al., 2012)
**692**	S	C[C@H]1[C@H](C2=CC=CC=C2)NC3=CC=CC([N+]([O-])=O)=C3[C@@H]1C4=CC=C(OC)C=C4	*L. chagasi*	P	Intermediate	11.06	(Bohórquez et al., 2012)
**693**	S	C[C@H]1[C@H](C2=CC=CC=C2)NC3=C(C#N)C=CC=C3[C@@H]1C4=CC=C(OC)C=C4	*L. chagasi*	IA	N/A		(Bohórquez et al., 2012)
**693**	S	C[C@H]1[C@H](C2=CC=CC=C2)NC3=C(C#N)C=CC=C3[C@@H]1C4=CC=C(OC)C=C4	*L. chagasi*	P	N/A		(Bohórquez et al., 2012)
**694**	S	CC1=CC=C(N[C@H](C2=CC=CC=N2)[C@H]3[C@@H]4C5=C(C=CC=C5)C3)C4=C1	*L. mexicana*	P	High	1.01	(Hernández-Chinea et al., 2015)
**695**	S	ClC1=CC=C(N[C@H](C2=CC=CC=N2)[C@H]3[C@@H]4C5=C(C=CC=C5)C3)C4=C1	*L. mexicana*	P	High	4.49	(Hernández-Chinea et al., 2015)
**696**	S	CCC1=CC=C(N[C@H](C2=CC=CC=N2)[C@H]3[C@@H]4C5=C(C=CC=C5)C3)C4=C1	*L. mexicana*	P	High	1.02	(Hernández-Chinea et al., 2015)
**697**	S	BrC1=C(N[C@H](C2=CC=CC=N2)[C@H]3[C@@H]4C5=C(C=CC=C5)C3)C4=CC=C1	*L. mexicana*	P	High	2.8	(Hernández-Chinea et al., 2015)
**698**	S	CC1=CC(C)=C(N[C@H](C2=CC=CC=N2)[C@H]3[C@@H]4C5=C(C=CC=C5)C3)C4=C1	*L. mexicana*	P	High	2.09	(Hernández-Chinea et al., 2015)
**699**	S	CC1=C(N[C@H](C2=CC=CC=N2)[C@H]3[C@@H]4C5=C(C=CC=C5)C3)C4=C(C)C=C1	*L. mexicana*	P	High	3.23	(Hernández-Chinea et al., 2015)
**700**	S	O=C(C)C1=CC=C(N[C@H](C2=CC=CC=N2)[C@H]3[C@@H]4C5=C(C=CC=C5)C3)C4=C1	*L. mexicana*	P	High	4.83	(Hernández-Chinea et al., 2015)
**701**	S	CC1=C([N+]([O-])=O)C(OC)=C(C(C)C)C=C1NN(C2=CC([N+]([O-])=O)=C(OC)C(C(C)C)=C2)O	*L. panamensis*	IA	Low	468.545	(Robledo et al., 2005)
**701**	S	CC1=C([N+]([O-])=O)C(OC)=C(C(C)C)C=C1NN(C2=CC([N+]([O-])=O)=C(OC)C(C(C)C)=C2)O	*L. panamensis*	P	Intermediate	22.312	(Robledo et al., 2005)
**702**		[H][N+]1=C2C(C)=C(CC)/C1=C/C3=NC(CC4=C(CC)C(C)=C(N4)/C=C5C(C=CC=C6)=C6/C(C/5(OC)OC)=C/2)C(CC)=C3CC	*L. amazonensis*	AA	High	2.4	(Taylor et al., 2011)

Comp[a]	Ori[b]	SMILES[c]	*Leishmania* species[d]	Parasite form[e]	Leishmanicidal Potential[f]	EC_{50} (μM)[g]	Reference
**702**		[H][N+]1=C2C(C)=C(CC)/C1=C/C3=NC(CC4=C(CC)C(C)=C(N4)/C=C5C(C=CC=C6)=C6/C(C/5(OC)OC)=C/2)C(CC)=C3CC	*L. panamensis*	AA	High	0.5	(Taylor et al., 2011)
**702**		[H][N+]1=C2C(C)=C(CC)/C1=C/C3=NC(CC4=C(CC)C(C)=C(N4)/C=C5C(C=CC=C6)=C6/C(C/5(OC)OC)=C/2)C(CC)=C3CC	*L. infantum*	AA	High	0.4	(Taylor et al., 2011)
**703**		[H][N+]1=C2C(C)=C(CC)/C1=C/C3=NC(CC4=C(CC)C(C)=C(N4)/C=C5C(C=CC=C6)=C6/C(C/5(OCC)OCC)=C/2)C(CC)=C3CC	*L. amazonensis*	AA	High	5.3	(Taylor et al., 2011)
**703**		[H][N+]1=C2C(C)=C(CC)/C1=C/C3=NC(CC4=C(CC)C(C)=C(N4)/C=C5C(C=CC=C6)=C6/C(C/5(OCC)OCC)=C/2)C(CC)=C3CC	*L. panamensis*	AA	High	2.2	(Taylor et al., 2011)
**703**		[H][N+]1=C2C(C)=C(CC)/C1=C/C3=NC(CC4=C(CC)C(C)=C(N4)/C=C5C(C=CC=C6)=C6/C(C/5(OCC)OCC)=C/2)C(CC)=C3CC	*L. infantum*	AA	High	0.72	(Taylor et al., 2011)
**704**	S	C1(CNCCNCC2=CC=CC=C2)=CC=CC=C1	*L. infantum*	IA	Low	25.42	(Leal et al., 2013a)
**704**	S	C1(CNCCNCC2=CC=CC=C2)=CC=CC=C1	*L. panamensis*	IA	Low	58.2	(Leal et al., 2013a)
**704**	S	C1(CNCCNCC2=CC=CC=C2)=CC=CC=C1	*L. amazonensis*	IA	Low	49.27	(Leal et al., 2013a)
**704**	S	C1(CNCCNCC2=CC=CC=C2)=CC=CC=C1	*L. infantum*	P	Intermediate	12.19	(Leal et al., 2013a)
**705**	S	C1(CNCCCNCC2=CC=CC=C2)=CC=CC=C1	*L. infantum*	IA	N/A	0	(Leal et al., 2013a)
**705**	S	C1(CNCCCNCC2=CC=CC=C2)=CC=CC=C1	*L. panamensis*	IA	N/A	0	(Leal et al., 2013a)
**705**	S	C1(CNCCCNCC2=CC=CC=C2)=CC=CC=C1	*L. amazonensis*	IA	N/A	0	(Leal et al., 2013a)
**705**	S	C1(CNCCCNCC2=CC=CC=C2)=CC=CC=C1	*L. infantum*	P	High	1.96	(Leal et al., 2013a)
**706**	S	C1(CNCCCCNCC2=CC=CC=C2)=CC=CC=C1	*L. infantum*	IA	N/A		(Leal et al., 2013a)
**706**	S	C1(CNCCCCNCC2=CC=CC=C2)=CC=CC=C1	*L. panamensis*	IA	Intermediate	11.19	(Leal et al., 2013a)
**706**	S	C1(CNCCCCNCC2=CC=CC=C2)=CC=CC=C1	*L. amazonensis*	IA	N/A		(Leal et al., 2013a)
**706**	S	C1(CNCCCCNCC2=CC=CC=C2)=CC=CC=C1	*L. infantum*	P	Intermediate	11.23	(Leal et al., 2013a)
**707**	S	OC1=CC(CNCCNCC2=CC=C(OC)C(C)=C2)=CC=C1OC	*L. infantum*	IA	N/A		(Leal et al., 2013a)
**707**	S	OC1=CC(CNCCNCC2=CC=C(OC)C(C)=C2)=CC=C1OC	*L. panamensis*	IA	N/A		(Leal et al., 2013a)
**707**	S	OC1=CC(CNCCNCC2=CC=C(OC)C(C)=C2)=CC=C1OC	*L. amazonensis*	IA	N/A		(Leal et al., 2013a)
**707**	S	OC1=CC(CNCCNCC2=CC=C(OC)C(C)=C2)=CC=C1OC	*L. infantum*	P	Low	40.24	(Leal et al., 2013a)
**708**	S	OC(C=C1)=C(OC)C=C1CNCCCNCC2=CC=C(OC)C(O)=C2	*L. infantum*	IA	N/A	0	(Leal et al., 2013a)
**708**	S	OC(C=C1)=C(OC)C=C1CNCCCNCC2=CC=C(OC)C(O)=C2	*L. panamensis*	IA	N/A	0	(Leal et al., 2013a)
**708**	S	OC(C=C1)=C(OC)C=C1CNCCCNCC2=CC=C(OC)C(O)=C2	*L. amazonensis*	IA	N/A	0	(Leal et al., 2013a)
**708**	S	OC(C=C1)=C(OC)C=C1CNCCCNCC2=CC=C(OC)C(O)=C2	*L. infantum*	P	ND	250	(Leal et al., 2013a)
**709**	S	C1(CNCCNCC2=CC=NC=C2)=CC=NC=C1	*L. infantum*	IA	N/A	0	(Leal et al., 2013a)
**709**	S	C1(CNCCNCC2=CC=NC=C2)=CC=NC=C1	*L. panamensis*	IA	N/A	0	(Leal et al., 2013a)
**709**	S	C1(CNCCNCC2=CC=NC=C2)=CC=NC=C1	*L. amazonensis*	IA	N/A	0	(Leal et al., 2013a)
**709**	S	C1(CNCCNCC2=CC=NC=C2)=CC=NC=C1	*L. infantum*	P	Low	180.54	(Leal et al., 2013a)
**710**	S	C1(CNCCCCNCC2=NC=CC=C2)=NC=CC=C1	*L. infantum*	IA	N/A	0	(Leal et al., 2013a)
**710**	S	C1(CNCCCCNCC2=NC=CC=C2)=NC=CC=C1	*L. panamensis*	IA	N/A	0	(Leal et al., 2013a)
**710**	S	C1(CNCCCCNCC2=NC=CC=C2)=NC=CC=C1	*L. amazonensis*	IA	N/A	0	(Leal et al., 2013a)
**710**	S	C1(CNCCCCNCC2=NC=CC=C2)=NC=CC=C1	*L. infantum*	P	Low	28.83	(Leal et al., 2013a)

Comp[a]	Ori[b]	SMILES[c]	*Leishmania* species[d]	Parasite form[e]	Leishmanicidal Potential[f]	EC_{50} (μM)[g]	Reference
**711**	S	C1(CNCCCCNCC2=CN=CC=C2)=CN=CC=C1	*L. infantum*	IA	N/A	0	(Leal et al., 2013a)
**711**	S	C1(CNCCCCNCC2=CN=CC=C2)=CN=CC=C1	*L. panamensis*	IA	N/A	0	(Leal et al., 2013a)
**711**	S	C1(CNCCCCNCC2=CN=CC=C2)=CN=CC=C1	*L. amazonensis*	IA	N/A	0	(Leal et al., 2013a)
**711**	S	C1(CNCCCCNCC2=CN=CC=C2)=CN=CC=C1	*L. infantum*	P	Low	44.19	(Leal et al., 2013a)
**712**	S	O=C(CSC1C2=CC=CC=C2)N1CCN3C(CSC3C4=CC=CC=C4)=O	*L. infantum*	IA	N/A	0	(Leal et al., 2013a)
**712**	S	O=C(CSC1C2=CC=CC=C2)N1CCN3C(CSC3C4=CC=CC=C4)=O	*L. panamensis*	IA	N/A	0	(Leal et al., 2013a)
**712**	S	O=C(CSC1C2=CC=CC=C2)N1CCN3C(CSC3C4=CC=CC=C4)=O	*L. amazonensis*	IA	N/A	0	(Leal et al., 2013a)
**712**	S	O=C(CSC1C2=CC=CC=C2)N1CCN3C(CSC3C4=CC=CC=C4)=O	*L. infantum*	P	Low	135.8	(Leal et al., 2013a)
**713**	S	O=C(CSC1C2=CC=CC=C2)N1CCCN3C(CSC3C4=CC=CC=C4)=O	*L. infantum*	IA	N/A	0	(Leal et al., 2013a)
**713**	S	O=C(CSC1C2=CC=CC=C2)N1CCCN3C(CSC3C4=CC=CC=C4)=O	*L. panamensis*	IA	N/A	0	(Leal et al., 2013a)
**713**	S	O=C(CSC1C2=CC=CC=C2)N1CCCN3C(CSC3C4=CC=CC=C4)=O	*L. amazonensis*	IA	N/A	0	(Leal et al., 2013a)
**713**	S	O=C(CSC1C2=CC=CC=C2)N1CCCN3C(CSC3C4=CC=CC=C4)=O	*L. infantum*	P	ND	250	(Leal et al., 2013a)
**714**	S	O=C(CSC1C2=NC=CC=C2)N1CCN3C(CSC3C4=NC=CC=C4)=O	*L. infantum*	IA	N/A	0	(Leal et al., 2013a)
**714**	S	O=C(CSC1C2=NC=CC=C2)N1CCN3C(CSC3C4=NC=CC=C4)=O	*L. panamensis*	IA	N/A	0	(Leal et al., 2013a)
**714**	S	O=C(CSC1C2=NC=CC=C2)N1CCN3C(CSC3C4=NC=CC=C4)=O	*L. amazonensis*	IA	N/A	0	(Leal et al., 2013a)
**714**	S	O=C(CSC1C2=NC=CC=C2)N1CCN3C(CSC3C4=NC=CC=C4)=O	*L. infantum*	P	Low	176.42	(Leal et al., 2013a)
**715**	S	O=C(CSC1C2=CN=CC=C2)N1CCN3C(CSC3C4=CN=CC=C4)=O	*L. infantum*	IA	N/A	0	(Leal et al., 2013a)
**715**	S	O=C(CSC1C2=CN=CC=C2)N1CCN3C(CSC3C4=CN=CC=C4)=O	*L. panamensis*	IA	N/A	0	(Leal et al., 2013a)
**715**	S	O=C(CSC1C2=CN=CC=C2)N1CCN3C(CSC3C4=CN=CC=C4)=O	*L. amazonensis*	IA	N/A	0	(Leal et al., 2013a)
**715**	S	O=C(CSC1C2=CN=CC=C2)N1CCN3C(CSC3C4=CN=CC=C4)=O	*L. infantum*	P	ND	250	(Leal et al., 2013a)
**716**	S	O=C(CSC1C2=CC=NC=C2)N1CCN3C(CSC3C4=CC=NC=C4)=O	*L. infantum*	IA	N/A	0	(Leal et al., 2013a)
**716**	S	O=C(CSC1C2=CC=NC=C2)N1CCN3C(CSC3C4=CC=NC=C4)=O	*L. panamensis*	IA	N/A	0	(Leal et al., 2013a)
**716**	S	O=C(CSC1C2=CC=NC=C2)N1CCN3C(CSC3C4=CC=NC=C4)=O	*L. amazonensis*	IA	N/A	0	(Leal et al., 2013a)
**716**	S	O=C(CSC1C2=CC=NC=C2)N1CCN3C(CSC3C4=CC=NC=C4)=O	*L. infantum*	P	ND	250	(Leal et al., 2013a)
**717**	S	O=C(CSC1C2=NC=CC=C2)N1CCCCN3C(CSC3C4=NC=CC=C4)=O	*L. infantum*	IA	N/A	0	(Leal et al., 2013a)
**717**	S	O=C(CSC1C2=NC=CC=C2)N1CCCCN3C(CSC3C4=NC=CC=C4)=O	*L. panamensis*	IA	N/A	0	(Leal et al., 2013a)
**717**	S	O=C(CSC1C2=NC=CC=C2)N1CCCCN3C(CSC3C4=NC=CC=C4)=O	*L. amazonensis*	IA	N/A	0	(Leal et al., 2013a)
**717**	S	O=C(CSC1C2=NC=CC=C2)N1CCCCN3C(CSC3C4=NC=CC=C4)=O	*L. infantum*	P	ND	250	(Leal et al., 2013a)
**718**	S	O=C(CSC1C2=CN=CC=C2)N1CCCCN3C(CSC3C4=CN=CC=C4)=O	*L. infantum*	IA	N/A	0	(Leal et al., 2013a)
**718**	S	O=C(CSC1C2=CN=CC=C2)N1CCCCN3C(CSC3C4=CN=CC=C4)=O	*L. panamensis*	IA	N/A	0	(Leal et al., 2013a)
**718**	S	O=C(CSC1C2=CN=CC=C2)N1CCCCN3C(CSC3C4=CN=CC=C4)=O	*L. amazonensis*	IA	N/A	0	(Leal et al., 2013a)
**718**	S	O=C(CSC1C2=CN=CC=C2)N1CCCCN3C(CSC3C4=CN=CC=C4)=O	*L. infantum*	P	ND	250	(Leal et al., 2013a)
**719**	S	O=C(CSC1C2=CC=NC=C2)N1CCCCN3C(CSC3C4=CC=NC=C4)=O	*L. infantum*	IA	N/A	0	(Leal et al., 2013a)
**719**	S	O=C(CSC1C2=CC=NC=C2)N1CCCCN3C(CSC3C4=CC=NC=C4)=O	*L. panamensis*	IA	N/A	0	(Leal et al., 2013a)
**719**	S	O=C(CSC1C2=CC=NC=C2)N1CCCCN3C(CSC3C4=CC=NC=C4)=O	*L. amazonensis*	IA	N/A	0	(Leal et al., 2013a)
**719**	S	O=C(CSC1C2=CC=NC=C2)N1CCCCN3C(CSC3C4=CC=NC=C4)=O	*L. infantum*	P	ND	250	(Leal et al., 2013a)
**720**	S	O=C(CCl)NCCNC(CCl)=O	*L. infantum*	IA	N/A	0	(Leal et al., 2013a)
**720**	S	O=C(CCl)NCCNC(CCl)=O	*L. panamensis*	IA	N/A	0	(Leal et al., 2013a)

Comp[a]	Ori[b]	SMILES[c]	*Leishmania* species[d]	Parasite form[e]	Leishmanicidal Potential[f]	EC_{50} (μM)[g]	Reference
**720**	S	O=C(CCl)NCCNC(CCl)=O	*L. amazonensis*	IA	N/A	0	(Leal et al., 2013a)
**720**	S	O=C(CCl)NCCNC(CCl)=O	*L. infantum*	P	Low	176.02	(Leal et al., 2013a)
**721**	S	O=C(CCl)NCCCNC(CCl)=O	*L. infantum*	IA	N/A	0	(Leal et al., 2013a)
**721**	S	O=C(CCl)NCCCNC(CCl)=O	*L. panamensis*	IA	N/A	0	(Leal et al., 2013a)
**721**	S	O=C(CCl)NCCCNC(CCl)=O	*L. amazonensis*	IA	N/A	0	(Leal et al., 2013a)
**721**	S	O=C(CCl)NCCCNC(CCl)=O	*L. infantum*	P	Low	76.43	(Leal et al., 2013a)
**722**	S	O=C(CCl)NCCCCNC(CCl)=O	*L. infantum*	IA	N/A	0	(Leal et al., 2013a)
**722**	S	O=C(CCl)NCCCCNC(CCl)=O	*L. panamensis*	IA	N/A	0	(Leal et al., 2013a)
**722**	S	O=C(CCl)NCCCCNC(CCl)=O	*L. amazonensis*	IA	N/A	0	(Leal et al., 2013a)
**722**	S	O=C(CCl)NCCCCNC(CCl)=O	*L. infantum*	P	Low	132.17	(Leal et al., 2013a)
**723**	S	O=C(CCl)N(CC1=CC=CC=C1)CCCCN(CC2=CC=CC=C2)C(CCl)=O	*L. infantum*	IA	N/A	0	(Leal et al., 2013a)
**723**	S	O=C(CCl)N(CC1=CC=CC=C1)CCCCN(CC2=CC=CC=C2)C(CCl)=O	*L. panamensis*	IA	N/A	0	(Leal et al., 2013a)
**723**	S	O=C(CCl)N(CC1=CC=CC=C1)CCCCN(CC2=CC=CC=C2)C(CCl)=O	*L. amazonensis*	IA	N/A	0	(Leal et al., 2013a)
**723**	S	O=C(CCl)N(CC1=CC=CC=C1)CCCCN(CC2=CC=CC=C2)C(CCl)=O	*L. infantum*	P	High	3.91	(Leal et al., 2013a)
**724**	S	CC(S1)=CN=C1N(CCN2)C2=O	*L. infantum*	P	Low	163.8	(Alvarez et al., 2002)
**725**	S	CC(S1)=CN=C1N(CCN2CC3=C(Br)C=CC=C3)C2=O	*L. infantum*	P	Intermediate	20.4	(Alvarez et al., 2002)
**726**	S	CC(S1)=CN=C1N(CCN2S(C3=CC=C(C)C=C3)(=O)=O)C2=O	*L. infantum*	P	Intermediate	17	(Alvarez et al., 2002)
**727**	S	CC(S1)=CN=C1N(CCN2S(C3=CC=C(C(F)(F)F)C=C3)(=O)=O)C2=O	*L. infantum*	P	Low	76.6	(Alvarez et al., 2002)
**728**	S	O=C1NCCN1C2=CC(C)=NO2	*L. infantum*	P	Low	179.4	(Alvarez et al., 2002)
**729**	S	O=C1N(CC2=C(Br)C=CC=C2)CCN1C3=CC(C)=NO3	*L. infantum*	P	Low	33.9	(Alvarez et al., 2002)
**730**	S	O=C1N(CC2=CC=C(Br)C=C2)CCN1C3=CC(C)=NO3	*L. infantum*	P	High	9.5	(Alvarez et al., 2002)
**731**	S	O=C1N(S(C2=CC=C(C)C=C2)(=O)=O)CCN1C3=CC(C)=NO3	*L. infantum*	P	Low	93.2	(Alvarez et al., 2002)
**732**	S	O=C(O1)C=CCC1C2=CC=C(O)C(OC)=C2	*L. panamensis*	IA	Low	172.215	(Castano et al., 2009)
**733**	S	O=C(O1)C=CCC1C2=CC=C(OCCCOC3=CC=C(C=C3OC)C4OC(C=CC4)=O)C(OC)=C2	*L. panamensis*	IA	Low	46.233	(Castano et al., 2009)
**734**	S	O=C1C=CCC(CCCCCCCCCCCCCCC)O1	*L. panamensis*	IA	High	2.595	(Castano et al., 2009)
**735**	S	CCCCCCCCCCCCCCCC1C=CC(O1)=O	*L. panamensis*	IA	High	9.516	(Castano et al., 2009)
**736**	S	O=C(O1)C=CCC1C2=CC=C(O)C(O)=C2	*L. panamensis*	IA	Intermediate	21.839	(Castano et al., 2009)
**737**	S	O=C(O1)C=CCC1C2=CC=C(OC)C(OC)=C2	*L. panamensis*	IA	High	6.835	(Castano et al., 2009)
**738**	S	O=C(O1)C=CCC1C2=CC=C(OC)C(O)=C2	*L. panamensis*	IA	Low	39.078	(Castano et al., 2009)
**739**	S	O=C(O1)C=CCC1CCC2=CC=CC=C2	*L. panamensis*	IA	Low	37.11	(Castano et al., 2009)
**740**	S	O=C(O1)C=CCC1/C=C/C2=CC=CC=C2	*L. panamensis*	IA	High	9.496	(Castano et al., 2009)
**741**	S	O=C(O1)C=CCC1CCCCCCCCC2CC=CC(O2)=O	*L. panamensis*	IA	High	4.572	(Castano et al., 2009)
**742**	S	O=C(O1)CC(OC)CC1CCCCCCCCCCCCCCC	*L. panamensis*	IA	Low	138.702	(Castano et al., 2009)
**743**	S	O=C(N1)C=CC[C@@H]1CCCCCCCCCCCCCCC	*L. panamensis*	IA	Intermediate	11.13	(Castano et al., 2009)
**744**	S	O=C1O[C@@H](C[C@@H](O)C[C@@H](O)CC[C@@H](O)CCCCCCCCCCCCCC)CC=C1	*L. panamensis*	IA	High	1.135	(Cardona G. et al., 2004)

Comp[a]	Ori[b]	SMILES[c]	*Leishmania* species[d]	Parasite form[e]	Leishmanicidal Potential[f]	EC_{50} (μM)[g]	Reference
**745**	S	O=C1O[C@@H](C[C@@H](OC(C)=O)C[C@@H](O)CC[C@@H](O)CCCCCCCCCCCCCC)C[C@@H](O)C1	*L. panamensis*	IA	Low	47.964	(Cardona G. et al., 2004)
**746**	S	O=C1O[C@@H](C[C@@H](OC(C)=O)C[C@@H](O)CC[C@@H](O)CCCCCCCCCCCCCC)CC=C1	*L. panamensis*	IA	High	5.805	(Cardona G. et al., 2004)
**747**	S	O=C1O[C@@H](C[C@@H](OC(C)=O)C[C@@H](OC(C)=O)CC[C@@H](OC(C)=O)CCCCCCCCCCCCCC)CC=C1	*L. panamensis*	IA	High	6.18	(Cardona G. et al., 2004)
**748**	S	O=C1O[C@@H](C[C@@H](O)CC(CCC(CCCCCCCCCCCCCC)=O)=O)CC=C1	*L. panamensis*	IA	Low	34.379	(Cardona G. et al., 2004)
**749**	S	O=C1O[C@@H](C[C@@H](OC(C)(C)O2)C[C@@H]2CC[C@@H](O)CCCCCCCCCCCCCC)CC=C1	*L. panamensis*	IA	High	5.412	(Cardona G. et al., 2004)
**750**	N	O[C@@]1([C@@]2([H])C[C@]([C@@]3(C)O)(C)C[C@@]2([H])OC3=O)CCC4[C@]5([H])C[C@@H]6[C@]([C@@]7(C)[C@@]5([H])CC[C@@]41C)(O6)CC=CC7=O	*L. panamensis*	IA	High	6.4	(Cardona et al., 2006a)
**751**	N	O[C@@]1([C@@]2([H])C[C@]([C@@]3(C)O)(C)C[C@@]2([H])OC3=O)CCC4[C@]5([H])CCC([C@@]6(C)[C@@]5([H])CC[C@@]41C)=CC=CC6=O	*L. panamensis*	IA	Low	121.2	(Cardona et al., 2006a)
**752**	N	O[C@@]1([C@@]2([H])C[C@]([C@@]3(C)O)(C)C[C@@]2([H])OC3=O)CCC4[C@]5([H])[C@H](OC(C)=O)[C@@H]6[C@]([C@@]7(C)[C@@]5([H])CC[C@@]41C)(O6)CC=CC7=O	*L. panamensis*	IA	High	4.9	(Cardona et al., 2006a)
**753**	N	O[C@@]1([C@@]2([H])C[C@]([C@@]3(C)O)(C)C[C@@]2([H])OC3=O)CCC4[C@]5([H])C[C@@H]6[C@]([C@@]7(C)[C@@]5([H])CC[C@@]41C)(O6)[C@@H](O)C=CC7=O	*L. panamensis*	IA	High	2.1	(Cardona et al., 2006a)
**754**	N	O[C@@]1([C@@]2([H])C[C@]([C@@]3(C)O)(C)C[C@@]2([H])OC3=O)CCC4[C@]5([H])C[C@H](O)[C@]([C@@]6(C)[C@@]5([H])CC[C@@]41C)(O)CC=CC6=O	*L. panamensis*	IA	Low	155.5	(Cardona et al., 2006a)
**755**	N	O[C@@]1([C@@]2([H])C[C@]([C@@]3(C)O)(C)C[C@@]2([H])OC3=O)CCC4[C@]5([H])C[C@@H]6[C@]([C@@]7(C)[C@@]5([H])CC[C@@]41C)(O6)CCCC7=O	*L. panamensis*	IA	Intermediate	16.7	(Cardona et al., 2006a)
**756**	N	O[C@]1([C@@]2([H])[C@](C[C@](O)(C)[C@@]3(C)C2)([H])OC3=O)CC[C@@]4(O)[C@]5([H])CC=C([C@@]6(C)[C@@]5([H])CC[C@@]41C)[C@@H](O)C=CC6=O	*L. panamensis*	IA	Low	63.3	(Cardona et al., 2006a)
**757**	N	O[C@]1([C@@]2([H])[C@](C[C@](O)(C)[C@@]3(C)C2)([H])OC3=O)CC[C@@]4(O)[C@]5([H])CC=C([C@@]6(C)[C@@]5([H])CC[C@@]41C)[C@@H](O)[C@@H](OC)CC6=O	*L. panamensis*	IA	ND	50	(Cardona et al., 2006a)
**758**	N	O[C@]1([C@@]2([H])[C@](C[C@](O)(C)[C@@]3(C)C2)([H])OC3=O)CC[C@@]4(O)[C@]5([H])C[C@@H]6[C@]([C@@]7(C)[C@@]5([H])CC[C@@]41C)(O6)[C@@H](O)C=CC7=O	*L. panamensis*	IA	High	2.2	(Cardona et al., 2006a)

Comp[a]	Ori[b]	SMILES[c]	*Leishmania* species[d]	Parasite form[e]	Leishmanicidal Potential[f]	EC_{50} (μM)[g]	Reference
**759**	N	O[C@]1([C@@]2([H])[C@](C[C@](O)(C)[C@@]3(C)C2)([H])OC3=O)CC[C@@]4(O)[C@]5([H])C[C@@H]6[C@]([C@@]7(C)[C@@]5([H])CC[C@@]41C)(O6)[C@@H](OC(C)=O)C=CC7=O	*L. panamensis*	IA	Intermediate	23.5	(Cardona et al., 2006a)
**760**	N	C[C@H](CC1=CC(OC)=C(OC)C(OC)=C1)[C@H](C)CC2=CC(OC)=C(OC)C(OC)=C2	*L. panamensis*	P	Low	234.389	(Sánchez-Suárez et al., 2011)
**760**	N	C[C@H](CC1=CC(OC)=C(OC)C(OC)=C1)[C@H](C)CC2=CC(OC)=C(OC)C(OC)=C2	*L. braziliensis*	P	Low	204.908	(Sánchez-Suárez et al., 2011)
**761**	N	C=CCC1=C[C@@]2(OC)[C@H](C)[C@@H](C3=CC(OCO4)=C4C=C3)[C@H](C1=O)[C@@H]2O	*L. panamensis*	P	N/A		(Sánchez-Suárez et al., 2011)
**761**	N	C=CCC1=C[C@@]2(OC)[C@H](C)[C@@H](C3=CC(OCO4)=C4C=C3)[C@H](C1=O)[C@@H]2O	*L. braziliensis*	P	ND	292.272	(Sánchez-Suárez et al., 2011)
**762**	N	C=CCC1=C[C@@]2(OC)[C@H](C)[C@@H](C3=CC(OC)=C(OC)C=C3)[C@H](C1=O)[C@@H]2O	*L. panamensis*	P	ND	279.191	(Sánchez-Suárez et al., 2011)
**762**	N	C=CCC1=C[C@@]2(OC)[C@H](C)[C@@H](C3=CC(OC)=C(OC)C=C3)[C@H](C1=O)[C@@H]2O	*L. braziliensis*	P	ND	279.191	(Sánchez-Suárez et al., 2011)
**763**	N	CC1=C(C2=CC(OCO3)=C3C=C2)OC4=C1C=C(C=O)C=C4OC	*L. panamensis*	P	ND	322.493	(Sánchez-Suárez et al., 2011)
**763**	N	CC1=C(C2=CC(OCO3)=C3C=C2)OC4=C1C=C(C=O)C=C4OC	*L. braziliensis*	P	ND	322.493	(Sánchez-Suárez et al., 2011)
**764**	N	C[C@H](CC1=CC(OC)=C(OC)C(OC)=C1)[C@@H](C)CC2=CC(OC)=C(OC)C(OC)=C2	*L. panamensis*	P	Low	140.901	(Sánchez-Suárez et al., 2011)
**764**	N	C[C@H](CC1=CC(OC)=C(OC)C(OC)=C1)[C@@H](C)CC2=CC(OC)=C(OC)C(OC)=C2	*L. braziliensis*	P	ND	239.1	(Sánchez-Suárez et al., 2011)
**765**	N	CC1=C(C2=CC(OC)=C(OC)C(OC)=C2)OC3=C1C=C(C=O)C=C3OC	*L. panamensis*	P	ND	280.8	(Sánchez-Suárez et al., 2011)
**765**	N	CC1=C(C2=CC(OC)=C(OC)C(OC)=C2)OC3=C1C=C(C=O)C=C3OC	*L. braziliensis*	P	ND	280.8	(Sánchez-Suárez et al., 2011)
**766**	N	C[C@@H]1[C@@H](C2=CC(OCO3)=C3C=C2)[C@@H]4[C@@H](O)[C@@]1(CC=C)C=C(OC)C4=O	*L. panamensis*	P	Low	99.431	(Sánchez-Suárez et al., 2011)
**766**	N	C[C@@H]1[C@@H](C2=CC(OCO3)=C3C=C2)[C@@H]4[C@@H](O)[C@@]1(CC=C)C=C(OC)C4=O	*L. braziliensis*	P	Low	105.978	(Sánchez-Suárez et al., 2011)
**767**	N	OC1=C(C(CCC2=CC=CC=C2)=O)C(OC)=CC(OC)=C1	*L. panamensis*	P	ND	349.503	(Sánchez-Suárez et al., 2011)
**767**	N	OC1=C(C(CCC2=CC=CC=C2)=O)C(OC)=CC(OC)=C1	*L. braziliensis*	P	ND	349.503	(Sánchez-Suárez et al., 2011)
**768**	N	[H][C@@]12[C@@](C[C@H]3CC2)(CC3=C)CC[C@]4([H])[C@](C)(C(O)=O)CCC[C@]41C	*L. panamensis*	P	ND	165.44	(Sánchez-Suárez et al., 2011)
**768**	N	[H][C@@]12[C@@](C[C@H]3CC2)(CC3=C)CC[C@]4([H])[C@](C)(C(O)=O)CCC[C@]41C	*L. braziliensis*	P	ND	661.76	(Sánchez-Suárez et al., 2011)

Comp[a]	Ori[b]	SMILES[c]	*Leishmania* species[d]	Parasite form[e]	Leishmanicidal Potential[f]	EC_{50} (μM)[g]	Reference
**769**	N	O=C1C=C2[C@]([C@H](C)[C@@H](C3=CC(OC)=C(OC)C(OC)=C3)O2)(OC)C=C1CC=C	*L. panamensis*	P	Low	51.635	(Sánchez-Suárez et al., 2011)
**769**	N	O=C1C=C2[C@]([C@H](C)[C@@H](C3=CC(OC)=C(OC)C(OC)=C3)O2)(OC)C=C1CC=C	*L. braziliensis*	P	Low	64.841	(Sánchez-Suárez et al., 2011)
**770**	N	COC1=CC(CC=C)=CC2=C1OC(C3=CC(OCO4)=C4C=C3)=C2	*L. panamensis*	P	Low	82.277	(Sánchez-Suárez et al., 2011)
**770**	N	COC1=CC(CC=C)=CC2=C1OC(C3=CC(OCO4)=C4C=C3)=C2	*L. braziliensis*	P	Low	147.32	(Sánchez-Suárez et al., 2011)
**771**	N	C=CCC1=C[C@@]2(OC)[C@H](C)[C@@H](C3=CC(OCO4)=C4C(OC)=C3)[C@@](C1=O)(OC)[C@H]2O	*L. panamensis*	P	ND	248.652	(Sánchez-Suárez et al., 2011)
**771**	N	C=CCC1=C[C@@]2(OC)[C@H](C)[C@@H](C3=CC(OCO4)=C4C(OC)=C3)[C@@](C1=O)(OC)[C@H]2O	*L. braziliensis*	P	ND	248.652	(Sánchez-Suárez et al., 2011)
**772**	N	OC1=CC2=C(C=C1OC)C=C(C)C(C)=C2C3=CC=C4OCOC4=C3	*L. panamensis*	P	ND	310.443	(Sánchez-Suárez et al., 2011)
**772**	N	OC1=CC2=C(C=C1OC)C=C(C)C(C)=C2C3=CC=C4OCOC4=C3	*L. braziliensis*	P	ND	310.443	(Sánchez-Suárez et al., 2011)
**773**	N	OC1=CC2=C(C=C1OC)C[C@@H](C)[C@H](C)[C@H]2C3=CC=C4OCOC4=C3	*L. panamensis*	P	ND	306.606	(Sánchez-Suárez et al., 2011)
**773**	N	OC1=CC2=C(C=C1OC)C[C@@H](C)[C@H](C)[C@H]2C3=CC=C4OCOC4=C3	*L. braziliensis*	P	ND	306.606	(Sánchez-Suárez et al., 2011)
**774**	N	COC1=CC2=C(C=C1OC)C(C3=CC=C4OCOC4=C3)=C(C)[C@H](C)C2	*L. panamensis*	P	ND	295.725	(Sánchez-Suárez et al., 2011)
**774**	N	COC1=CC2=C(C=C1OC)C(C3=CC=C4OCOC4=C3)=C(C)[C@H](C)C2	*L. braziliensis*	P	ND	295.725	(Sánchez-Suárez et al., 2011)
**775**	N	O[C@H]1CC[C@@]2(C)C(CC[C@]3(C)C2CC=C4[C@@]3(C)CC[C@]5(C(O[C@@H]6[C@@H]([C@H]([C@@H]([C@@H](CO)O6)O)O)O)=O)C4[C@](C)(O)[C@H](C)CC5)[C@@]1(C)C(O)=O	*L. panamensis*	AA	ND	150.516	(Pérez et al., 2016)
**775**	N	O[C@H]1CC[C@@]2(C)C(CC[C@]3(C)C2CC=C4[C@@]3(C)CC[C@]5(C(O[C@@H]6[C@@H]([C@H]([C@@H]([C@@H](CO)O6)O)O)O)=O)C4[C@](C)(O)[C@H](C)CC5)[C@@]1(C)C(O)=O	*L. panamensis*	IA	Low	62.615	(Pérez et al., 2016)
**776**	N	O[C@H]1CC[C@@]2(C)C(CC[C@]3(C)C2CC=C4[C@@]3(C)CC[C@]5(C(O[C@@H]6[C@@H]([C@H]([C@@H]([C@@H](CO)O6)O)O)O)=O)C4[C@](C)(O)[C@H](C)CC5)[C@@]1(C)CO	*L. panamensis*	AA	ND	153.751	(Pérez et al., 2016)
**776**	N	O[C@H]1CC[C@@]2(C)C(CC[C@]3(C)C2CC=C4[C@@]3(C)CC[C@]5(C(O[C@@H]6[C@@H]([C@H]([C@@H]([C@@H](CO)O6)O)O)O)=O)C4[C@](C)(O)[C@H](C)CC5)[C@@]1(C)CO	*L. panamensis*	IA	High	9.071	(Pérez et al., 2016)
**777**	N	C[C@@]12C(CC[C@]3(C)C2CC=C4[C@@]3(C)CC[C@]5(C(O)=O)C4CC(C)(C)CC5)[C@@](CO)(C)[C@@H](O[C@H]6[C@@H]([C@H]([C@H](C	*L. panamensis*	AA	High	8.898	(Correa et al., 2014)

Comp[a]	Ori[b]	SMILES[c]	*Leishmania* species[d]	Parasite form[e]	Leishmanicidal Potential[f]	EC_{50} (μM)[g]	Reference
		O6)O)O)O[C@H]7[C@@H]([C@@H]([C@H]([C@H](C)O7)O)O[C@H]8[C@@H]([C@H]([C@@H](CO8)OC(C)=O)OC(C)=O)O)O)CC1					
**777**	N	C[C@@]12C(CC[C@]3(C)C2CC=C4[C@@]3(C)CC[C@]5(C(O)=O)C4CC(C)(C)CC5)[C@@](CO)(C)[C@@H](O[C@H]6[C@@H]([C@H]([C@H](CO6)O)O)O[C@H]7[C@@H]([C@@H]([C@H]([C@H](C)O7)O)O[C@H]8[C@@H]([C@H]([C@@H](CO8)OC(C)=O)OC(C)=O)O)O)CC1	*L. panamensis*	IA	High	2.587	(Correa et al., 2014)
**778**	N	C[C@@]12C(CC[C@]3(C)C2CC=C4[C@@]3(C)CC[C@]5(C(O)=O)C4CC(C)(C)CC5)[C@@](CO)(C)[C@@H](O[C@H]6[C@@H]([C@H]([C@H](CO6)O)O)O[C@H]7[C@@H]([C@@H]([C@H]([C@H](C)O7)O)O[C@H]8[C@@H]([C@H]([C@H](CO8)OC(C)=O)OC(C)=O)O)O)CC1	*L. panamensis*	AA	Intermediate	17.175	(Correa et al., 2014)
**778**	N	C[C@@]12C(CC[C@]3(C)C2CC=C4[C@@]3(C)CC[C@]5(C(O)=O)C4CC(C)(C)CC5)[C@@](CO)(C)[C@@H](O[C@H]6[C@@H]([C@H]([C@H](CO6)O)O)O[C@H]7[C@@H]([C@@H]([C@H]([C@H](C)O7)O)O[C@H]8[C@@H]([C@H]([C@H](CO8)OC(C)=O)OC(C)=O)O)O)CC1	*L. panamensis*	IA	High	2.794	(Correa et al., 2014)
**779**	N	C[C@@]12C(CC[C@]3(C)C2CC=C4[C@@]3(C)CC[C@]5(C(O)=O)C4CC(C)(C)CC5)[C@@](CO)(C)[C@@H](O[C@H]6[C@@H]([C@H]([C@H](CO6)O)O)O[C@H]7[C@@H]([C@@H]([C@H]([C@H](C)O7)O)O[C@H]8[C@@H]([C@H]([C@@H](CO8)OC(C)=O)O)O)O)CC1	*L. panamensis*	AA	Intermediate	11.682	(Correa et al., 2014)
**779**	N	C[C@@]12C(CC[C@]3(C)C2CC=C4[C@@]3(C)CC[C@]5(C(O)=O)C4CC(C)(C)CC5)[C@@](CO)(C)[C@@H](O[C@H]6[C@@H]([C@H]([C@H](CO6)O)O)O[C@H]7[C@@H]([C@@H]([C@H]([C@H](C)O7)O)O[C@H]8[C@@H]([C@H]([C@@H](CO8)OC(C)=O)O)O)O)CC1	*L. panamensis*	IA	High	2.271	(Correa et al., 2014)
**780**	N	O[C@H]1CC[C@@]2(C)C(CC[C@]3(C)C2CC=C4[C@@]3(C)CC[C@]5(C(O)=O)C4CC(C)(C)CC5)[C@]1(C)CO	*L. panamensis*	AA	Low	38.319	(Correa et al., 2014)
**780**	N	O[C@H]1CC[C@@]2(C)C(CC[C@]3(C)C2CC=C4[C@@]3(C)CC[C@]5(C(O)=O)C4CC(C)(C)CC5)[C@]1(C)CO	*L. panamensis*	IA	Intermediate	22.652	(Correa et al., 2014)
**781**	S	O=C(/C=C/C1=CC=CC=C1)C2=CC=C(CC(C)=O)C=C2	*L. panamensis*	P	Low	55.7	(Pacheco et al., 2013)
**782**	S	O=C(/C=C/C1=CC=C(C)C=C1)C2=CC=C(CC(C)=O)C=C2	*L. panamensis*	P	ND	715.99	(Pacheco et al., 2013)
**783**	S	O=C(/C=C/C1=CC=C(Cl)C=C1)C2=CC=C(CC(C)=O)C=C2	*L. panamensis*	P	Low	49.7	(Pacheco et al., 2013)
**784**	S	O=C(/C=C/C1=CC=C(F)C=C1)C2=CC=C(CC(C)=O)C=C2	*L. panamensis*	P	ND	706	(Pacheco et al., 2013)
**785**	S	O=C(/C=C/C1=CC=C([N+]([O-])=O)C=C1)C2=CC=C(CC(C)=O)C=C2	*L. panamensis*	P	ND	644.5	(Pacheco et al., 2013)
**786**	S	O=C(/C=C/C1=CC=C(OC)C=C1)C2=CC=C(CC(C)=O)C=C2	*L. panamensis*	P	ND	677.2	(Pacheco et al., 2013)
**787**	S	O=C(/C=C/C1=CC=C(OCO2)C2=C1)C3=CC=C(CC(C)=O)C=C3	*L. panamensis*	P	N/A		(Pacheco et al., 2013)
**788**	S	O=C(/C=C/C1=CC(OC)=C(OC)C(OC)=C1)C2=CC=C(CC(C)=O)C=C2	*L. panamensis*	P	N/A		(Pacheco et al., 2013)
**789**	S	O=C(/C=C/C1=CC=CN1)C2=CC=C(CC(C)=O)C=C2	*L. panamensis*	P	ND	592.3	(Pacheco et al., 2013)
**790**	S	O=C(/C=C/C1=CC=CO1)C2=CC=C(CC(C)=O)C=C2	*L. panamensis*	P	ND	783.5	(Pacheco et al., 2013)
**791**	S	O=C(/C=C/C1=CC=CS1)C2=CC=C(CC(C)=O)C=C2	*L. panamensis*	P	ND	737.1	(Pacheco et al., 2013)
**792**	S	[O-][N+]1=C(C#N)C(CC(C2CC2)=O)=[N+]([O-])C3=CC=CC=C31	*L. infantum*	AA	High	3.6	(Barea et al., 2013)
**792**	S	[O-][N+]1=C(C#N)C(CC(C2CC2)=O)=[N+]([O-])C3=CC=CC=C31	*L. amazonensis*	AA	N/A		(Barea et al., 2013)

Comp[a]	Ori[b]	SMILES[c]	*Leishmania* species[d]	Parasite form[e]	Leishmanicidal Potential[f]	EC_{50} (μM)[g]	Reference
**793**	S	[O-][N+]1=C(C#N)C(CC(C2CC2)=O)=[N+]([O-])C3=CC=C(Cl)C=C31	*L. infantum*	AA	High	3.5	(Barea et al., 2013)
**793**	S	[O-][N+]1=C(C#N)C(CC(C2CC2)=O)=[N+]([O-])C3=CC=C(Cl)C=C31	*L. amazonensis*	AA	N/A		(Barea et al., 2013)
**794**	S	[O-][N+]1=C(C#N)C(CC(C2CC2)=O)=[N+]([O-])C3=CC=C(C)C=C31	*L. infantum*	AA	High	3.5	(Barea et al., 2013)
**794**	S	[O-][N+]1=C(C#N)C(CC(C2CC2)=O)=[N+]([O-])C3=CC=C(C)C=C31	*L. amazonensis*	AA	N/A		(Barea et al., 2013)
**795**	S	[O-][N+]1=C(C#N)C(CC(C2CC2)=O)=[N+]([O-])C3=CC=C(OC)C=C31	*L. infantum*	AA	High	3.9	(Barea et al., 2013)
**795**	S	[O-][N+]1=C(C#N)C(CC(C2CC2)=O)=[N+]([O-])C3=CC=C(OC)C=C31	*L. amazonensis*	AA	N/A		(Barea et al., 2013)
**796**	S	[O-][N+]1=C(C#N)C(CC(C2CCCC2)=O)=[N+]([O-])C3=CC=C(Cl)C=C31	*L. infantum*	AA	N/A		(Barea et al., 2013)
**796**	S	[O-][N+]1=C(C#N)C(CC(C2CCCC2)=O)=[N+]([O-])C3=CC=C(Cl)C=C31	*L. amazonensis*	AA	Intermediate	14.9	(Barea et al., 2013)
**797**	S	[O-][N+]1=C(C#N)C(CC(C2CCCCC2)=O)=[N+]([O-])C3=CC=C(Cl)C=C31	*L. infantum*	AA	High	2.5	(Barea et al., 2013)
**797**	S	[O-][N+]1=C(C#N)C(CC(C2CCCCC2)=O)=[N+]([O-])C3=CC=C(Cl)C=C31	*L. amazonensis*	AA	N/A		(Barea et al., 2013)
**798**	S	[O-][N+]1=C(C#N)C(CC(C2CCCCC2)=O)=[N+]([O-])C3=CC=C(C)C=C31	*L. infantum*	AA	High	4.6	(Barea et al., 2013)
**798**	S	[O-][N+]1=C(C#N)C(CC(C2CCCCC2)=O)=[N+]([O-])C3=CC=C(C)C=C31	*L. amazonensis*	AA	N/A		(Barea et al., 2013)
**799**	S	[O-][N+]1=C(C#N)C(CC(C2CCCCC2)=O)=[N+]([O-])C3=CC=C(OC)C=C31	*L. infantum*	AA	High	3.4	(Barea et al., 2013)
**799**	S	[O-][N+]1=C(C#N)C(CC(C2CCCCC2)=O)=[N+]([O-])C3=CC=C(OC)C=C31	*L. amazonensis*	AA	N/A		(Barea et al., 2013)
**800**	S	[O-][N+]1=C(C#N)C(CC(C)=O)=[N+]([O-])C2=CC=CC=C21	*L. infantum*	AA	N/A		(Barea et al., 2013)
**800**	S	[O-][N+]1=C(C#N)C(CC(C)=O)=[N+]([O-])C2=CC=CC=C21	*L. amazonensis*	AA	Intermediate	16.6	(Barea et al., 2013)
**801**	S	[O-][N+]1=C(C#N)C(CC(CC(C)=O)=O)=[N+]([O-])C2=CC=CC=C21	*L. infantum*	AA	N/A		(Barea et al., 2013)
**801**	S	[O-][N+]1=C(C#N)C(CC(CC(C)=O)=O)=[N+]([O-])C2=CC=CC=C21	*L. amazonensis*	AA	Intermediate	11.9	(Barea et al., 2013)
**802**	S	[O-][N+]1=C(C#N)C(CC(CC(C)=O)=O)=[N+]([O-])C2=CC=C(Cl)C=C21	*L. infantum*	AA	N/A		(Barea et al., 2013)
**802**	S	[O-][N+]1=C(C#N)C(CC(CC(C)=O)=O)=[N+]([O-])C2=CC=C(Cl)C=C21	*L. amazonensis*	AA	High	4	(Barea et al., 2013)
**803**	S	[O-][N+]1=C(C#N)C(CC(CCCCl)=O)=[N+]([O-])C2=CC=C(Cl)C=C21	*L. infantum*	AA	N/A		(Barea et al., 2013)
**803**	S	[O-][N+]1=C(C#N)C(CC(CCCCl)=O)=[N+]([O-])C2=CC=C(Cl)C=C21	*L. amazonensis*	AA	High	0.7	(Barea et al., 2013)
**804**	S	CCC1=C2/C=C3C(C)=C(CC)C(/C=C(N/4)/C(CC)=C(C)C4=C(N/5)\C(C)=C(CC)C5=C\C6=[NH+]/C(C(C)=C6CC)=C\C(N2)=C1CC)=[NH+]/3	*L. panamensis*	AA	High	7.8	(Hooker et al., 2012)
**804**	S	CCC1=C2/C=C3C(C)=C(CC)C(/C=C(N/4)/C(CC)=C(C)C4=C(N/5)\C(C)=C(CC)C5=C\C6=[NH+]/C(C(C)=C6CC)=C\C(N2)=C1CC)=[NH+]/3	*L. panamensis*	IA	High	0.6	(Hooker et al., 2012)

Comp[a]	Ori[b]	SMILES[c]	*Leishmania* species[d]	Parasite form[e]	Leishmanicidal Potential[f]	EC_{50} (μM)[g]	Reference
**805**	S	CC1=C(CC)C2=[NH+]/C1=C\C(O3)=CC=C3/C=C4C(C)=C(CC)C(/C=C(N/5)/C(CC)=C(C)C5=C(N/6)\C(C)=C(CC)C6=C\2)=[NH+]/4	*L. panamensis*	AA	High	7.7	(Hooker et al., 2012)
**805**	S	CC1=C(CC)C2=[NH+]/C1=C\C(O3)=CC=C3/C=C4C(C)=C(CC)C(/C=C(N/5)/C(CC)=C(C)C5=C(N/6)\C(C)=C(CC)C6=C\2)=[NH+]/4	*L. panamensis*	IA	High	2.9	(Hooker et al., 2012)
**806**	S	CC1=C(CC)C2=[NH+]/C1=C\C(S3)=CC=C3/C=C4C(C)=C(CC)C(/C=C(N/5)/C(CC)=C(C)C5=C(N/6)\C(C)=C(CC)C6=C\2)=[NH+]/4	*L. panamensis*	AA	Intermediate	17.1	(Hooker et al., 2012)
**806**	S	CC1=C(CC)C2=[NH+]/C1=C\C(S3)=CC=C3/C=C4C(C)=C(CC)C(/C=C(N/5)/C(CC)=C(C)C5=C(N/6)\C(C)=C(CC)C6=C\2)=[NH+]/4	*L. panamensis*	IA	High	5.5	(Hooker et al., 2012)
**807**	S	CC1=C2/C=C3C4=C(C=CC=C4)C(/C=C5C(C)=C(CC)C(/C=C(N/6)/C(CC)=C(C)C6=C(N/7)\C(C)=C(CC)C7=C\C(N2)=C1CC)=[NH+]/5)=C/3	*L. panamensis*	AA	Intermediate	13.7	(Hooker et al., 2012)
**807**	S	CC1=C2/C=C3C4=C(C=CC=C4)C(/C=C5C(C)=C(CC)C(/C=C(N/6)/C(CC)=C(C)C6=C(N/7)\C(C)=C(CC)C7=C\C(N2)=C1CC)=[NH+]/5)=C/3	*L. panamensis*	IA	High	9.7	(Hooker et al., 2012)
**808**	S	CC1=C(CC)C2=[NH+]/C1=C\C(N3)=C(C4=CC=CC=C4C5=C6C=CC=C5)C6=C3/C=C7C(C)=C(CC)C(/C=C(N/8)/C(CC)=C(C)C8=C(N/9)\C(C)=C(CC)C9=C\2)=[NH+]/7	*L. panamensis*	AA	Low	145	(Hooker et al., 2012)
**808**	S	CC1=C(CC)C2=[NH+]/C1=C\C(N3)=C(C4=CC=CC=C4C5=C6C=CC=C5)C6=C3/C=C7C(C)=C(CC)C(/C=C(N/8)/C(CC)=C(C)C8=C(N/9)\C(C)=C(CC)C9=C\2)=[NH+]/7	*L. panamensis*	IA	Low	121	(Hooker et al., 2012)
**809**	S	CC1=C(CC)C2=[NH+]/C1=C\C(N3)=C(C4=C(C5=CC=C6)C6=CC=C4)C5=C3/C=C7C(C)=C(CC)C(/C=C(N/8)/C(CC)=C(C)C8=C(N/9)\C(C)=C(CC)C9=C\2)=[NH+]/7	*L. panamensis*	AA	Low	101	(Hooker et al., 2012)
**809**	S	CC1=C(CC)C2=[NH+]/C1=C\C(N3)=C(C4=C(C5=CC=C6)C6=CC=C4)C5=C3/C=C7C(C)=C(CC)C(/C=C(N/8)/C(CC)=C(C)C8=C(N/9)\C(C)=C(CC)C9=C\2)=[NH+]/7	*L. panamensis*	IA	Low	153	(Hooker et al., 2012)
**810**	S	NC(=O)N\N=C\C1=CC=CC=C1O	*L. panamensis*	IA	ND	213.68	(Benítez et al., 2011)
**810**	S	NC(=O)N\N=C\C1=CC=CC=C1O	*L. chagasi*	IA	High	6.67	(Benítez et al., 2011)
**810**	S	NC(=O)N\N=C\C1=CC=CC=C1O	*L. panamensis*	P	Intermediate	13.63	(Benítez et al., 2011)
**810**	S	NC(=O)N\N=C\C1=CC=CC=C1O	*L. chagasi*	P	High	3.12	(Benítez et al., 2011)
**811**	S	NC(=O)N\N=C\C1=CC(Br)=CC=C1O	*L. panamensis*	IA	Intermediate	10.46	(Benítez et al., 2011)
**811**	S	NC(=O)N\N=C\C1=CC(Br)=CC=C1O	*L. chagasi*	IA	Intermediate	13.41	(Benítez et al., 2011)
**811**	S	NC(=O)N\N=C\C1=CC(Br)=CC=C1O	*L. panamensis*	P	High	8.7	(Benítez et al., 2011)
**811**	S	NC(=O)N\N=C\C1=CC(Br)=CC=C1O	*L. chagasi*	P	High	3.47	(Benítez et al., 2011)
**812**	S	COC1=C(O)C(\C=N\NC(N)=O)=CC=C1	*L. panamensis*	IA	ND	558.66	(Benítez et al., 2011)
**812**	S	COC1=C(O)C(\C=N\NC(N)=O)=CC=C1	*L. chagasi*	IA	ND	558.66	(Benítez et al., 2011)
**812**	S	COC1=C(O)C(\C=N\NC(N)=O)=CC=C1	*L. panamensis*	P	ND	558.66	(Benítez et al., 2011)
**812**	S	COC1=C(O)C(\C=N\NC(N)=O)=CC=C1	*L. chagasi*	P	Low	517.99	(Benítez et al., 2011)
**813**	S	CCOC1=C(O)C(\C=N\NC(N)=O)=CC=C1	*L. panamensis*	IA	ND	109.9	(Benítez et al., 2011)
**813**	S	CCOC1=C(O)C(\C=N\NC(N)=O)=CC=C1	*L. chagasi*	IA	ND	109.9	(Benítez et al., 2011)
**813**	S	CCOC1=C(O)C(\C=N\NC(N)=O)=CC=C1	*L. panamensis*	P	Low	78.91	(Benítez et al., 2011)

Comp[a]	Ori[b]	SMILES[c]	*Leishmania* species[d]	Parasite form[e]	Leishmanicidal Potential[f]	EC_{50} (μM)[g]	Reference
**813**	S	CCOC1=C(O)C(\C=N\NC(N)=O)=CC=C1	*L. chagasi*	P	Low	266.9	(Benítez et al., 2011)
**814**	S	COC1=C(O)C(\C=N\NC(N)=O)=CC(Br)=C1	*L. panamensis*	IA	Low	33.4	(Benítez et al., 2011)
**814**	S	COC1=C(O)C(\C=N\NC(N)=O)=CC(Br)=C1	*L. chagasi*	IA	High	6.93	(Benítez et al., 2011)
**814**	S	COC1=C(O)C(\C=N\NC(N)=O)=CC(Br)=C1	*L. panamensis*	P	High	3.18	(Benítez et al., 2011)
**814**	S	COC1=C(O)C(\C=N\NC(N)=O)=CC(Br)=C1	*L. chagasi*	P	High	8.89	(Benítez et al., 2011)
**815**	N	C=C1[C@@H]2[C@H](C(C)(C)C2)CC/C(C)=C/CC1	*L. infantum*	P	Intermediate	24.02	(Leal et al., 2013b)
**816**	N	CC1=CCC2C(C)(C)C1C2	*L. infantum*	P	Low	45.94	(Leal et al., 2013b)
**817**	N	C/C(C)=C\CCC(O)(C)C=C	*L. infantum*	P	ND	>100	(Leal et al., 2013b)
**818**	N	CC(C1CCC(C)=CC1)=C	*L. infantum*	P	ND	>100	(Leal et al., 2013b)
**819**	N	CC1(C2CCC(O1)(CC2)C)C	*L. infantum*	P	ND	>100	(Leal et al., 2013b)
**815**	N	C=C1[C@@H]2[C@H](C(C)(C)C2)CC/C(C)=C/CC1	*L. infantum*	IA	Low	53.39	(Leal et al., 2013b)
**816**	N	CC1=CCC2C(C)(C)C1C2	*L. infantum*	IA	ND	>100	(Leal et al., 2013b)
**817**	N	C/C(C)=C\CCC(O)(C)C=C	*L. infantum*	IA	ND	>100	(Leal et al., 2013b)
**818**	N	CC(C1CCC(C)=CC1)=C	*L. infantum*	IA	ND	>100	(Leal et al., 2013b)
**819**	N	CC1(C2CCC(O1)(CC2)C)C	*L. infantum*	IA	ND	>100	(Leal et al., 2013b)
**820**	N	C/C1=C\C[C@H](OC(C)=O)/C(C)=C/[C@@H](OC(C2=C)=O)[C@H]2[C@H](OC(/C(CO)=C/CO)=O)C1	*L. donovani*	AA	Low	39	(Mokoka et al., 2013)
**821**	N	C/C1=C\C[C@H](OC(C(O)C(C)C)=O)/C(C)=C/[C@@H](OC(C2=C)=O)[C@H]2[C@H](OC(/C(CO)=C/CO)=O)C1	*L. donovani*	AA	Low	65	(Mokoka et al., 2013)
**822**	N	C=C([C@]1([H])C[C@@H]2O)C[C@@H](OC(C(CO)=C)=O)[C@@H]3[C@H](OC(C3=C)=O)[C@@]1([H])C2=C	*L. donovani*	AA	High	1.56	(Mokoka et al., 2013)
**823**	N	C/C(C)=C\CC/C(C)=C/C=O	*L. chagasi*	P	Low	40.3	(Escobar et al., 2010)
**824**	N	C=C([C@H]1CCC(C)=CC1)C	*L. chagasi*	P	Low	129.1	(Escobar et al., 2010)
**825**	N	C=C([C@@H]1CCC(C)=CC1)C	*L. chagasi*	P	Low	261	(Escobar et al., 2010)
**826**	N	CC1=C(O)C=C(C(C)C)C=C1	*L. chagasi*	P	Low	28	(Escobar et al., 2010)
**827**	N	CC1=CC=C(C(C)C)C=C1	*L. chagasi*	P	Low	149.1	(Escobar et al., 2010)
**828**	N	CC1=CCC(C(C)C)=CC1	*L. chagasi*	P	Low	145.1	(Escobar et al., 2010)
**829**	N	CC([C@@H]1CC(C(C)=CC1)=O)=C	*L. chagasi*	P	ND	>300	(Escobar et al., 2010)
**830**	N	CC([C@H]1CC(C(C)=CC1)=O)=C	*L. chagasi*	P	Low	179.1	(Escobar et al., 2010)
**133**	N	CC1=CC(O)=C(C(C)C)C=C1	*L. chagasi*	P	Low	65.2	(Escobar et al., 2010)
**823**	N	C/C(C)=C\CC/C(C)=C/C=O	*L. chagasi*	IA	ND	>30	(Escobar et al., 2010)
**824**	N	C=C([C@H]1CCC(C)=CC1)C	*L. chagasi*	IA	ND	>30	(Escobar et al., 2010)
**825**	N	C=C([C@@H]1CCC(C)=CC1)C	*L. chagasi*	IA	ND	>30	(Escobar et al., 2010)
**826**	N	CC1=C(O)C=C(C(C)C)C=C1	*L. chagasi*	IA	ND	>30	(Escobar et al., 2010)
**827**	N	CC1=CC=C(C(C)C)C=C1	*L. chagasi*	IA	ND	>30	(Escobar et al., 2010)
**828**	N	CC1=CCC(C(C)C)=CC1	*L. chagasi*	IA	ND	>30	(Escobar et al., 2010)
**829**	N	CC([C@@H]1CC(C(C)=CC1)=O)=C	*L. chagasi*	IA	ND	>30	(Escobar et al., 2010)
**830**	N	CC([C@H]1CC(C(C)=CC1)=O)=C	*L. chagasi*	IA	ND	>30	(Escobar et al., 2010)

Comp[a]	Ori[b]	SMILES[c]	*Leishmania* species[d]	Parasite form[e]	Leishmanicidal Potential[f]	EC_{50} (μM)[g]	Reference
**133**	N	CC1=CC(O)=C(C(C)C)C=C1	*L. chagasi*	IA	ND	>30	(Escobar et al., 2010)
**831**	N	CC(C1=C(C=C2)C3=C(C=C(C)C(O)=C3)C=C1)(C)C2=O	*L. donovani*	AA	Intermediate	14	(Hata et al., 2014)
**832**	N	CC(C1=C(C=C2)C3=C(C=C(C)C(OC4=C(C(C=CC(C5(C)C)=O)=C5C=C6)C6=CC(C)=C4O)=C3)C=C1)(C)C2=O	*L. donovani*	AA	N/A		(Hata et al., 2014)
**833**	N	CC1(C)CC[C@]2(C(O)=O)CC[C@@]3(C)[C@]4(C)CC[C@@]5([H])C(C)(C)[C@@H](O[C@H]6[C@@H]([C@H]([C@@H]([C@@H](C(O)=O)O6)O)O[C@H]7[C@@H]([C@@H]([C@H]([C@H](C)O7)O)O)O)O)CC[C@]5(C)[C@@]4([H])CC=C3[C@]2([H])C1	*L. donovani*	AA	High	7.8	(Hata et al., 2014)
**834**	S	CCC(C=C1)=CC=C1/C(C(C=C/2)=NC2=C3\C4=CC=C(CC)C=C4)=C5N/C(C=C/5)=C(C6=CC=C(CC)C=C6)\C7=N/C(C=C7)=C(C8=CC=C(CC)C=C8)\C9=CC=C3N9	*L. braziliensis*	P	Low	34.1	(Espitia-Almeida et al., 2020)
**835**	S	CCC(C=C1)=CC=C1/C(C(C=C/2)=NC2=C3\C4=CC=C(CC)C=C4)=C5N/C(C=C/5)=C(C6=CC=C(CC)C=C6)\C7=N/C(C=C7)=C(C8=CC=C(CC)C=C8)\C9=CC=C3N10	*L. panamensis*	P	Intermediate	20.6	(Espitia-Almeida et al., 2020)
**836**	N	C[C@@H](/C=C/[C@H](C)C(C)C)[C@H]1CCC2[C@@]34C=C[C@@]5(OO4)C[C@@H](C)CC[C@]5(C)C3CC[C@@]21C	*L. panamensis*	IA	High	4.1	(Correa et al., 2006)

[a] Compounds compiled from 75 reviewed articles. 836 compounds were retrieved along with their reported leishmanicidal activity.

[b] Origin. The compounds were classified between "synthetic" (S) and "natural" (N).

[c] Simplified Molecular-Input Line-Entry System (SMILES) is a line notation for chemical structure. The notation uses the American Standard Code for Information Interchange (ASCII) character encoding (Weininger, 1988).

[d] *Leishmania* species against the leishmanicidal activity was assayed.

[e] Parasite form involved in the leishmanicidal assay: intracellular amastigotes (IA), axenic amastigotes (AA) and promastigotes (P).

[f] Leishmanicidal potential was classified as categories according to the resulting pEC_{50} values for each compound: High = $pEC_{50} > 5.00$ ($EC_{50} < 10.0$ μM); Intermediate = $4.60 < pEC_{50} < 4.99$ (25.1 μM > EC_{50} > 10.0 μM); Low = $pEC_{50} < 4.6$ ($EC_{50} > 25.1$ μM); Not Determined = compounds included into the respective study, but the EC_{50} value was over the maximum evaluated concentration; Not Avalilable = compounds included into the respective study, but the antileishmanial assay did not return an EC_{50}.

[g] Half maximal effective concentration (EC_{50}). Concentration that shows the 50% of the maximal response.

References

Alvarez, N., Robledo, S., Velez, I. D., Robert, J. M., Le Baut, G., and Le Pape, P. (2002). Inhibition of parasite protein kinase C by new antileishmanial imidazolidin-2-one compounds. *J. Enzyme Inhib. Med. Chem.* 17, 443–447. doi:10.1080/1475636021000005749.

Arango, V., Domínguez, J. J., Cardona, W., Robledo, S. M., Muñoz, D. L., Figadere, B., et al. (2012). Synthesis and leishmanicidal activity of quinoline-triclosan and quinoline-eugenol hybrids. *Med. Chem. Res.* 21, 3445–3454. doi:10.1007/s00044-011-9886-8.

Arango, V., Robledo, S., Séon-Méniel, B., Figadère, B., Cardona, W., Sáez, J., et al. (2010). Coumarins from Galipea panamensis and Their Activity against Leishmania panamensis. *J. Nat. Prod.* 73, 1012–1014. doi:10.1021/np100146y.

Barea, C., Pabón, A., Castillo, D., Zimic, M., Quiliano, M., Galiano, S., et al. (2011). New salicylamide and sulfonamide derivatives of quinoxaline 1,4-di-N-oxide with antileishmanial and antimalarial activities. *Bioorganic Med. Chem. Lett.* 21, 4498–4502. doi:10.1016/j.bmcl.2011.05.125.

Barea, C., Pabón, A., Galiano, S., Pérez-Silanes, S., Gonzalez, G., Deyssard, C., et al. (2012). Antiplasmodial and leishmanicidal activities of 2-cyano-3-(4- phenylpiperazine-1-carboxamido) quinoxaline 1,4-dioxide derivatives. *Molecules* 17, 9451–9461. doi:10.3390/molecules17089451.

Barea, C., Pabón, A., Pérez-Silanes, S., Galiano, S., Gonzalez, G., Monge, A., et al. (2013). New amide derivatives of quinoxaline 1,4-di-N-oxide with leishmanicidal and antiplasmodial

activities. *Molecules* 18, 4718–4727. doi:10.3390/molecules18044718.

Benítez, J., Becco, L., Correia, I., Leal, S. M., Guiset, H., Pessoa, J. C., et al. (2011). Vanadium polypyridyl compounds as potential antiparasitic and antitumoral agents: New achievements. *J. Inorg. Biochem.* 105, 303–312. doi:10.1016/j.jinorgbio.2010.11.001.

Blanco, M. C., Escobar, P., Leal, S. M., Bahsas, A., Cobo, J., Nogueras, M., et al. (2014). Synthesis of novel polysubstituted (2SR,4RS)-2-heteroaryltetrahydro-1,4- epoxy-1-benzazepines and cis-2-heteroaryl-4-hydroxytetrahydro-1H-1-benzazepines as antiparasitic agents. *Eur. J. Med. Chem.* 86, 291–309. doi:10.1016/j.ejmech.2014.08.055.

Bohórquez, A. R. R., Rivero, P. E., Leal, S. M., and Kouznetsov, V. V (2012). In vitro activity against Trypanosoma cruzi and Leishmania chagasi parasites of 2,4-diaryl 1,2,3,4-tetrahydroquinoline derivatives. *Lett. Drug Des. Discov.* 9, 802–808. doi:10.2174/157018012802652994.

Bompart, D., Núñez-Durán, J., Rodríguez, D., Kouznetsov, V. V, Meléndez Gómez, C. M., Sojo, F., et al. (2013). Anti-leishmanial evaluation of C2-aryl quinolines: Mechanistic insight on bioenergetics and sterol biosynthetic pathway of Leishmania braziliensis. *Bioorganic Med. Chem.* 21, 4426–4431. doi:10.1016/j.bmc.2013.04.063.

Cardona, D., Quiñones, W., Torres, F., Robledo, S., Vélez, I. D., Cruz, V., et al. (2006a). Leishmanicidal activity of withajardins and acnistins. An experimental and computational study. *Tetrahedron* 62, 6822–6829. doi:10.1016/j.tet.2006.04.101.

Cardona G., W., Quiñones F., W., and Echeverri L., F. (2004). Leishmanicidal activity of passifloricin A and derivatives. *Molecules* 9, 666–672. doi: 10.3390/90800666.

Cardona, W., Arango, V., Domínguez, J. J., Robledo, S. M., Muñoz, D. L., Figadere, B., et al. (2013). Synthesis and leishmanicidal activity of new bis-alkylquinolines. *J. Chil. Chem. Soc.* 58, 1709–1712. doi:10.4067/S0717-97072013000200014.

Cardona, W., Quiñones, W., Robledo, S., Vélez, I. D., Murga, J., García-Fortanet, J., et al. (2006b). Antiparasite and antimycobacterial activity of passifloricin analogues. *Tetrahedron* 62, 4086–4092. doi:10.1016/j.tet.2006.02.017.

Carmona, D., Sáez, J., Granados, H., Pérez, E., Blair, S., Angulo, A., et al. (2003). Antiprotozoal 6-substituted-5,6-dihydro-α-pyrones from Raimondia cf. Monoica. *Nat. Prod. Res.* 17, 275–280. doi:10.1080/1057563031000065062.

Castano, M., Cardona, W., Quinones, W., Robledo, S., and Echeverri, F. (2009). Leishmanicidal Activity of Aliphatic and Aromatic Lactones: Correlation Structure-Activity. *Molecules* 14, 2491–2500. doi:10.3390/molecules14072491.

Coa, J. C., Castrillón, W., Cardona, W., Carda, M., Ospina, V., Muñoz, J. A., et al. (2015). Synthesis, leishmanicidal, trypanocidal and cytotoxic activity of quinoline-hydrazone hybrids. *Eur. J. Med. Chem.* 101, 746–753. doi:10.1016/j.ejmech.2015.07.018.

Coa, J. C., García, E., Carda, M., Agut, R., Vélez, I. D., Muñoz, J. A., et al. (2017). Synthesis, leishmanicidal, trypanocidal and cytotoxic activities of quinoline-chalcone and quinoline-chromone hybrids. *Med. Chem. Res.* 26, 1405–1414. doi:10.1007/s00044-017-1846-5.

Coa, J. C., Yepes, A., Carda, M., Conesa-Milián, L., Upegui, Y., Robledo, S. M., et al. (2020). Synthesis, In Silico Studies, Antiprotozoal and Cytotoxic Activities of Quinoline-Biphenyl Hybrids. *ChemistrySelect* 5, 2918–2924. doi:10.1002/slct.201903835.

Correa, E., Cardona, D., Quiñones, W., Torres, F., Franco, A. E., Vélez, I. D., et al. (2006). Leishmanicidal activity of Pycnoporus sanguineus. *Phytother. Res.* 20, 497–499. doi:10.1002/ptr.1890.

Correa, E., Quiñones, W., Robledo, S., Carrillo, L., Archbold, R., Torres, F., et al. (2014). Leishmanicidal and trypanocidal activity of sapindus saponaria . *Bol. Latinoam. y del Caribe Plantas Med. y Aromat.* 13, 311–323. Available at: https://www.scopus.com/inward/record.uri?eid=2-s2.0-84905590650&partnerID=40&md5=7bf6aeef4fce99a6788b8667f0041ada.

Coy Barrera, C. A., Coy Barrera, E. D., Granados Falla, D. S., Delgado Murcia, G., and Cuca Suarez, L. E. (2011). seco-limonoids and quinoline alkaloids from Raputia heptaphylla and their antileishmanial activity. *Chem. Pharm. Bull. (Tokyo).* 59, 855–859. doi:10.1248/cpb.59.855.

Cuartas, V., Robledo, S. M., Vélez, I. D., Crespo, M. D. P., Sortino, M., Zacchino, S., et al. (2020). New thiazolyl-pyrazoline derivatives bearing nitrogen mustard as potential antimicrobial and antiprotozoal agents. *Arch. Pharm. (Weinheim).* 353, e1900351. doi:10.1002/ardp.201900351.

Duque-Benítez, S. M., Ríos-Vásquez, L. A., Ocampo-Cardona, R., Cedeño, D. L., Jones, M. A., Vélez, I. D., et al. (2016). Synthesis of Novel Quaternary Ammonium Salts and Their in Vitro Antileishmanial Activity and U-937 Cell Cytotoxicity. *Molecules* 21, 1–16. doi:10.3390/molecules21040381.

Escobar, P., Leal, S. M., Herrera, L. V, Martinez, J. R., and Stashenko, E. (2010). Chemical composition and antiprotozoal activities of Colombian Lippia spp essential oils and their major components. *Mem. Inst. Oswaldo Cruz* 105, 184–190. doi:10.1590/S0074-02762010000200013.

Espitia-Almeida, F., Díaz-Uribe, C., Vallejo, W., Gómez-Camargo, D., and Romero Bohórquez, A. R. (2020). In vitro anti-leishmanial effect of metallic meso-substituted porphyrin derivatives against leishmania braziliensis and leishmania panamensis promastigotes properties. *Molecules* 25, 1–11. doi:10.3390/molecules25081887.

Galeano, E., Martínez, A., Thomas, O. P., Robledo, S., and Munoz, D. (2012). Antiparasitic bromotyrosine derivatives from the caribbean marine sponge *Aiolochroia crassa*. *Quim. Nova* 35, 1189–1193. doi:10.1590/S0100-40422012000600023.

Galeano, E., Thomas, O. P., Robledo, S., Munoz, D., and Martinez, A. (2011). Antiparasitic bromotyrosine derivatives from the marine sponge *Verongula rigida*. *Mar. Drugs* 9, 1902–1913. doi:10.3390/md9101902.

García, E., Coa, J. C., Otero, E., Carda, M., Vélez, I. D., Robledo, S. M., et al. (2018). Synthesis and antiprotozoal activity of furanchalcone–quinoline, furanchalcone–chromone and furanchalcone–imidazole hybrids. *Med. Chem. Res.* 27, 497–511. doi:10.1007/s00044-017-2076-6.

Gómez-Ayala, S., Castrillón, J. A., Palma, A., Leal, S. M., Escobar, P., and Bahsas, A. (2010). Synthesis, structural elucidation and in vitro antiparasitic activity against Trypanosoma cruzi and Leishmania chagasi parasites of novel tetrahydro-1-benzazepine derivatives. *Bioorg. Med. Chem.* 18, 4721–4739. doi:10.1016/j.bmc.2010.05.018.

Granados-Falla, D., Gomez-Galindo, A., Daza, A., Robledo, S., Coy-Barrera, C., Cuca, L., et al. (2016). Seco-limonoid derived from Raputia heptaphylla promotes the control of cutaneous leishmaniasis in hamsters (Mesocricetus auratus). *Parasitology* 143, 289–299. doi:10.1017/S0031182015001717.

Hata, Y., De Mieri, M., Ebrahimi, S. N., Mokoka, T., Fouche, G., Kaiser, M., et al. (2014). Identification of two new phenathrenones and a saponin as antiprotozoal constituents of Drypetes gerrardii. *Phytochem. Lett.* 10, cxxxiii–cxl. doi:10.1016/j.phytol.2014.05.005.

Hernández-Chinea, C., Carbajo, E., Sojo, F., Arvelo, F., Kouznetsov, V. V, Romero-Bohórquez, A. R., et al. (2015). In vitro activity of synthetic tetrahydroindeno[2,1-c]quinolines on Leishmania mexicana. *Parasitol. Int.* 64, 479–483. doi:10.1016/j.parint.2015.06.011.

Hooker, J. D., Nguyen, V. H., Taylor, V. M., Cedeño, D. L., Lash, T. D., Jones, M. A., et al. (2012). New application for expanded porphyrins: Sapphyrin and heterosapphyrins as inhibitors of Leishmania parasites. *Photochem. Photobiol.* 88, 194–200. doi:10.1111/j.1751-1097.2011.01034.x.

Insuasty, B., Ramírez, J., Becerra, D., Echeverry, C., Quiroga, J., Abonia, R., et al. (2015). An efficient synthesis of new caffeine-based chalcones, pyrazolines and pyrazolo[3,4-b][1,4]diazepines as potential antimalarial, antitrypanosomal and antileishmanial agents. *Eur. J. Med. Chem.* 93, 401–413. doi:10.1016/j.ejmech.2015.02.040.

Insuasty, D., Robledo, S. M., Vélez, I. D., Cuervo, P., Insuasty, B., Quiroga, J., et al. (2017). A Schmidt rearrangement-mediated synthesis of novel tetrahydro-benzo[1,4]diazepin-5-ones as potential anticancer and antiprotozoal agents. *Eur. J. Med. Chem.* 141, 567–583. doi:10.1016/j.ejmech.2017.10.024.

Kouznetsov, V. V, Méndez, L. Y. V, Leal, S. M., Cruz, U. M., Coronado, C. A., Gómez, C. M. M., et al. (2007). Target-oriented synthesis of antiparasitic 2-hetaryl substituted quinolines based on imino Diels-Alder reactions. *Lett. Drug Des. Discov.* 4, 293–296. doi:10.2174/157018007784620031.

Leal, S. M., Amado, D. F., Kouznetsov, V. V, and Escobar, P. (2013a). In vitro antileishmanial, trypanocidal, and Mammalian cell activities of diverse n,n' -dihetaryl substituted diamines and related compounds. *Sci. Pharm.* 81, 43–55. doi:10.3797/scipharm.1205-14.

Leal, S. M., Pino, N., Stashenko, E. E., Martínez, J. R., and Escobar, P. (2013b). Antiprotozoal activity of essential oils derived from Piper spp. grown in Colombia. *J. Essent. Oil Res.* 25, 512–519. doi:10.1080/10412905.2013.820669.

Leañez, J., Nuñez, J., García-Marchan, Y., Sojo, F., Arvelo, F., Rodriguez, D., et al. (2019). Anti-leishmanial effect of spiro dihydroquinoline-oxindoles on volume regulation decrease and sterol biosynthesis of Leishmania braziliensis. *Exp. Parasitol.* 198, 31–38. doi:10.1016/j.exppara.2019.01.011.

López, R., Cuca, L. E., and Delgado, G. (2009). Antileishmanial and immunomodulatory activity of Xylopia discreta. *Parasite Immunol.* 31, 623–630. doi:10.1111/j.1365-3024.2009.01134.x.

Marin, F. J., Torres, O. L., Santafé, G. G., and Robledo, S. M. (2016). Estudio Fitoquímico y Evaluación del Potencial Leishmanicida de la Especie Esenbeckia litoralis (Rutaceae). *Inf. Tecnol.* 27, 159–168. doi:10.4067/S0718-07642016000100017.

Mesa V., A. M., Molano M., P. A., Seon, B., Figadere, B., Robledo, S. M., Muñoz, D. L., et al. (2008). Síntesis y actividades leishmanicida y citotóxica in vitro de análogos 2-arilquinolinas. *Vitae* 15, 259–266. Available at: https://doaj.org/article/61817657cf4c4a94a5d75122b8e7a5f0.

Mokoka, T. A., Xolani, P. K., Zimmermann, S., Hata, Y., Adams, M., Kaiser, M., et al. (2013). Antiprotozoal screening of 60 South African plants, and the identification of the antitrypanosomal germacranolides schkuhrin i and II. *Planta Med.* 79, 1380–1384. doi:10.1055/s-0033-1350691.

Murillo, J. A., Gil, J. F., Upegui, Y. A., Restrepo, A. M., Robledo, S. M., Quiñones, W., et al. (2019). Antileishmanial activity and cytotoxicity of ent-beyerene diterpenoids. *Bioorg. Med. Chem.* 27, 153–160. doi:10.1016/j.bmc.2018.11.030.

Ochoa, R., García, E., Robledo, S. M., and Cardona G, W. (2019). Virtual and experimental screening of phenylfuranchalcones as potential anti-Leishmania candidates. *J. Mol. Graph. Model.* 91, 164–171. doi:10.1016/j.jmgm.2019.06.015.

Ortiz, C., Echeverri, F., Robledo, S., Lanari, D., Curini, M., Quiñones, W., et al. (2020). Synthesis and Evaluation of Antileishmanial and Cytotoxic Activity of Benzothiopyrane Derivatives. *Molecules* 25. doi:10.3390/molecules25040800.

Osorio, E., Arango, G., Robledo, S., Muñoz, D., Jaramillo, L., and Vélez, I. (2006). Antileishmanial and cytotoxic activity of synthetic aromatic monoterpens. *Acta Farm. Bonaer.* 25, 405–413. Available at: https://www.scopus.com/inward/record.uri?eid=2-s2.0-33845383632&partnerID=40&md5=84a5054d7e89dacafcc70bc64f33fa5a.

Otero, E., García, E., Palacios, G., Yepes, L. M., Carda, M., Agut, R., et al. (2017). Triclosan-caffeic acid hybrids: Synthesis, leishmanicidal, trypanocidal and cytotoxic activities. *Eur. J.*

Med. Chem. 141, 73–83. doi:10.1016/j.ejmech.2017.09.064.
Otero, E., Robledo, S. M., Díaz, S., Carda, M., Muñoz, D., Paños, J., et al. (2014a). Synthesis and leishmanicidal activity of cinnamic acid esters: Structure-activity relationship. *Med. Chem. Res.* 23, 1378–1386. doi:10.1007/s00044-013-0741-y.
Otero, E., Vergara, S., Robledo, S. M., Cardona, W., Carda, M., Vélez, I. D., et al. (2014b). Synthesis, leishmanicidal and cytotoxic activity of triclosan-chalcone, triclosan-chromone and triclosan-coumarin hybrids. *Molecules* 19, 13251–13266. doi:10.3390/molecules190913251.
Pacheco, D. J., Trilleras, J., Quiroga, J., Gutiérrez, J., Prent, L., Coavas, T., et al. (2013). N-(4-((E)-3-arylacryloyl)phenyl)acetamide derivatives and their antileishmanial activity. *J. Braz. Chem. Soc.* 24, 1685–1690. doi:10.5935/0103-5053.20130203.
Palma, A., Yépes, A. F., Leal, S. M., Coronado, C. A., and Escobar, P. (2009). Synthesis and in vitro activity of new tetrahydronaphtho[1,2-b]azepine derivatives against Trypanosoma cruzi and Leishmania chagasi parasites. *Bioorganic Med. Chem. Lett.* 19, 2360–2363. doi:10.1016/j.bmcl.2008.05.013.
Peniche, A. G., Renslo, A. R., Melby, P. C., and Travi, B. L. (2015). Antileishmanial Activity of Disulfiram and Thiuram Disulfide Analogs in an Ex Vivo Model System Is Selectively Enhanced by the Addition of Divalent Metal Ions. *Antimicrob. Agents Chemother.* 59, 6463–6470. doi:10.1128/AAC.05131-14.
Pérez, J. M., Robledo, S., Cardona, W., Alzate, F., Muñoz, D., and Herrera, A. (2016). Leishmanicidal and cytotoxic activity of extracts and saponins from ilex laurina (Aquifoliaceae). *Trop. J. Pharm. Res.* 15, 973–979. doi:10.4314/tjpr.v15i5.11.
Quiliano, M., Pabón, A., Ramirez-Calderon, G., Barea, C., Deharo, E., Galiano, S., et al. (2017). New hydrazine and hydrazide quinoxaline 1,4-di-N-oxide derivatives: In silico ADMET, antiplasmodial and antileishmanial activity. *Bioorg. Med. Chem. Lett.* 27, 1820–1825. doi:10.1016/j.bmcl.2017.02.049.
Ramírez–Prada, J., Robledo, S. M., Vélez, I. D., Crespo, M. D. P., Quiroga, J., Abonia, R., et al. (2017). Synthesis of novel quinoline–based 4,5–dihydro–1H–pyrazoles as potential anticancer, antifungal, antibacterial and antiprotozoal agents. *Eur. J. Med. Chem.* 131, 237–254. doi:10.1016/j.ejmech.2017.03.016.
Restrepo, M. P., Jaramillo, E. G., Martínez, A. M., Arango, A. M., and Restrepo, S. R. (2018). Anti-parasite and cytotoxic activities of chloro and bromo L-tyrosine derivatives. *J. Braz. Chem. Soc.* 29, 2569–2579. doi:10.21577/0103-5053.20180136.
Restrepo, M. P., Surmay, V. S., Jaramillo, E. G., and Restrepo, S. R. (2019). Anti-Parasite Activity of Novel 3,5-Diiodophenethyl-benzamides. *J. Braz. Chem. Soc.* 30, 116–123. doi:10.21577/0103-5053.20180160.
Robledo, S. M., Cardona, W., Ligardo, K., Henao, J., Arbeláez, N., Montoya, A., et al. (2015). Antileishmanial Effect of 5,3'-Hydroxy-7,4'-dimethoxyflavanone of Picramnia gracilis Tul. (Picramniaceae) Fruit: In Vitro and In Vivo Studies. *Adv. Pharmacol. Sci.* 2015, 978379. doi:10.1155/2015/978379.
Robledo, S., Osorio, E., Muñoz, D., Jaramillo, L. M., Restrepo, A., Arango, G., et al. (2005). In vitro and in vivo cytotoxicities and antileishmanial activities of thymol and hemisynthetic derivatives. *Antimicrob. Agents Chemother.* 49, 1652–1655. doi:10.1128/AAC.49.4.1652-1655.2005.
Romero, I. C., Saravia, N. G., and Walker, J. (2005). Selective Action of Fluoroquinolones Against Intracellular Amastigotes of Leishmania (Viannia) Panamensis in Vitro. *J. Parasitol.* 91, 1474–1479. doi:10.1645/ge-3489.1.
Sánchez-Suárez, J., Coy-Barrera, E., Cuca, L. E., and Delgado, G. (2011). Leishmanicidal and cytotoxic activities of extracts and naturally-occurring compounds from two lauraceae species. *Nat. Prod. Commun.* 6. doi:10.1177/1934578x1100600218.
Sánchez, E. L., Santafé, G. G., Torres, O. L., Muñoz, D. L., and Robledo, S. M. (2014). Compuestos sintéticos del tipo de estirilquinolinas con actividades leishmanicida y citotóxica. *Biomedica* 34, 605–611. doi:10.7705/biomedica.v34i4.2299.
Taylor, V. M., Cedeño, D. L., Muñoz, D. L., Jones, M. A., Lash, T. D., Young, A. M., et al. (2011). In vitro and in vivo studies of the utility of dimethyl and diethyl carbaporphyrin ketals in treatment of cutaneous leishmaniasis. *Antimicrob. Agents Chemother.* 55, 4755–4764. doi:10.1128/AAC.00671-11.
Torres, O. L., Marin, F. J., Santafé, G. G., and Robledo, S. M. (2020). Sinthesis of styrylquinolines with Leishmanicidal potential in vitro on Leishmania (Viannia) panamensis. *Inf. Tecnol.* 31, 3–11. doi:10.4067/S0718-07642020000100003.
Upegui, Y., Gil, J. F., Quiñones, W., Torres, F., Escobar, G., Robledo, S. M., et al. (2014). Preparation of rotenone derivatives and in vitro analysis of their antimalarial, antileishmanial and selective cytotoxic activities. *Molecules* 19, 18911–18922. doi:10.3390/molecules191118911.
Upegui, Y., Rios, K., Quiñones, W., Echeverri, F., Archbold, R., Murillo, J. D., et al. (2019). Chroman-4-one hydrazones derivatives: synthesis, characterization, and in vitro and in vivo antileishmanial effects. *Med. Chem. Res.* 28, 2184–2199. doi:10.1007/s00044-019-02446-x.
Valdivieso, E., Mejías, F., Torrealba, C., Benaim, G., Kouznetsov, V. V, Sojo, F., et al. (2018). In vitro 4-Aryloxy-7-chloroquinoline derivatives are effective in mono- and combined therapy against Leishmania donovani and induce mitocondrial membrane potential disruption. *Acta Trop.* 183, 36–42. doi:10.1016/j.actatropica.2018.03.023.
Vargas, E., Echeverri, F., Upegui, Y. A., Robledo, S. M., and Quiñones, W. (2018). Hydrazone derivatives enhance antileishmanial activity of thiochroman-4-ones. *Molecules* 23.

doi:10.3390/molecules23010070.
Vargas, E., Echeverri, F., Vélez, I. D., Robledo, S. M., and Quiñones, W. (2017). Synthesis and evaluation of thiochroman-4-one derivatives as potential leishmanicidal agents. *Molecules* 22, 1–16. doi:10.3390/molecules22122041.
Vergara, S., Carda, M., Agut, R., Yepes, L. M., Vélez, I. D., Robledo, S. M., et al. (2017). Synthesis, antiprotozoal activity and cytotoxicity in U-937 macrophages of triclosan–hydrazone hybrids. *Med. Chem. Res.* 26, 3262–3273. doi:10.1007/s00044-017-2019-2.
Weininger, D. (1988). SMILES, a chemical language and information system. 1. Introduction to methodology and encoding rules. *J. Chem. Inf. Model.* 28, 31–36. doi:10.1021/ci00057a005.
Weniger, B., Vonthron-Sénécheau, C., Arango, G. J., Kaiser, M., Brun, R., and Anton, R. (2004). A bioactive biflavonoid from Campnosperma panamense. *Fitoterapia* 75, 764–767. doi:10.1016/j.fitote.2004.09.015.
Yépes, A. F., Bahsas, A., Escobar, P., Cobo, J., Palma, A., Garro Martinez, J. C., et al. (2018). Synthesis, anti-parasitic activity and QSAR study of a new library of polysubstituted tetrahydronaphtho[1,2-b]azepines. *Med. Chem. Res.* 27, 2239–2264. doi:10.1007/s00044-018-2232-7.

Table S3. Antileishmanial activity predicted by machine learning models.

ID	Exp	M1	M2	M3	M4
**1**	4.86	4.98	4.90	4.96	4.94
**2**	3.67	4.25	3.91	4.28	3.75
**3**	5.39	5.04	5.30	4.95	5.31
**5**	3.44	4.15	3.68	4.04	3.52
**6**	4.14	4.15	4.24	4.01	4.08
**7**	4.24	4.12	4.21	4.14	4.16
**8**	3.64	4.12	3.82	4.14	4.11
**9**	4.40	4.26	4.30	4.30	4.32
**10**	4.85	4.15	4.21	4.04	4.77
**11**	5.05	4.50	4.74	4.04	4.97
**12**	4.11	4.43	4.36	4.21	4.19
**32**	4.57	4.85	4.84	5.24	5.07
**33**	5.25	4.87	5.04	5.21	5.17
**34**	4.53	4.87	4.78	5.21	5.10
**35**	4.44	4.51	4.52	4.54	4.59
**36**	4.54	4.48	4.54	4.44	4.57
**37**	4.61	4.64	4.60	4.75	4.58
**38**	4.46	4.61	4.63	4.57	4.54
**39**	4.56	4.61	4.59	4.57	4.57
**40**	4.67	4.61	4.64	4.57	4.59
**41**	4.55	4.56	4.57	4.68	4.61
**44**	5.31	4.87	5.13	5.21	5.23
**98**	4.27	4.26	4.45	4.37	4.35
**99**	5.40	5.23	5.23	5.03	5.32
**100**	5.15	5.25	5.15	5.06	5.17
**101**	4.89	5.13	5.15	4.99	5.04
**102**	4.52	4.93	4.79	4.62	4.60
**103**	5.30	5.23	5.13	5.20	5.22
**104**	5.00	5.05	5.01	5.05	4.92
**105**	5.40	5.23	5.30	5.03	5.32
**106**	5.40	5.31	5.08	5.30	5.32
**107**	5.15	5.30	5.19	5.25	5.24
**108**	5.52	5.31	5.36	5.40	5.44
**109**	5.15	5.22	5.13	5.07	5.09
**110**	4.50	4.61	4.60	4.58	4.58
**111**	4.97	4.88	4.95	4.65	4.89
**112**	4.77	4.62	4.85	4.67	4.84

ID	Exp	M1	M2	M3	M4
**113**	4.80	4.77	4.88	4.88	4.88
**114**	4.69	4.74	4.80	4.79	4.77
**115**	5.19	4.76	5.00	4.71	5.11
**116**	4.99	4.61	4.86	4.58	4.91
**117**	4.38	4.61	4.44	4.58	4.46
**118**	4.75	4.88	4.77	4.65	4.72
**119**	4.70	4.62	4.64	4.67	4.69
**120**	4.98	4.77	4.62	4.88	4.90
**121**	4.84	4.74	4.74	4.79	4.76
**122**	4.38	4.76	4.49	4.71	4.46
**123**	4.27	4.61	4.46	4.58	4.69
**125**	2.57	3.01	3.07	2.67	2.65
**126**	3.61	3.43	3.53	3.51	3.69
**127**	3.16	3.20	3.24	3.26	3.24
**128**	2.85	3.12	2.93	2.95	2.93
**129**	3.34	3.59	3.38	3.43	3.42
**130**	3.86	3.75	3.21	3.94	3.78
**131**	3.71	3.44	3.64	3.61	3.63
**132**	3.26	3.31	3.27	3.48	3.34

ID	Exp	M1	M2	M3	M4
**133**	4.19	3.80	3.74	4.09	4.11
**134**	4.27	4.01	4.11	3.85	4.20
**135**	3.90	4.54	4.02	4.00	3.98
**136**	3.90	4.02	4.06	4.00	3.98
**137**	3.89	4.38	4.11	3.99	3.97
**138**	3.90	3.91	3.95	3.73	4.13
**139**	3.90	3.88	3.94	3.80	4.16
**140**	4.90	4.61	4.67	4.37	4.22
**141**	3.92	3.89	3.97	4.02	4.30
**142**	4.79	4.25	4.33	4.33	4.31
**143**	3.92	3.98	4.12	3.96	4.00
**144**	4.19	4.30	4.34	4.29	4.50
**145**	3.96	4.67	4.01	4.47	4.04
**146**	3.87	3.98	3.84	4.12	3.86
**147**	3.42	3.94	3.75	3.52	3.50
**148**	4.03	4.19	4.20	4.36	4.25
**149**	4.45	4.22	4.39	4.35	4.37
**150**	4.01	4.17	4.10	4.11	4.09
**151**	4.03	4.13	4.13	4.13	4.11

ID	Exp	M1	M2	M3	M4
**152**	4.18	4.16	4.17	4.21	4.23
**153**	4.13	4.14	4.23	4.23	4.21
**154**	4.49	4.14	4.10	4.23	4.41
**155**	4.17	4.11	4.14	4.08	4.17
**156**	6.80	6.22	6.38	6.08	5.74
**157**	6.28	6.23	6.19	6.18	6.15
**158**	5.78	6.01	5.85	5.92	5.86
**159**	6.66	6.11	6.39	6.00	6.58
**160**	6.22	6.22	6.28	6.08	5.91
**161**	5.82	6.01	5.85	5.92	5.86
**162**	5.90	6.11	5.90	6.00	5.88
**163**	5.85	6.01	6.01	5.92	5.93
**164**	6.15	6.11	6.09	6.00	6.22
**165**	7.15	6.22	6.38	6.08	5.68
**166**	6.17	6.01	6.20	5.92	6.21
**167**	5.85	6.00	5.97	5.80	5.77
**168**	6.15	5.48	5.72	5.49	5.55
**169**	5.47	5.48	5.72	5.49	5.55
**170**	4.19	4.48	4.31	4.71	4.27
**171**	4.10	5.48	5.72	5.49	5.55
**172**	4.96	4.61	4.33	4.62	4.88
**173**	5.68	6.11	5.77	6.00	5.76
**174**	5.66	6.11	5.77	6.00	5.76
**175**	5.95	6.11	5.77	6.00	5.76
**176**	5.77	6.11	5.77	6.00	5.76
**177**	4.95	5.29	5.42	5.21	5.03
**178**	5.78	5.29	5.42	5.21	5.03
**179**	5.67	5.29	5.42	5.21	5.03
**180**	5.68	6.11	6.07	6.00	6.01
**181**	6.53	6.11	6.07	6.00	6.01
**182**	6.00	6.11	6.07	6.00	6.01
**183**	6.09	6.11	6.07	6.00	6.01
**184**	4.26	4.10	4.06	4.16	4.01
**185**	4.08	4.07	4.14	4.03	4.14
**186**	3.94	4.09	3.95	3.99	4.02
**187**	3.82	3.98	4.08	3.92	3.90
**188**	3.82	4.05	3.90	3.92	3.90
**189**	3.15	3.81	3.57	3.25	3.23

ID	Exp	M1	M2	M3	M4
**190**	3.30	4.08	3.85	3.40	3.38
**191**	6.24	5.56	5.52	5.24	6.16
**192**	5.13	5.08	5.15	5.03	5.05
**194**	4.86	4.98	4.90	4.96	4.94
**195**	3.67	4.25	3.91	4.28	3.75
**196**	5.39	5.04	5.30	4.95	5.31
**197**	3.92	3.96	3.81	3.87	3.98
**198**	3.97	3.96	3.79	3.87	3.89
**199**	3.84	3.79	3.63	3.74	3.76
**200**	2.95	3.79	3.31	3.74	3.68
**201**	3.52	3.77	3.61	3.62	3.60
**202**	3.40	3.77	3.67	3.62	3.48
**203**	3.81	3.60	3.06	3.56	3.73
**204**	3.22	3.60	3.36	3.56	3.30
**205**	3.62	3.58	3.64	3.56	3.70
**206**	3.38	3.38	3.37	3.28	3.39
**207**	3.17	3.38	3.29	3.28	3.25
**208**	3.61	3.45	3.55	3.56	3.69
**209**	3.69	3.45	3.63	3.56	3.61

ID	Exp	M1	M2	M3	M4
**210**	2.68	3.21	2.94	3.26	3.64
**211**	3.29	3.21	3.32	3.26	3.33
**212**	3.21	3.48	3.36	3.29	3.29
**213**	3.59	3.48	3.67	3.29	3.67
**214**	4.10	3.62	3.87	3.56	4.02
**215**	3.46	3.62	3.62	3.56	4.01
**230**	3.97	4.25	4.54	4.36	4.05
**231**	4.35	4.31	4.39	4.41	4.17
**232**	3.90	4.32	4.36	4.44	4.26
**233**	4.36	4.32	4.36	4.44	4.26
**234**	4.34	4.32	4.36	4.44	4.26
**235**	4.75	4.32	4.36	4.44	4.26
**236**	5.33	5.20	5.04	4.61	5.25
**237**	4.66	5.17	4.88	4.76	4.85
**238**	4.78	5.28	5.13	4.98	5.00
**239**	5.08	5.28	5.13	4.98	5.00
**240**	6.11	5.28	5.13	4.98	5.00
**241**	5.67	5.28	5.13	4.98	5.00
**242**	4.47	4.53	4.34	4.57	4.55

ID	Exp	M1	M2	M3	M4
**243**	4.86	4.53	4.24	4.58	4.56
**244**	4.23	4.53	4.24	4.58	4.56
**245**	4.68	4.53	4.24	4.58	4.56
**246**	3.68	4.53	4.24	4.58	4.56
**247**	4.15	4.70	4.30	4.25	4.07
**248**	5.61	4.59	5.13	4.46	5.35
**249**	3.14	3.87	3.63	3.46	3.22
**250**	5.21	4.72	4.82	5.11	5.13
**251**	3.90	4.54	4.22	4.00	4.32
**252**	5.59	4.87	5.21	4.47	5.50
**253**	5.08	5.06	4.91	4.31	5.00
**254**	4.53	4.56	4.66	4.43	4.61
**255**	4.62	4.61	4.68	4.58	4.69
**256**	4.53	4.55	4.55	4.50	4.55
**257**	4.43	4.52	4.41	4.53	4.51
**258**	5.04	4.75	4.80	4.94	4.96
**259**	4.36	4.66	4.51	4.48	4.44
**260**	4.42	4.51	4.44	4.52	4.48
**261**	4.42	4.69	4.45	4.52	4.55

ID	Exp	M1	M2	M3	M4
**262**	3.97	3.83	4.03	4.07	4.20
**263**	3.87	4.36	3.90	3.97	3.95
**264**	4.90	5.11	4.22	4.25	4.82
**265**	3.38	3.59	3.85	3.48	3.38
**266**	2.45	3.29	3.00	3.34	2.53
**267**	3.31	3.44	3.36	3.36	3.39
**268**	3.56	3.44	3.60	3.36	3.64
**269**	4.16	3.73	4.09	3.79	4.08
**270**	3.72	3.84	3.88	3.62	3.80
**271**	3.99	3.91	3.90	3.74	3.92
**272**	3.84	3.91	3.90	3.74	3.92
**273**	3.67	3.96	3.92	3.77	4.17
**274**	4.10	3.96	3.92	3.77	4.17
**275**	4.00	4.11	4.03	4.25	4.03
**276**	3.30	3.59	3.85	3.48	3.38
**277**	4.27	3.59	3.85	3.48	3.38
**278**	3.43	3.84	3.88	3.62	3.80
**279**	4.28	3.84	3.88	3.62	3.80
**280**	4.55	4.33	4.42	4.45	4.35

ID	Exp	M1	M2	M3	M4
**281**	4.27	4.40	4.22	4.36	4.35
**282**	3.72	3.87	3.95	3.82	3.80
**296**	5.04	5.36	4.96	5.14	4.96
**297**	5.69	5.36	4.96	5.14	4.96
**298**	3.35	3.51	3.42	3.45	3.43
**299**	3.37	3.43	3.39	3.48	3.33
**300**	3.96	3.76	3.96	3.86	3.88
**301**	4.10	3.97	4.00	4.00	4.04
**302**	4.02	3.90	3.98	4.07	4.05
**303**	3.95	3.87	3.93	4.05	3.87
**304**	4.36	4.19	4.30	3.94	4.28
**305**	4.21	4.12	4.19	4.11	4.25
**306**	4.10	4.13	4.00	4.20	4.02
**307**	4.14	4.33	4.18	4.05	4.31
**308**	4.03	4.14	4.09	4.01	4.11
**309**	4.15	4.14	4.11	4.24	4.23
**310**	4.43	4.21	4.34	4.27	4.35
**311**	3.79	4.11	3.96	4.03	3.87
**312**	4.28	4.13	4.09	4.18	4.07

ID	Exp	M1	M2	M3	M4
**313**	4.13	4.33	4.20	4.06	4.14
**314**	4.11	4.15	4.08	4.01	4.03
**315**	4.15	4.20	4.14	4.25	4.22
**316**	4.02	3.84	3.99	3.92	3.94
**317**	4.06	3.74	4.03	3.97	3.97
**318**	4.03	3.88	4.01	4.06	3.98
**319**	3.66	3.98	3.91	3.92	3.74
**320**	4.07	3.99	4.06	4.15	4.05
**321**	4.10	4.02	4.08	4.20	4.15
**322**	3.86	4.31	4.12	3.96	4.03
**323**	4.16	4.01	4.02	4.05	4.08
**324**	4.20	4.03	4.12	4.30	4.13
**325**	5.12	5.01	5.02	5.02	5.04
**326**	5.14	4.98	5.13	5.20	4.87
**327**	5.49	4.96	5.23	5.27	4.58
**328**	5.13	4.96	5.06	5.03	5.05
**329**	5.17	4.96	5.13	5.14	5.09
**330**	5.14	5.06	5.10	5.04	5.06
**331**	3.69	3.83	3.74	3.61	3.77

ID	Exp	M1	M2	M3	M4
**332**	3.54	3.80	3.74	3.64	3.54
**333**	4.05	4.22	4.17	4.30	4.13
**334**	4.12	4.03	3.98	4.02	4.04
**335**	3.80	4.03	4.18	4.21	3.88
**336**	3.73	4.02	3.87	3.83	3.81
**341**	5.32	4.96	5.18	4.74	5.24
**343**	5.21	4.90	5.10	4.81	5.13
**345**	5.23	5.16	4.65	5.13	4.53
**368**	5.89	5.02	4.89	4.81	5.81
**369**	4.61	4.70	4.60	4.63	4.69
**370**	4.60	4.70	4.98	4.63	4.68
**371**	5.79	5.00	5.39	4.84	5.71
**372**	4.15	5.02	4.40	4.81	4.23
**373**	5.16	5.13	4.99	4.90	5.08
**374**	5.63	5.19	5.36	5.01	5.55
**375**	4.41	4.71	4.56	4.62	4.49
**376**	4.92	4.86	4.90	4.82	4.86
**377**	5.03	5.17	4.88	5.02	4.83
**378**	4.34	5.00	4.54	4.84	4.42

ID	Exp	M1	M2	M3	M4
**393**	4.28	4.73	4.73	4.76	4.36
**394**	5.03	4.84	4.82	4.77	4.95
**395**	4.82	4.81	4.78	4.76	4.76
**396**	4.87	4.84	4.69	4.77	4.79
**397**	4.63	4.73	4.09	4.76	4.54
**398**	3.74	4.06	3.93	3.84	3.82
**399**	3.14	3.95	3.53	3.86	3.33
**400**	5.19	4.84	5.01	4.83	4.65
**401**	5.05	4.90	4.89	4.81	4.97
**402**	4.72	4.89	4.78	4.82	4.80
**403**	5.31	4.84	5.13	4.83	5.23
**404**	4.61	4.80	4.72	4.51	4.69
**405**	5.22	4.82	4.73	4.77	5.14
**450**	4.05	4.11	4.06	4.15	4.07
**451**	3.97	4.14	4.05	4.34	4.05
**452**	3.96	4.14	4.02	4.34	4.04
**453**	4.27	4.18	4.30	4.19	4.24
**454**	4.35	4.23	4.35	4.39	4.27
**455**	4.49	4.23	4.38	4.39	4.26

ID	Exp	M1	M2	M3	M4
**456**	4.12	4.13	4.11	4.02	4.13
**457**	4.22	4.14	4.17	4.12	4.19
**458**	4.01	4.15	4.08	4.10	4.19
**459**	3.95	4.15	3.94	4.05	4.03
**460**	4.17	4.16	4.13	4.07	4.16
**461**	3.19	4.07	3.49	3.44	4.06
**479**	5.47	4.87	5.27	5.32	4.91
**480**	5.72	5.37	5.44	5.53	5.64
**481**	5.72	5.41	5.61	5.62	5.64
**482**	5.19	5.26	5.31	5.12	5.21
**483**	4.90	5.01	5.15	5.00	4.98
**484**	5.52	5.36	5.48	5.47	5.44
**485**	5.18	5.37	5.56	5.53	5.26
**486**	4.93	5.19	4.86	5.18	5.01
**487**	5.15	5.19	5.21	5.25	5.23
**488**	5.20	5.18	5.13	5.10	5.12
**489**	5.43	5.17	5.25	5.26	4.93
**490**	5.47	5.20	5.36	5.37	5.39
**491**	5.08	5.19	5.24	5.18	5.16

ID	Exp	M1	M2	M3	M4
**492**	3.21	4.63	3.85	5.24	4.50
**493**	5.30	4.92	5.13	5.35	5.22
**494**	4.14	4.81	4.46	4.77	4.22
**495**	4.88	4.87	4.79	4.98	4.80
**496**	5.30	4.86	5.09	5.20	5.22
**497**	5.39	4.63	5.01	5.24	5.31
**498**	4.64	4.35	4.55	4.58	4.72
**499**	5.19	4.80	5.09	4.77	5.27
**500**	4.86	4.53	4.80	4.76	4.83
**501**	4.62	4.61	4.69	4.55	4.70
**502**	5.43	4.59	5.13	4.58	5.21
**503**	4.32	4.35	4.54	4.58	4.88
**504**	3.77	4.35	4.04	4.58	3.93
**505**	4.56	4.80	4.43	4.77	4.57
**506**	4.29	4.53	4.23	4.76	4.21
**507**	4.45	4.61	4.31	4.55	4.24
**508**	4.02	4.59	4.15	4.58	4.10
**509**	4.51	4.35	4.37	4.58	4.43
**510**	4.68	4.35	4.30	4.58	4.55

ID	Exp	M1	M2	M3	M4
**511**	5.35	4.80	5.06	4.77	5.27
**512**	4.66	4.53	4.63	4.76	4.58
**513**	4.58	4.61	4.60	4.55	4.51
**514**	4.49	4.59	4.70	4.58	4.57
**515**	4.48	4.35	4.55	4.58	4.56
**547**	4.36	4.30	4.29	4.26	4.28
**548**	4.16	4.23	4.18	4.10	4.24
**549**	3.84	4.00	3.97	3.94	3.92
**550**	3.94	4.09	4.17	4.04	4.02
**551**	4.12	4.26	4.16	4.22	4.20
**552**	4.26	4.27	4.19	4.36	4.34
**553**	3.32	3.82	3.92	4.11	3.40
**554**	4.00	3.81	3.93	3.97	3.95
**555**	3.81	3.83	3.87	4.01	3.89
**556**	3.42	3.81	3.64	3.97	3.50
**557**	4.21	3.82	4.18	4.11	4.13
**558**	4.81	4.63	4.79	4.72	4.74
**559**	4.79	4.63	4.75	4.69	4.71
**560**	4.68	4.55	4.57	4.62	4.60

ID	Exp	M1	M2	M3	M4
**561**	4.53	4.55	4.57	4.62	4.60
**562**	4.40	4.50	4.44	4.40	4.51
**563**	4.28	4.48	4.36	4.42	4.35
**564**	4.42	4.46	4.40	4.36	4.38
**565**	4.46	4.46	4.40	4.36	4.38
**566**	5.26	4.67	5.00	4.72	5.13
**567**	4.83	4.63	4.74	4.73	4.75
**568**	4.51	4.61	4.60	4.63	4.53
**569**	4.45	4.61	4.60	4.63	4.53
**570**	4.60	4.65	4.81	4.70	4.68
**571**	4.89	4.67	4.79	4.69	4.81
**572**	4.60	4.61	4.54	4.63	4.68
**573**	4.80	4.61	4.54	4.63	4.68
**574**	4.53	4.61	4.54	4.63	4.68
**575**	4.43	4.61	4.54	4.63	4.68
**576**	4.77	4.47	4.44	4.59	4.42
**577**	3.89	4.44	4.37	4.57	4.37
**578**	4.29	4.44	4.37	4.57	4.37
**579**	4.77	4.44	4.37	4.57	4.37

ID	Exp	M1	M2	M3	M4
**580**	4.47	4.44	4.37	4.57	4.37
**581**	4.16	4.43	4.77	4.26	4.24
**582**	4.67	4.53	4.65	4.26	4.26
**583**	5.58	4.82	5.19	4.81	4.38
**584**	3.92	4.55	4.28	4.33	4.00
**585**	4.32	4.64	4.45	4.63	4.24
**586**	4.49	4.44	4.49	4.21	4.44
**587**	4.79	4.71	4.70	4.69	4.71
**588**	4.19	4.43	4.28	4.29	4.27
**589**	4.21	4.55	4.28	4.51	4.41
**590**	4.96	4.82	4.95	4.81	4.88
**637**	4.61	4.76	4.84	4.71	4.69
**638**	5.42	4.83	5.15	4.78	4.95
**639**	4.82	4.72	4.72	4.72	4.81
**640**	4.93	4.72	4.72	4.72	4.81
**641**	4.89	4.72	4.72	4.72	4.81
**642**	4.52	4.72	4.72	4.72	4.81
**643**	4.37	4.72	4.72	4.72	4.81
**644**	4.42	4.45	4.41	4.50	4.34

ID	Exp	M1	M2	M3	M4
**645**	4.27	4.46	4.35	4.48	4.27
**646**	4.00	4.38	4.16	4.42	4.08
**647**	4.31	4.38	4.16	4.42	4.08
**648**	4.40	4.38	4.63	4.20	4.46
**666**	3.96	3.76	3.96	3.86	3.88
**667**	3.35	3.51	3.42	3.45	3.43
**668**	3.37	3.43	3.39	3.48	3.33
**669**	4.36	4.19	4.30	3.94	4.28
**670**	4.25	4.21	4.20	4.19	4.17
**671**	4.04	4.04	4.16	4.05	4.00
**672**	4.25	4.08	4.20	4.15	4.17
**673**	4.43	4.24	4.33	4.41	4.26
**674**	4.40	4.23	4.28	4.30	4.32
**675**	4.02	4.06	4.10	4.12	4.10
**676**	4.42	4.27	4.32	4.32	4.34
**677**	4.25	4.25	4.17	4.18	4.22
**678**	4.04	4.09	4.09	3.99	4.12
**679**	4.36	4.25	4.29	4.26	4.28
**680**	4.00	4.23	4.07	4.11	4.08

ID	Exp	M1	M2	M3	M4
**681**	3.79	4.06	4.01	3.89	3.87
**701**	3.33	3.59	3.35	3.43	3.41
**704**	4.24	4.55	4.37	4.33	4.32
**706**	4.95	4.59	4.69	4.85	4.87
**732**	3.76	4.75	4.15	4.51	3.84
**733**	4.34	4.45	4.50	4.43	4.42
**734**	5.59	5.48	5.72	5.49	5.55
**735**	5.02	5.33	4.94	5.21	4.94
**736**	4.66	4.74	4.48	4.59	4.30
**737**	5.17	4.71	4.81	4.57	5.09
**738**	4.41	4.75	4.49	4.51	4.36
**739**	4.43	4.69	4.79	4.53	4.85
**740**	5.02	4.70	4.92	4.60	4.94
**741**	5.34	5.04	5.31	5.09	5.16
**742**	3.86	4.40	4.16	4.06	3.94
**743**	4.95	5.70	4.90	5.05	4.87
**744**	5.95	6.11	6.07	6.00	6.01
**745**	4.32	4.57	4.51	4.64	4.40
**746**	5.24	5.59	5.43	5.34	5.32

ID	Exp	M1	M2	M3	M4
**747**	5.21	5.29	5.29	5.21	5.13
**748**	4.46	5.67	4.78	5.45	4.54
**749**	5.27	5.70	5.60	5.67	5.35
**750**	5.19	5.23	4.93	5.16	4.65
**751**	3.92	4.37	4.75	4.02	4.00
**752**	5.31	5.21	5.02	5.21	5.23
**753**	5.68	5.23	5.39	5.16	5.60
**754**	3.81	4.45	4.36	4.16	3.89
**755**	4.78	4.62	4.68	4.68	4.70
**756**	4.20	4.47	4.43	4.30	4.28
**757**	4.30	4.45	4.33	4.20	4.38
**758**	5.66	5.24	5.32	5.30	5.58
**759**	4.63	5.20	4.77	5.35	4.71
**775**	4.20	4.68	4.38	4.66	4.28
**776**	5.04	4.82	4.43	5.01	4.96
**777**	5.59	5.31	5.48	5.49	5.51
**778**	5.55	5.31	5.48	5.49	5.51
**779**	5.64	5.31	5.52	5.49	5.27
**780**	4.64	4.92	4.69	4.75	4.63

ID	Exp	M1	M2	M3	M4
**804**	6.22	4.88	5.66	5.41	6.14
**805**	5.54	4.86	5.29	5.44	5.46
**806**	5.26	4.84	5.11	5.16	5.34
**807**	5.01	4.71	4.94	4.34	5.09
**808**	3.92	4.17	4.57	4.02	5.22
**809**	3.82	4.17	4.49	4.02	3.90
**810**	3.67	3.92	4.51	3.77	3.75
**811**	4.98	4.24	4.72	4.72	4.90
**812**	3.25	3.69	3.63	3.48	3.33
**813**	3.96	3.84	4.02	3.91	3.93
**814**	4.48	3.97	4.09	4.38	4.18

* M1: RF with MACCS; M2: RF with Morgan; M3: SVM with MACCS; M4: SVM with Morgan. Gray cells for samples in test set. Activity expressed as pEC_{50}

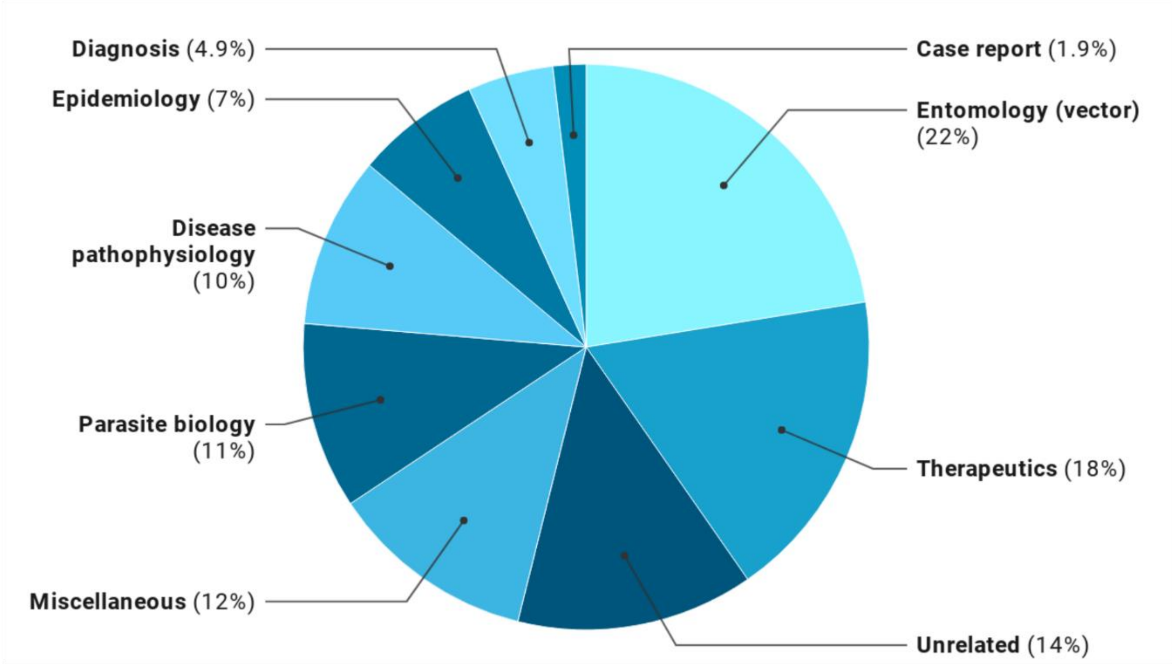

Figure S1. Distribution of the Colombian scientific literature on leishmaniasis.

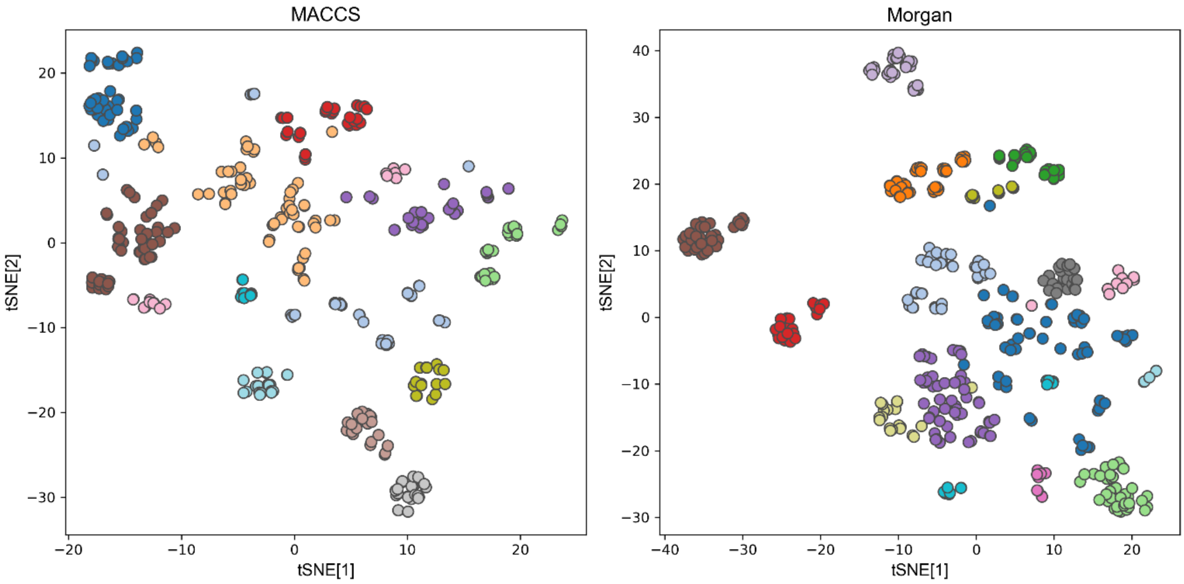

Figure S2. t-SNE plot using MACCS (left) and Morgan (right) fingerprints colored by HCA. Colors represent different clusters in each case (13 with MACCS and 16 with Morgan).

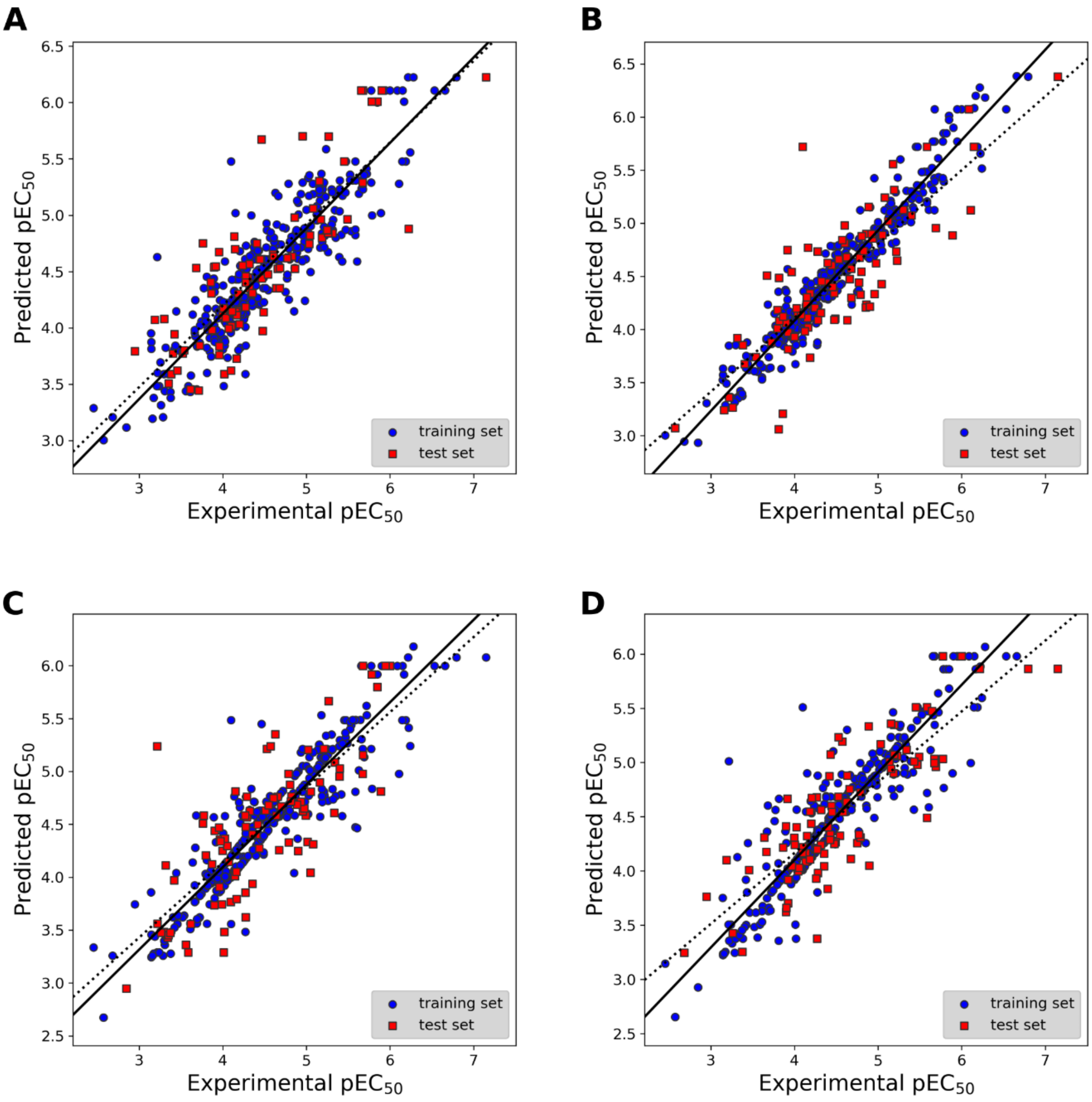

Figure S3. Experimental versus predicted activity (pEC$_{50}$) for machine learning models. (A) M1 (RF with MACCS), (B) M2 (RF with Morgan), (C) M3 (SVM with MACCS), and (D) M4 (SVM with Morgan).
